# Benchmarking algorithms for joint integration of unpaired and paired single-cell RNA-seq and ATAC-seq data

**DOI:** 10.1186/s13059-023-03073-x

**Published:** 2023-10-24

**Authors:** Michelle Y. Y. Lee, Klaus H. Kaestner, Mingyao Li

**Affiliations:** 1 Department of Genetics, University of Pennsylvania, Philadelphia, PA 19104 USA; 2 Graduate Group in Genomics and Computational Biology, University of Pennsylvania Perelman School of Medicine, Philadelphia, Philadelphia, PA 19104 USA; 3 Department of Biostatistics, Epidemiology and Informatics, Perelman School of Medicine, University of Pennsylvania, Philadelphia, PA USA

## Abstract

**Background:**

Single-cell RNA-sequencing (scRNA-seq) measures gene expression in single cells, while single-nucleus ATAC-sequencing (snATAC-seq) quantifies chromatin accessibility in single nuclei. These two data types provide complementary information for deciphering cell types and states. However, when analyzed individually, they sometimes produce conflicting results regarding cell type/state assignment. The power is compromised since the two modalities reflect the same underlying biology. Recently, it has become possible to measure both gene expression and chromatin accessibility from the same nucleus. Such paired data enable the direct modeling of the relationships between the two modalities. Given the availability of the vast amount of single-modality data, it is desirable to integrate the paired and unpaired single-modality datasets to gain a comprehensive view of the cellular complexity.

**Results:**

We benchmark nine existing single-cell multi-omic data integration methods. Specifically, we evaluate to what extent the multiome data provide additional guidance for analyzing the existing single-modality data, and whether these methods uncover peak-gene associations from single-modality data. Our results indicate that multiome data are helpful for annotating single-modality data. However, we emphasize that the availability of an adequate number of nuclei in the multiome dataset is crucial for achieving accurate cell type annotation. Insufficient representation of nuclei may compromise the reliability of the annotations. Additionally, when generating a multiome dataset, the number of cells is more important than sequencing depth for cell type annotation.

**Conclusions:**

Seurat v4 is the best currently available platform for integrating scRNA-seq, snATAC-seq, and multiome data even in the presence of complex batch effects.

**Supplementary Information:**

The online version contains supplementary material available at 10.1186/s13059-023-03073-x.

## Background

Over the past 10 years, hundreds of single-cell RNA-seq (scRNA-seq) (for transcript abundance in single cells) or single-nucleus ATAC-seq (snATAC-seq) (for chromatin accessibility in single nuclei) datasets have been produced by laboratories worldwide, leading to the discovery of new cell types and regulatory circuits. In addition, by applying single-cell assays to two-state models such as the comparison between control and mutant tissues, changes in gene expression or chromatin accessibility caused by a gene mutation could be analyzed easily at the cell type-specific level for the first time. Unfortunately, each single-modality dataset measures either the gene expression or the chromatin accessibility of a given cell. Although the two datasets can be generated from the same cell population, they measure different individual cells. Most of the time, the two experimental modalities result in the identification of similar cell types, as the highly expressed genes used to define cell types at the transcript levels frequently have promoters that are identified as highly accessible by the ATAC-seq modality. However, there are situations when the two profiles are discordant. In these situations, simultaneous, joint profiling of gene expression and chromatin accessibility is paramount for resolving inconsistency and revealing novel cell types and states that show modality-specific features. Moreover, the joint profiling of gene expression and chromatin accessibility of the same exact cells offers the most direct link between *cis*-regulatory elements and their target genes [1].

Recently, it has become feasible to simultaneously determine both transcript levels and chromatin state in the same nucleus, using so-called “multi-omics” approaches. An example is the 10 × Genomics Single Cell Multiome ATAC + Gene Expression technology [2]. Multi-omics data are superior at refining cell types and revealing gene regulatory networks [1]. However, it is not practical to repeat all prior studies of interest performed using the single-modality assays with the multiome approaches, as frequently precious samples are either no longer available or funding is limited. Therefore, it is highly desirable to integrate pre-existing single-modality scRNA-seq and snATAC-seq datasets with multiome data generated subsequently using the newer technology to achieve more accurate cell type annotations.

Several methodologies have been developed for multi-omic data integration. Here, we refer to multi-omic integration as the integration of RNA-seq and ATAC-seq profiles measured in single cells or nuclei, either with or without the guidance of multiome data. These methods attempt to align cells profiled by separate technologies and project them into one common low-dimensional space to ensure consistent cell type calling. However, we still lack an objective evaluation of whether the addition of multiome data improves the annotation of single-modality datasets. Furthermore, some of the methods attempt to impute the missing modality for the single-modality datasets and identify peak-gene pairs using these “pseudo-paired” datasets. Thus, it is still uncertain if the imputed missing modality can truly provide additional biological insights to the same degree as provided by the experimentally produced multiome datasets. Finally, given the availability of many methods for multi-omic data integration, at present, we do not know which method performs the best when integrating all three data types.

The current multi-omic integration methods can be divided into two categories. Methods in the first category perform multi-omic integration using only the single-modality datasets, aiming to find a mapping between gene expression profiles and chromatin accessibility states to create an aligned space that explains both modalities; we call these approaches “unpaired integration.” Representative methods in this category include Seurat version 3 (Seurat v3) [3], which performs canonical correlation analysis (CCA) to align experimentally measured gene expression with pseudo-gene expression obtained from chromatin accessibility. One example of pseudo-gene expression is the gene activity score, calculated by summing up peak counts within the gene body plus 2 kb upstream in the ATAC-seq data. LIGER [4] also uses gene expression and activity scores to obtain shared features between the two modalities and then derives a low-dimensional embedding through a non-negative matrix factorization approach. FigR [5] aligns the snATAC-seq and scRNA-seq data using a CCA-based approach. In addition, it provides matching of snATAC-seq and scRNA-seq cells, which enables the identification of *cis*-regulatory elements similarly to what can be achieved with paired multiome data. BindSC [6] goes beyond the simple construction of gene activity scores. Instead, bindSC uses a bi-directional CCA to empirically construct a cell-by-gene matrix for the snATAC-seq cells that preserve its similarity with the ATAC-seq input and simultaneously maximizes the correlation with the scRNA-seq matrix it is being integrated with. A recently developed method called GLUE [7] uses a deep-learning approach called “variational autoencoder” to extract features for each modality. To link the features across modalities, GLUE requests a knowledge-based guidance graph, which links genomic regions to genes based on their genomic proximity. Using the knowledge graph and the autoencoder system, GLUE learns the representation of cells from different modalities and aligns them through an iterative process.

Methods in the second category encompass more recent approaches that incorporate information from multiome cells and integrate all three data types for a more comprehensive exploration of cellular identities; we term these approaches “multiome-guided integration.” Representative methods in this category include Seurat version 4 (Seurat v4) [8], an approach that first learns a low-dimensional representation of the cells profiled by the multiome methodology using both the RNA-seq and ATAC-seq profiles by weighted nearest neighbors (WNN) analysis [8]. Subsequently, the two single-modality datasets are projected onto the WNN embedding space in a supervised manner. MultiVI [9] and Cobolt [10] also use “variational autoencoders” to embed all three data types. Both methods employ the encoder-decoder system to learn a low-dimensional representation of the data. Specifically, two encoders and two decoders are set up, one for each modality. However, the two platforms employ different model choices. MultiVI assumes a negative binomial distribution for the RNA-seq data and a Bernoulli distribution for the ATAC-seq data, while Cobolt assumes a Multivariate Normal distribution for both modalities. Furthermore, the two methods integrate the modality-specific representation for the paired cells differently. MultiVI first aligns the two embeddings through a symmetric Kullback–Leibler (KL) divergence loss and then obtains an average of the two embeddings. On the other hand, Cobolt simply multiplies the two embeddings to represent the paired cells, while the representation of the unpaired cells is first generated by the corresponding encoder and refined using a linear transformation to ensure enough similarity between the RNA-seq derived embedding and the ATAC-seq derived embedding. We included another method called scMoMaT [11]. We chose this method to represent a broad category of methods called “mosaic integration,” which can integrate datasets that differ in either cells, features, or both [12]. In addition, these methods could integrate situations where there are three or more modalities. We acknowledge that the situations benchmarked here do not represent these methods’ full capacity, but we wanted to include at least one method from this category to represent their performance in this specific situation where we integrate scRNA-seq, snATAC-seq, and multiome datasets. ScMoMaT employs a matrix tri-factorization framework, which decomposes each count matrix into a cell matrix, a feature matrix, and, finally, an association matrix that captures the strength between the cell and feature matrices.

All methods described above aim to project cells from different data types into one shared space to facilitate the identification of cell types through clustering. Nevertheless, a common goal for studies profiling chromatin accessibility and gene expression at the single-cell level is to understand cell type-specific *cis*-regulatory logic. Since the two single-modality datasets are generated from different cells in a given population, albeit representing the same cell types, the single-modality datasets cannot be naïvely combined to test for association between chromatin accessibility and gene expressions. Therefore, multiple efforts have attempted to impute the missing modality for the single-modality datasets, aiming to computationally generate paired profiles similar to those measured experimentally by the multiome technology. Some methods mentioned above, e.g., Seurat v3, FigR, bindSC, Seurat v4, and MultiVI, are capable of this task. However, an objective evaluation of how reliable the in silico imputed profiles are compared to what is directly measured by the paired multiome technologies is still lacking. Therefore, we aimed to conduct an extensive benchmarking analysis to evaluate the abovementioned methods by addressing two important questions. First, do multiome data improve the integration of single-modality datasets? Second, what is the best computational method for the integration of scRNA-seq, snATAC-seq, and multiome data?

## Results

### Overview of the benchmarking scheme and evaluation strategies

**Fig. 1:**
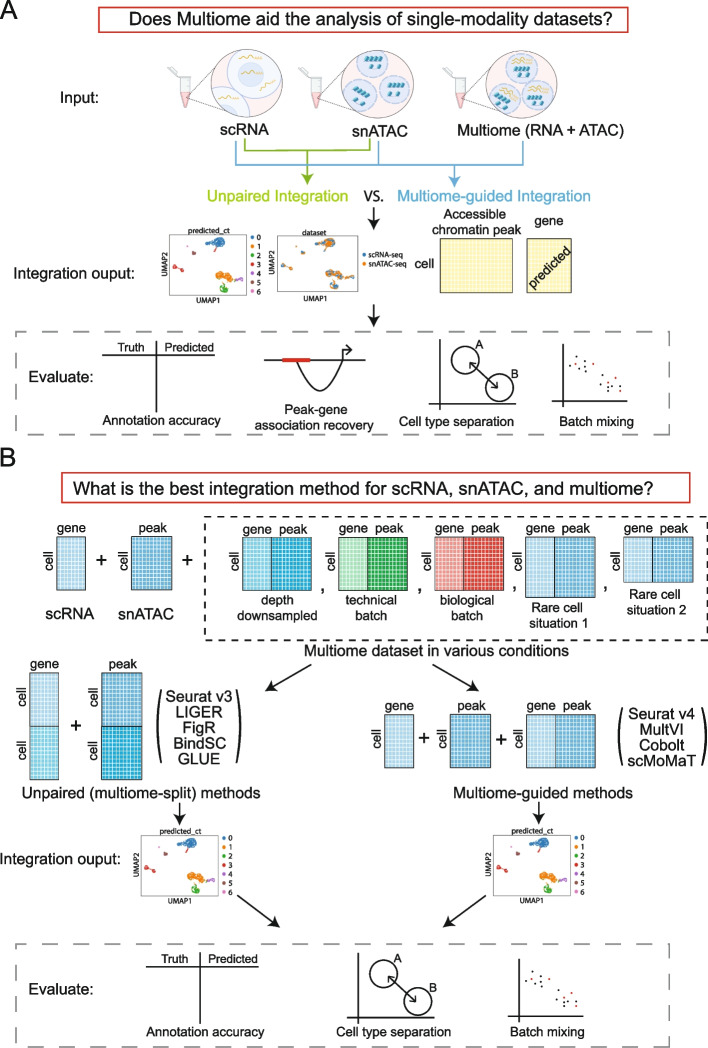
Outline of the benchmarking evaluations. **A** Scheme to evaluate if multiome data help the integration of single-modality data. **B** Scenarios simulated to evaluate multi-omic integration methods

The overall workflow of our benchmarking evaluations is summarized in Fig. 1. Figure 1A illustrates our approach to evaluate whether multiome data integration can improve the value of single-modality datasets, while Fig. 1B outlines how we assess the effectiveness of each integration method, at various conditions of the multiome dataset. To answer the proposed questions, we simulated situations where all three data types are available by using three publicly available multiome datasets [1, 13–15]. The first multiome dataset [13] profiled 10,085 peripheral blood mononuclear cells (PMBCs) and represents a simple biological system, because PBMCs can be easily divided into seven well-separated cell types (Additional file 1: Fig. S1A). The second dataset profiled bone marrow mononuclear cells (BMMC) [14, 15], an example of highly complex cell populations. BMMCs are closely related to each other transcriptionally, and contain, for example, myeloid progenitors and their closely related descendants, CD16 + and CD14 + monocytes (Additional file 1: Fig. S1B). The individual BMMC cell types are therefore much harder to separate compared to the PBMC populations, thus allowing us to thoroughly evaluate the performance of each method in both simple and complex biological systems. Moreover, the BMMC dataset is composed of samples generated from four research sites and nine donors [14, 15], which enables the analysis of batch effects and technical replicates. The third multiome dataset [1] employed SHARE-seq, a technology developed by the Buenrostro group, to profile cells from the mouse skin. This dataset offers joint profiles of 32,231 cells, which have been annotated into 22 different cell types (Additional file 1: Fig. S5). It encompasses cells from various lineages, including regenerative compartments of the hair follicles, as well as distinct cell groups like endothelial and macrophages [1]. Although the sequencing depth of this dataset is slightly lower than the two previous multiome datasets, it has the largest number of cells. Table 1 provides detailed information about each dataset.

**Table 1:** Summary of the data used for simulation. Columns are number of cells (n_cells), number of unique genes expressed per cell on average in the RNA profile (nGene_RNA), total counts expressed per cell on average in RNA profile (nCount_RNA), number of unique fragments per cell on average in the ATAC profile (nFrag_ATAC), number of peak counts per cell on average in the ATAC profile (nPeakCount_ATAC)

Source	n_cells	nGene_RNA	nCount_RNA	nFrag_ATAC	nPeakCount_ATAC
PBMC	10085	2013	4463	15510	11305
BMMC site 1 donor 2 (S1D2)	6740	1365	2525	11064	7512
BMMC site 1 or donor 1	29486	1205	2227	11798	7615
Share-seq mouse skin	32231	637	1245	5622	4231
HPAP multiome	13109	3651	13244	13462	8465
HPAP scRNA-seq	32115	4086	21032	NA	NA
HPAP snATAC-seq	26439	NA	NA	25098	16892

We evaluated five popular unpaired integration methods (Seurat v3, LIGER, FigR, BindSC, and GLUE), and four multiome-guided integration methods (Seurat v4, MultiVI, Cobolt, and scMoMaT). Given that “multiome-guided integration” methods use the multiome dataset in the integration process, the overall number of cells employed during integration and clustering is substantially increased. To account for the increased power, we created another scenario termed “unpaired (multiome-split)” in which the RNA-seq and ATAC-seq data from the multiome samples were treated as independent datasets and appended to the single-modality datasets. This category again includes the five unpaired integration methods, the only difference being that the single-modality datasets now include additional single-modality cells that were converted from the multiome cells.

To evaluate the performance of each method for cell type identification, we performed Louvain clustering [16] on the integrated embedding. For methods capable of missing modality imputation, we imputed gene expression using snATAC-seq profiles. We then evaluated the integration results in four aspects as shown in Fig. 1A. Specifically, we evaluated cell type annotation accuracy using two metrics: Adjusted Rand Index (ARI) [17] and Normalized Mutual Information (NMI) [18]. Both metrics range from 0 to 1, with 1 being the best. The detailed approach is described in the “Methods” section. The accuracy of cell type annotation depends on the number of cell clusters identified; therefore, an additional way to measure data integration quality is via the accuracy of cell type separation. Using the ground-truth annotation, we evaluated how well cells of different identities are separated, using a cell type-specific average silhouette width (ASW) [19] and a cell type Local Inverse Simpson’s Index (cLISI) [20, 21]. Furthermore, because the three data types could have technology-specific differences, we used a batch ASW [19] and the *k*-nearest neighbor batch effect test (kBET) [19] to measure batch mixing of the integrated results. These four measurements were normalized to be in the range of 0 and 1 in which 1 is the best result, namely high separation between cell types and complete mixing of data batches.

We also evaluated the quality of “peak to gene pair” predictions by assessing the accuracy of assigning an ATAC-seq peak to a specific gene. Using the measured ATAC-seq and imputed RNA-seq data, we computed the percentage of significant peak-gene pairs recovered as compared to a ground truth obtained using all cells in the multiome dataset. To penalize for the presence of false positives reported by the data integration methods, we also calculated an F1 score [18], which normalized the absolute percent recovery of the true peak-gene pairs by the occurrence of false positive and false negative relationships.

### Do Multiome data improve the annotation of single-modality datasets?

#### PBMC

**Fig. 2:**
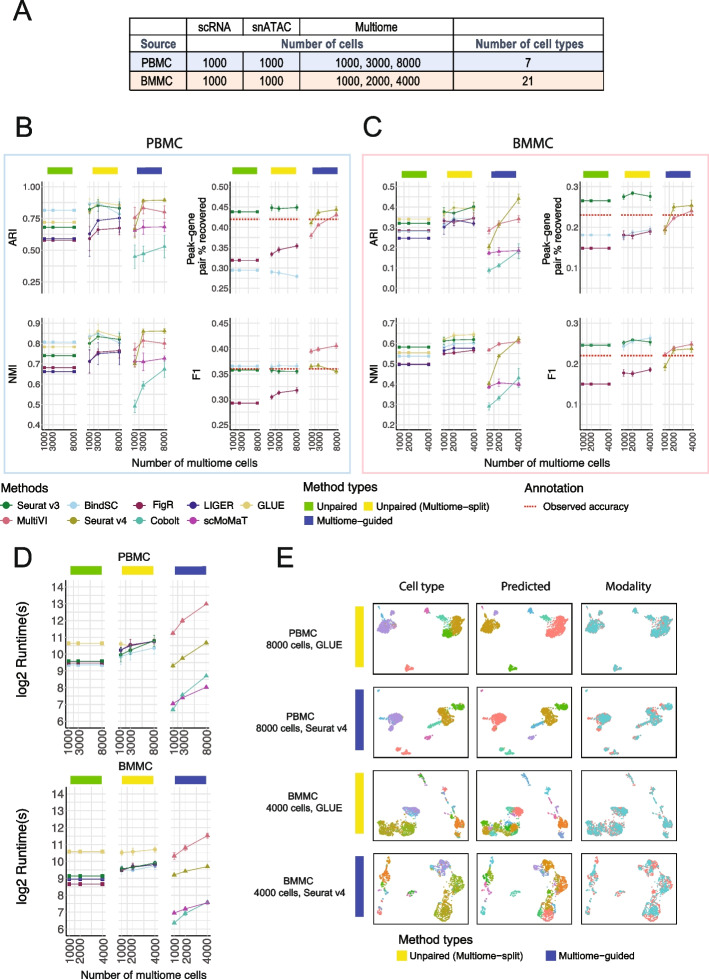
Comparison of integration performance without vs. with multiome cells. **A** The number of cells and cell types for each simulated dataset using the PBMC or BMMC multiome data as the ground truth. **B**, **C** Performance of cell type annotation and peak-gene association recovery in the PBMC-based simulations (**B**) and BMMC-based simulations (**C**). ARI and NMI measure agreement between predicted cell type and ground-truth labels. Peak-gene pair % recovered is the percentage of peak-gene pairs correctly identified compared to the ground-truth list calculated using 10,412 paired PBMC cells (**B**) and 6740 BMMC cells (**C**). F1 is the prediction accuracy normalized by the number of false positives and false negatives. The dashed line shows the percent recovery and F1 score calculated using 1000 multiome cells. Error bar is mean ± standard deviation. **D** Runtime measured in seconds, for each method, in log2 scale. Error bar is mean ± standard deviation. **E** UMAP projection using integrated embedding for a select number of methods. UMAP projection for the other methods are shown in Additional file 1: Fig. S3 (PBMC) and Additional file 1: Fig. S4 (BMMC)

To answer if multiome data improve the analysis of single-modality datasets (scRNA-seq and snATAC-seq), we first simulated the situation with 1000 scRNA-seq cells and 1000 snATAC-seq cells based on the PBMC data. These single-modality cells were integrated using each of the five unpaired integration methods. To evaluate if the inclusion of multiome data improve the analysis of single-modality datasets, we considered the situation where we have a multiome dataset, potentially with different numbers of cells (e.g., 1000, 3000, or 8000). These multiome data were integrated with the single-modality datasets using the multiome-guided methods. However, because the number of cells used during clustering and gene expression imputation impacts the clustering accuracy and peak-gene association identification, we ran the unpaired integration methods again, this time treating the multiome dataset as single-modality cells and adding them to the existing single-modality data. Here, any increase in performance is solely caused by the increase in cell number; the results from these evaluations are labeled as the “unpaired (multiome-split)” category. For each simulation, we randomly drew the cells from the 10,085 PBMC dataset and each condition was repeated five times. The parameters used for this simulation are summarized in Fig. 2A.

The evaluation results for each method are summarized in Fig. 2B. The initial cell type annotation accuracy was quite high even before the incorporation of multiome data. When integrating the unpaired data using BindSC, the accuracy was 0.81 in the Adjusted Rand Index (ARI) and 0.81 in the Normalized Mutual Information (NMI) metric (Fig. 2B). After incorporating the multiome cells for the “unpaired integration” methods as described above, all “unpaired (multiome-split)” methods show a slight increase in cell type annotation accuracy. Interestingly, incorporating 3000 multiome cells yielded similar results as incorporating 8000 cells, suggesting the existence of an empirical limit for the cell type annotation score, given this dataset.

On the other hand, when including 1000 multiome cells in the multiome-guided approaches, the results were worse compared to simply integrating the 2000 single-modality cells (Fig. 2B). This unexpected result can be attributed to the fact that 1000 multiome cells alone did not achieve sufficient cell type separation, which is a crucial requirement for the success of multiome-guided methods. However, when we used 3000 or 8000 multiome cells, Seurat v4, one of the multiome-guided methods, achieved the best cell type annotation results (Fig. 2B). Moreover, comparing the multiome-guided results with the unpaired (multiome-split) results, Seurat v4 demonstrated comparable or slightly higher performance at 3000 or 8000 cells (Fig. 2B) in ARI and NMI metrics. Thus, our findings suggest that the presence of multiome data can enhance cell type annotation in single-modality datasets, as long as a sufficient number of multiome cells are available.

Next, we evaluated the performance of each method in predicting peak-gene pairs. Peak-gene pairs are calculated using 1000 measured chromatin accessibility profiles and the corresponding 1000 imputed gene expression profiles. Here, we compared predicted peak-gene pairs to the ground-truth list calculated using multiome cells in the full PBMC data. Seurat v3 performed well at recovering the absolute number of peak-gene pairs, and the incorporation of data from multiome cells through splitting only marginally increased the performance (Fig. 2B). BindSC had a slightly better F1 score than Seurat v3, meaning that the Seurat v3 results contained more false positives (Fig. 2B). For the multiome-guided methods, the more multiome cells available during gene expression imputation resulted in higher peak-gene pair recovery (Fig. 2B). Nevertheless, the incorporation of data from multiome cells using the multiome-guided methods did not perform better than the unpaired methods, with the exception that the F1 score was higher in MultiVI (Fig. 2B).

The number of cells used for predicting peak-gene pairs influences the accuracy. To give a general idea of how well the predicted gene expression profiles are, we compared the peak-gene pair identification result to the one obtained using the real paired profiles. We included a red dashed line in Fig. 2B to indicate the percentage of peak-gene pair recovery and F1 score calculated using the measured, paired gene expression and chromatin accessibility profiles of the 1000 cells being evaluated, instead of the gene expression profile imputed from chromatin accessibility. What is surprising is that the in silico prediction profile from Seurat v3 revealed a higher percentage of recovered peak-gene pairs than the measured paired gene expression and chromatin accessibility profile from 1000 cells, although the F1 score is lower. This is likely due to the dropout issue common to single-cell assays and the predicted RNA profile can borrow information from similar cells, thus recovering the trend better. Although the predicted profiles are sometimes better than the measured gene expression profiles, here we are only imputing the expression of 1000 cells, and there are at least 1000 scRNA-seq cells used for training. Further testing is required to assess the reliability of in silico imputation when dealing with varying numbers of scRNA-seq cells and multiome cells.

#### BMMC

Having evaluated the various data integration platforms with the PBMC data, which represent a low-complexity situation with clearly defined major cell types, we next sought to determine how the different methodologies perform when analyzing data from highly complex cell populations, as is the case for bone marrow mononuclear cells (BMMC). Here, to avoid complexity caused by batch differences, we only used 6740 multiome cells from one sample (site 1 donor 2). We again started with 1000 scRNA-seq and 1000 snATAC-seq cells, and then tested the result when incorporating 1000, 2000, and 4000 multiome cells, composed of 21 cell types (Fig. 2A). In this biological system, we found that including a larger number of multiome cells improved cell type annotation, with GLUE and Seurat v3 being the top 2 performing methods in the unpaired (multiome-split) category (Fig. 2C). Among the multiome-guided methods, Seurat v4 achieved the highest ARI when the input data included 4000 multiome cells. Remarkably, when we utilized data from only 1000 or 2000 multiome cells, all multiome-guided methods exhibited poorer performance compared to splitting the multiome data into two separate, unpaired modalities (Fig. 2C), as indicated by both the ARI and NMI metrics. A similar trend was observed in the peak-gene pair prediction (Fig. 2C). The likely reason causing the poor performance of the multiome-guided methods is the limited quality of the multiome data and the high complexity of the biological system being investigated. It is worth noting that peak-gene prediction recovery and F1 score obtained via the unpaired Seurat v3 algorithm are still higher than the association calculated from the observed multiome profile, as indicated by the red dashed line in Fig. 2C.

#### Comparison of run time and visualization of integration

Another important issue to consider when comparing various computational approaches is the computation time needed to complete a given task. All methods were run with 8 CPU cores and 32 GB of RAM. Figure 2D shows the runtime, measured in log_2_ (seconds). Unpaired methods all have similar runtimes, except GLUE, which requires more compute time. Unpaired (multiome-split) category in comparison took a longer time, and this was likely due to the incorporation of the additional data from multiome experiments. Importantly, the multiome-guided methods vary greatly in runtime. Cobolt and scMoMaT were much faster than the other two methods, but unfortunately, they exhibited comparatively low clustering accuracy. Seurat v4 had comparable runtime than the unpaired (multiome-split) methods, while MultiVI took the longest runtime to complete the assigned tasks, due to its use of a variational autoencoder.

To visually examine the integration results, we generated UMAP plots using the integrated latent embedding and colored the cells by the ground-truth annotation, the predicted identity, and the dataset origin. Figure 2E summarizes the best-performing results from both the unpaired (multiome-split) and multiome-guided categories for each of the PBMC and BMMC simulations. Additional evaluation on cell type separation and batch mixing are shown in Additional file 1: Fig. S2. Most metrics show method-specific values, meaning the rankings of methods do not change across different numbers of multiome cells. Among the unpaired methods, Seurat v3 is the best at separating cell types in the integrated space, but it has the worst batch mixing result. On the other hand, FigR shows the opposite trend; it ranked the highest for batch mixing, but the lowest for cell type separation. Among the multiome-guided integration methods, MultiVI mixes the batches better while Seurat v4 often results in a higher cell type silhouette score, especially when there is a greater number of multiome cells. We also evaluated the integration results visually, through examining UMAP projections of the integration results as shown in Additional file 1: Fig. S3 for the PBMC simulations, and Additional file 1: Fig. S4 for the BMMC simulations. Visually, we do not see dramatic differences between methods, and none exhibits particularly poor cell type separation or batch mixing results.

#### SHARE-seq Mouse Skin dataset

**Fig. 3:**
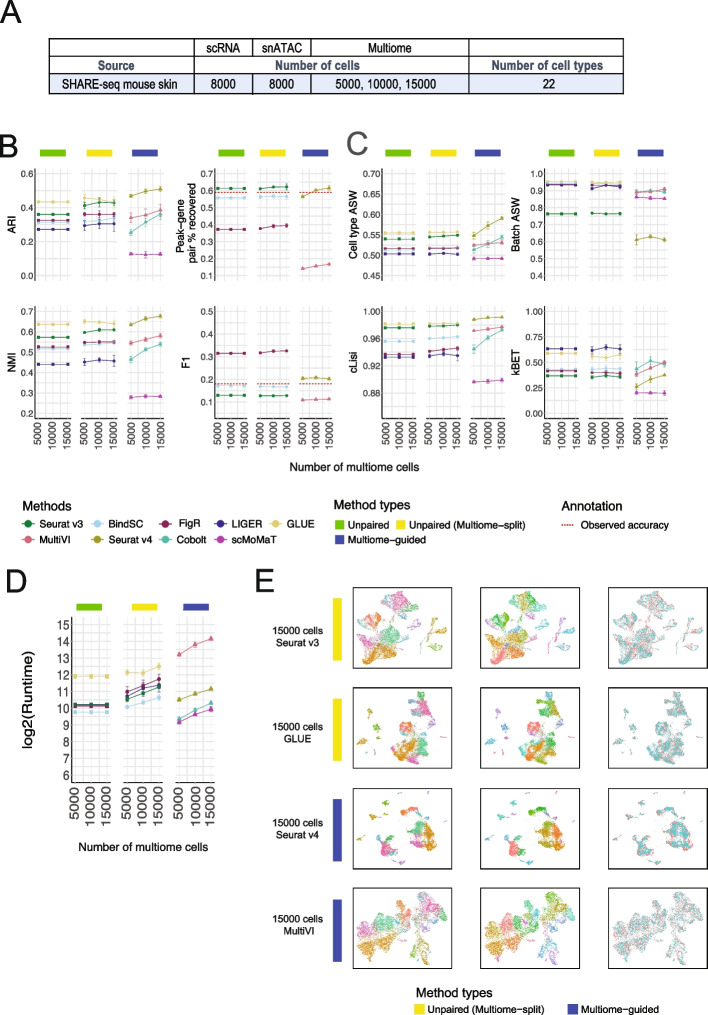
Comparison of integration performance without vs. with multiome cells, simulated using the SHARE-seq mouse skin dataset. **A** The number of cells and cell types for each simulated dataset using the SHARE-seq mouse skin data as the ground truth. **B** Performance of cell type annotation and peak-gene association recovery. ARI and NMI measure agreement between predicted cell type and ground-truth labels. Peak-gene pair % recovered is the percentage of peak-gene pairs correctly identified compared to the ground-truth list calculated using 32,231 cells from the SHARE-seq dataset. F1 is the prediction accuracy normalized by the number of false positives and false negatives. The dashed line shows the percent recovery and F1 score calculated using 8000 multiome cells. Error bar is mean ± standard deviation. **C** Performance of cell type separation and batch mixing. Cell type average silhouette width (ASW) and cell type Local Inverse Simpson’s Index (cLISI) measure separation of cell types. Batch ASW and *k*-nearest neighbor batch effect test (kBET) measure the mixing of scRNA-seq and snATAC-seq cells. Error bar is mean ± standard deviation. **D** Runtime measured in seconds, for each method, in log2 scale. Error bar is mean ± standard deviation. **E** UMAP projection using integrated embedding for a select number of methods. UMAP projection for the other methods is shown in Additional file 1: Fig. S6

We conducted a similar study using the SHARE-seq mouse skin dataset to validate our findings. Given the larger number of cells available in this dataset, we designed a simulation with 8000 scRNA-seq cells and 8000 snATAC-seq cells. We then performed integrations without or with the inclusion of 5000, 10,000, or 15,000 cells from the multiome dataset (Fig. 3A). The integration performance of each method is depicted in Fig. 3B. Consistent with the evaluation results from the PBMC- and BMMC-simulated scenarios, GLUE and Seurat v3 emerged as top-performing methods in the “unpaired (multiome-split)” category, with GLUE being the best performer. Among the “multiome-guided” methods, Seurat v4 exhibited the best performance and outperformed all methods in the two “unpaired integration” categories.

Regarding peak-gene pair recovery, Seurat v3 demonstrated the highest recovery in terms of the absolute number of peak-gene pairs, surpassing the value obtained from the truly paired profiles. However, when considering the F1 score, which corrects for false positive and false negative rates, FigR performed significantly better than the other methods and even outperformed the F1 score from the truly paired profiles. Seurat v4 achieved the highest F1 score among the multiome-guided methods and ranked second in overall performance, just behind FigR. In terms of runtime, GLUE had the longest execution time among the “unpaired integration” category, while the remaining methods had similar runtime overall, comparable to that of Seurat v4. MultiVI had the longest runtime, whereas Cobolt and scMoMaT were the fastest (Fig. 3C). Representative UMAP plots after integration are displayed in Fig. 3D. UMAP plots of other integration methods are shown in Additional file 1: Fig. S6.

In conclusion, our findings confirm that the incorporation of multiome cells enhances cell type annotation when there are sufficient cells to resolve the cell type heterogeneity within the multiome dataset. Regarding the recovery of peak-gene pairs, Seurat v4 performed slightly better than the paired cells consistently when there were a large number of multiome cells, but there was always another method from the “unpaired (multiome-split)”category that performed better. However, this top-performing method varied depending on dataset and metric type.

### How to spend your sequencing dollars: more cells or increased sequencing depth?

**Fig. 4:**
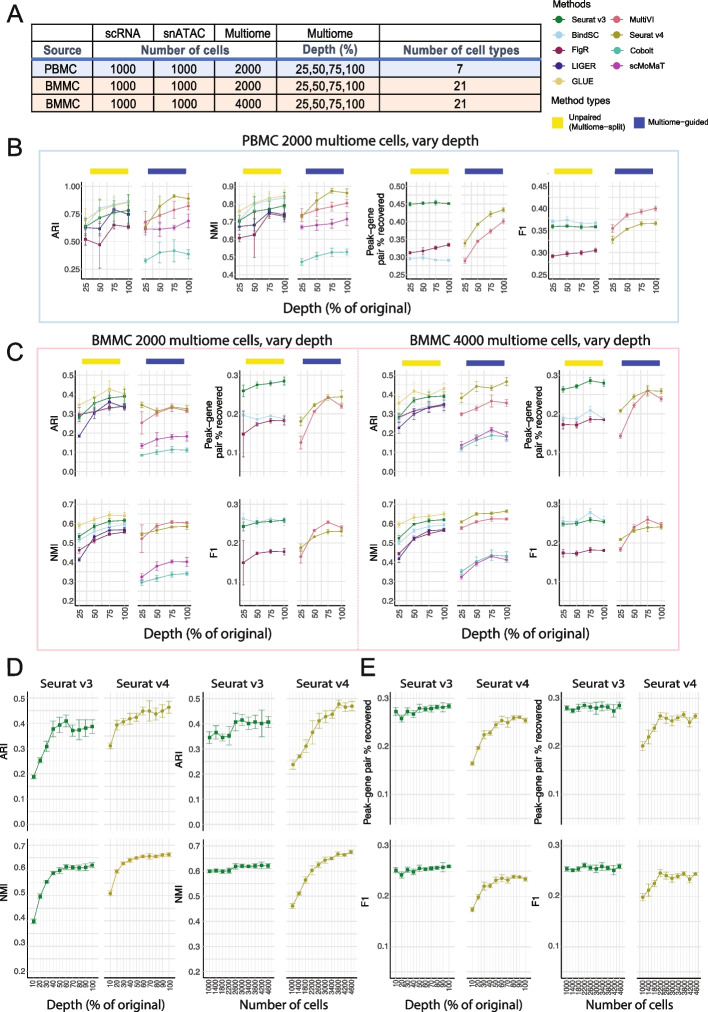
Evaluation of integration performance at varying sequencing depth for multiome cells. **A** Details of the simulation scheme. **B**, **C** Performance of cell type annotation and peak-gene association recovery in the PBMC-based simulations (**B**) and BMMC-based simulations (**C**: left panel, 2000 multiome cells; right panel, 4000 multiome cells). ARI and NMI measures agreement between predicted cell type and ground-truth labels. Peak-gene pair % recovered is the percentage of peak-gene pairs correctly identified comparing to the ground-truth list calculated using 10,412 paired PBMC cells (**B**) and 6740 BMMC cells (**C**). F1 is the prediction accuracy normalized by the number of false positives and false negatives. **D** Performance of cell type annotation using Seurat v3 or Seurat v4 at increasing depth or increasing number of cells. **E** Performance of peak-gene association recovery using Seurat v3 or Seurat v4 at increasing depth or increasing number of cells. For all subplots, error bar is mean ± standard deviation

Experimentalists are commonly constrained by budget limitations and need to consider whether sequencing a larger number of cells at low depth or a smaller number of cells at high depth is the more productive approach. To answer this question, we evaluated how the sequencing depth of the multiome dataset influences the integration result. Since we know that including multiome data improves cell type annotation for the single-modality datasets, for this analysis, we evaluated the cell type annotation accuracy of the three data types together. Table 1 shows the sequencing depth of the original multiome samples. To simulate data with lower depths, we downsampled the reads for both RNA and ATAC profiles to 25, 50, and 75% of the original data (Fig. 4A) and compared these results to the original samples. We performed this experiment on both the PBMC dataset (Fig. 4B) and the BMMC dataset (Fig. 4C). We did not perform this experiment on the SHARE-seq dataset because the original sequencing depth was not sufficient. For the PBMC study, an increase in sequencing depths resulted in an increase in cell type annotation accuracy for all methods, with Seurat v4 achieving the highest ARI and NMI scores among all methods for 75 and 100% sequencing depth (Fig. 4B). In contrast, when we used the BMMC data set as the input, we noted that when including only 2000 multiome cells, regardless of sequencing depth, the unpaired method, GLUE, performed the best (Fig. 4C left). However, when we included 4000 cells in the BMMC multiome sample, 50% of read depth was sufficient for Seurat v4 to annotate the cell types most accurately (Fig. 4C right). These conflicting results prompted us to ask whether sequencing depth is less important than cell number.

To answer this question, we designed another simulation using the BMMC dataset. Given a fixed cost for 1,000,000 RNA-seq reads and 4,000,000 ATAC-seq reads, we used either 400 cells with 100% of the depth (see Table 1), or 10% of the reads for 4000 cells. Next, we analyzed the datasets using Seurat v3 and Seurat v4. We chose to use Seurat v3 instead of GLUE because Seurat v3 allows peak-gene pair recovery and is just slightly worse than GLUE in the unpaired (multiome-split) category. For cell type annotation accuracy, the sequencing depth curve plateaued sooner than the number of cells curve (Fig. 4D). For Seurat v4, the ARI and NMI scores did not increase much beyond 60% sequencing depth, while both scores increased consistently as the number of cells increased (Fig. 4D). Comparing Seurat v3 with Seurat v4, we noted that Seurat v4 performed better when there was 30% sequencing depth given 4000 cells or 2600 cells given 100% depth (Fig. 4D). Therefore, for the accuracy of cell type annotation for integrated data, having more cells is more important than having a higher sequencing depth. Importantly, once a sufficient number of cells have been profiled to capture the complexity of a given sample, the multiome-guided methods, specifically Seurat v4, perform the best. Our analysis also demonstrated that the “sufficient” number of cells depends on the complexity of the biological system in question. For PBMC, we see that if the goal is to detect seven distinct cell types, 2000 cells is already sufficient. However, for BMMC with its more complex cell type composition, at least 2600 cells are needed to achieve adequate cell type annotation accuracy.

In addition to the cell type annotation accuracy, we also evaluated the recovery of peak-gene association for the 1000 single-modality ATAC-seq cells when incorporating multiome samples generated at ten different depths and numbers of cells. We noted that Seurat v3 performed consistently better than Seurat v4 (Fig. 4E). Moreover, the number of cells and sequencing depth did not affect the percentage of peak-gene pair recovery nor the F1 score. This is likely because Seurat v3 predicts RNA expression using the nearest neighbor approach on the integrated space, and the software was given a sufficient number of cells in the scRNA-seq dataset for the prediction, thus changes in the multiome data did not affect the result.

Next, we evaluated cell type separation and batch mixing results as summarized in Additional file 1: Fig. S7. Most metrics increased slightly as sequencing depth increased, but the ranking of methods is similar as described before. Overall, Seurat v4 exhibited the best separation of cell types in the integrated space, but the mixing of batches is the worst, across sequencing depths. A UMAP projection of each method under each simulated scenario is shown in Additional file 1: Fig. S8-10 for visual comparison.

Overall, we conclude that the number of cells in the multiome data is more critical than sequencing depth for annotating cell types in the integrated data. On the other hand, increasing depth or number of cells of the multiome dataset does not improve the peak-gene pair recovery result for the “unpaired (multiome-split)” methods, while higher depth or larger number of multiome cells both improve the peak-gene pair recovery for the “multiome-guided” methods slightly.

### Which method is the best at removing batch effects?

**Fig. 5:**
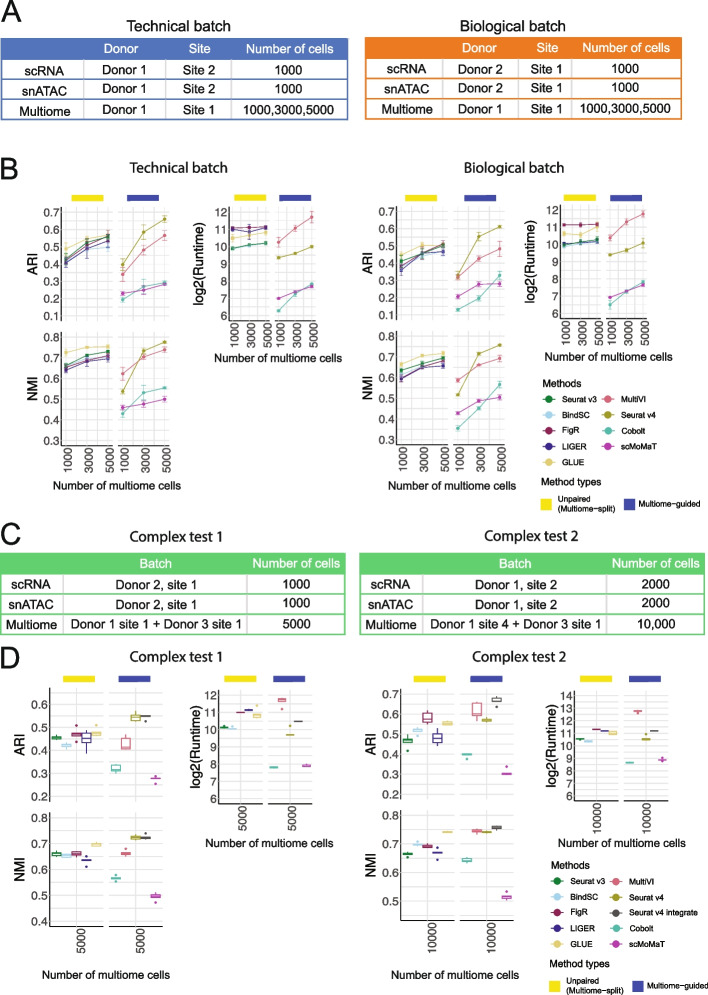
Evaluation of integration performance in the presence of batch effects. **A** Simulation details for the constructed data with technical batches and biological batches. **B** Performance of cell type annotation and runtime in the presence of technical and biological batches shown in **A**. ARI and NMI measure agreement between predicted cell type and ground-truth labels. Runtime is measured in seconds, for each method, in log2 scale. Error bar is mean ± standard deviation. **C** Simulation details for two datasets with more complex batch structures. **D** Performance of cell type annotation and runtime in the presence of technical and biological batches shown in **C**. ARI and NMI measure agreement between predicted cell type and ground-truth labels. Runtime is measured in seconds, for each method, in log2 scale. Whisker is 1.5 times the inter-quartile range

It is common that scRNA-seq and snATAC-seq data are generated by different labs or from different individuals than the multiome data. Therefore, another key characteristic for integration methods is whether they can integrate samples displaying batch effects. To answer this question, we leveraged the complex batch structure present in the BMMC dataset. Figure 5A shows the technical batch or biological batch structure we aimed to evaluate, with the multiome cells coming from a different research site, or a different donor. Figure 5B shows results of cell type annotation accuracy for the “unpaired (multiome-split)” methods and the “multiome-guided” methods. We again saw increasing cell type annotation accuracy as the number of multiome cells increased. With 3000 or more multiome cells, Seurat v4 again was the best-performing method, although GLUE showed comparable NMI scores. Seurat v4 is a supervised approach, meaning that the multiome sample serves as a reference to which the single-modality datasets are mapped to. Figure 5B shows that although the multiome sample has strong batch effects (Additional file 1: Fig. S11), the supervised mapping approach resulted in the most accurate cell type annotation. Additional integration results are shown in Additional file 1: Fig. S12 and the UMAP projections are shown in Additional file 1: Fig. S13-14.

To further challenge all methods in the situation of complex mixtures of samples, we considered two situations where the multiome sample includes cells from a mixture of two donors, and the scRNA-seq and snATAC-seq data come from the same (Fig. 5C left) or different research sites (Fig. 5C right). Due to batch effects in the multiome samples, we added one more category called “Seurat v4 integrate,” in which the integration of samples was first done on each modality separately, then the two modalities were joined using the Seurat v4 weighted nearest neighbor approach, and lastly combined with the single-modality dataset (see more in Additional file 1: Supplementary methods). Figure 5D (left) shows that in the case of low batch effects between the two donors, Seurat v4 and “Seurat v4 integrate” performed similarly well at annotating cell types. However, in the presence of stronger batch effects, “Seurat v4 integrate” outperformed all other methods for cell type annotation (Fig. 5D right), with much higher cell type separation as measured in cell type average silhouette width (ASW) (Additional file 1: Fig. S15). In these two tests, “unpaired (multiome-split)” methods achieved similar performance, although GLUE was consistently ranked among the top two in ARI and NMI. In comparison, “Seurat v4 integrate” was consistently the best, although just slightly better than others. From the UMAP projection in Additional file 1: Fig. S16, we noted that “Seurat v4 integrate” mixes cells from the two multiome samples much better than Seurat v4, especially for “Complex test 2.” Therefore, when the multiome data include two donors with strong batch effects, integration across the batches is required before mapping the single-modality datasets.

#### Integration of single-cell multi-omic datasets from Human Pancreas Analysis Program (HPAP)

Simulated datasets are critical for evaluating integration performance due to the availability of ground-truth cell type annotations. However, there are limitations with the use of simulated datasets. The three data types we simulated all came from the same single-cell multiome dataset and thus represent the most idealized situation where there is no batch effect between the single-modality datasets and the multiome dataset. In reality, there are significant differences between datasets generated from different technologies, due to the different library preparation workflow. Moreover, the difference between scRNA-seq and snRNA-seq is non-negligible.

**Fig. 6:**
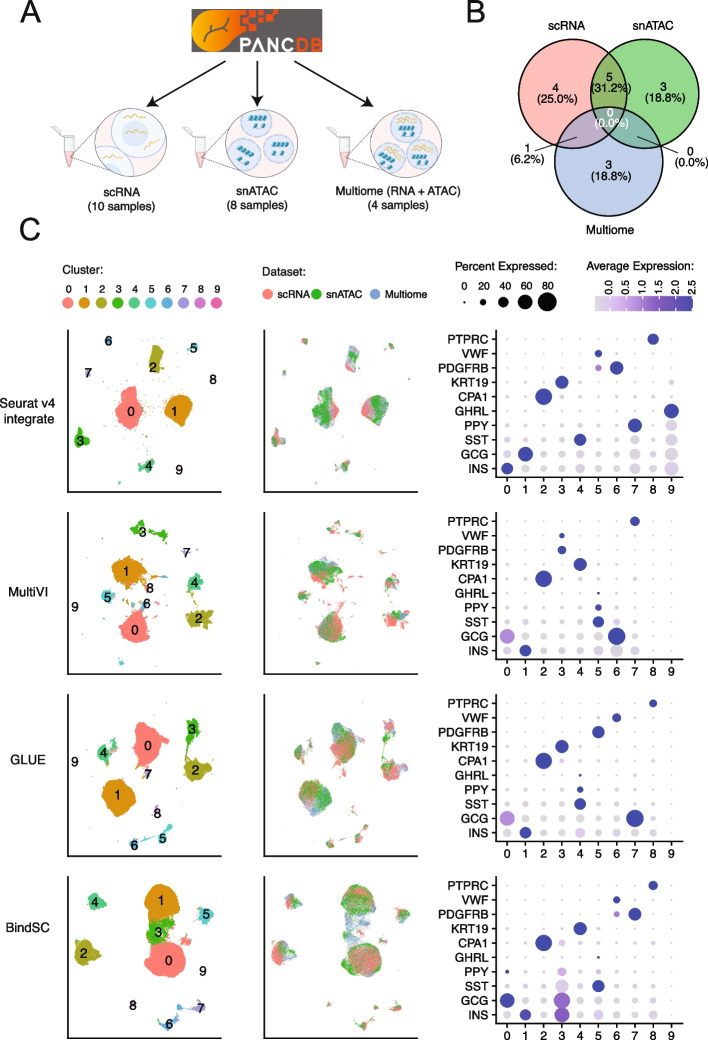
Integration of scRNA-seq, snATAC-seq, and multiome datasets from the Human Pancreas Analysis Program (HPAP). **A** Illustration of the number of samples for each data type from PANC-DB, the website releasing all HPAP datasets. **B** Overlap of samples from the same human donor between data types. **C** Integration results: UMAP projection using integrated embedding for a select number of methods, colored by cluster ID (left), data type (middle), and dot-plots showing the expression of marker genes per cluster, colored by average expression, sized by the percentage of cells expressing the gene (right). Integration results of the rest of the methods and other evaluation metrics are shown in Additional file 1: Fig. S17

To evaluate the performance of different integration methods in a more realistic situation, we used data generated by the Human Pancreas Analysis Program (HPAP; https://hpap.pmacs.upenn.edu/about-pancdb; PMID: 36206763; PMID: 31127054). Specifically, we integrated 22 samples generated from human islets, including 10 scRNA-seq samples, 8 snATAC-seq samples, and 4 multiome samples (Fig. 6A). The scRNA-seq and snATAC-seq datasets were obtained from healthy adult human samples, while the multiome dataset included samples from one healthy adult, one young healthy donor, and two type 2 diabetic donors. Some of the datasets were obtained from islet cells in the same donor; Fig. 6B shows the overlap of donors between datasets. We believe the HPAP data represent a realistic scenario where all three data types are present, and the goal is to integrate all cells to achieve one uniform cell type clustering result. We integrated the datasets using methods belonging to the “unpaired (multiome-split)” and “multiome-guided integration” categories. However, due to the presence of strong batch effects, we tried our best to modify each method’s default pipeline to limit the impact of technical batch differences. For methods that require *z*-score standardization, we performed this donor-by-donor and aggregated the scaled data. In situations where highly variable gene selection is needed, we ran highly variable gene selection per donor and aggregated the selected genes across donors by ranking to obtain the most representative genes. More details on specific optimization for each method are in the Additional file 1: Supplementary methods.

Results of each integration method are shown in Fig. 6C and Additional file 1: Fig. S17A. Since we did not have ground-truth annotation, we analyzed the integration results in three different ways: UMAP projections using the integrated embedding labeled by cluster id (left column) and dataset origin (middle column), and a dot plot showing the average gene expression and percentage of expressed cells for 10 marker genes, one per expected cell type (right column). Overall, Seurat v4, MultiVI, and GLUE all showed good separation of the major cell types (Fig. 6C). However, Seurat v4 revealed the cleanest result as it produced 10 clusters, each expressing only one cell type marker gene and showing the least amount of co-expression of marker genes for different cell types. The two rare cell populations in the human islet, *PPY*-expressing gamma cells and *GHRL*-expressing epsilon cells, were also found by Seurat v4. For MultiVI and GLUE, although the broad cell type separation was decent, the *GCG*-expressing cell population was separated into two clusters, possibly due to technical reasons. Moreover, both methods failed to identify distinct clusters for gamma cells or epsilon cells. The results from other integration methods are shown in Additional file 1: Fig. S17A. We also calculated the same metrics measuring the mixing of batches (scRNA-seq, snATAC-seq, or multiome), and the mixing of cells from different donors (Additional file 1: Fig. S17B). Like previous trends, Seurat v4 showed relatively poorer mixing of cells from different types of assays and donors, while FigR showed better batch mixing than the other methods. This HPAP analysis also allowed us to better evaluate each method’s run time, as there were more than 70,000 cells in this dataset. We again found MultiVI to be the slowest method. Among the “unpaired (multiome-split)” methods, LIGER took the longest while the remaining methods took similar time, also comparable to Seurat v4. ScMoMaT and Cobolt were the fastest, but again, their integration performance was less optimal.

### When your single-modality datasets have different cell types as the Multiome dataset, do the integration methods still work?

**Fig. 7:**
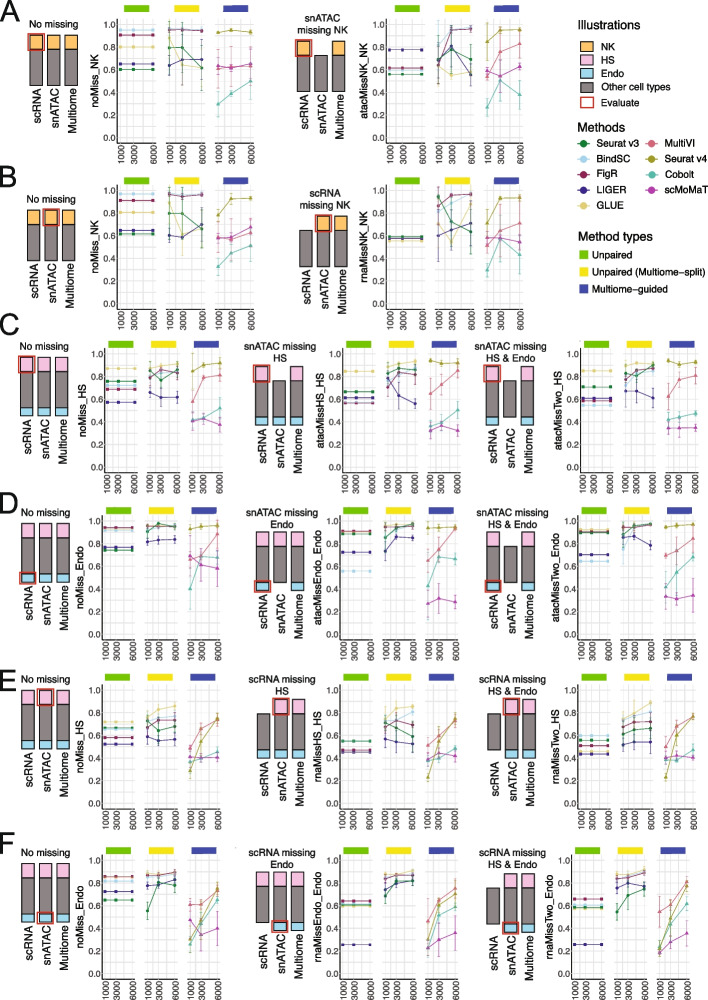
Evaluation of rare cell recovery when one of the single-modality datasets miss the rare population of cells. **A**, **B** F1 score of recovering “Natural Killer” (NK) cells in PBMC-simulated situations. **A** F1 score of recovering NK cells in scRNA-seq at the baseline situation (left) and when snATAC-seq does not have NK cells (right). **B** F1 score of recovering NK cells in snATAC-seq at the baseline situation (left) and when scRNA-seq does not have NK cells (right). **C**–**F** F1 scores of recovering “Hair Shaft” (HS) cells or “Endothelial” (Endo) cells in SHARE-seq-simulated situations. **C** F1 score of recovering HS cells in scRNA-seq at the baseline situation (left), versus when snATAC-seq does not have HS cells (middle), versus when snATAC-seq does not have HS and Endo cells (right). **D** F1 score of recovering Endo cells in scRNA-seq at the baseline situation (left), versus when snATAC-seq does not have Endo cells (middle), versus when snATAC-seq does not have HS and Endo cells (right). **E** F1 score of recovering HS cells in snATAC-seq at the baseline situation (left), versus when scRNA-seq does not have HS cells (middle), versus when scRNA-seq does not have HS and Endo cells (right). **F** F1 score of recovering Endo cells in snATAC-seq at the baseline situation (left), versus when scRNA-seq does not have Endo cells (middle), versus whens cRNA-seq does not have HS and Endo cells (right). For all subplots, error bar is mean ± standard deviation

**Fig. 8:**
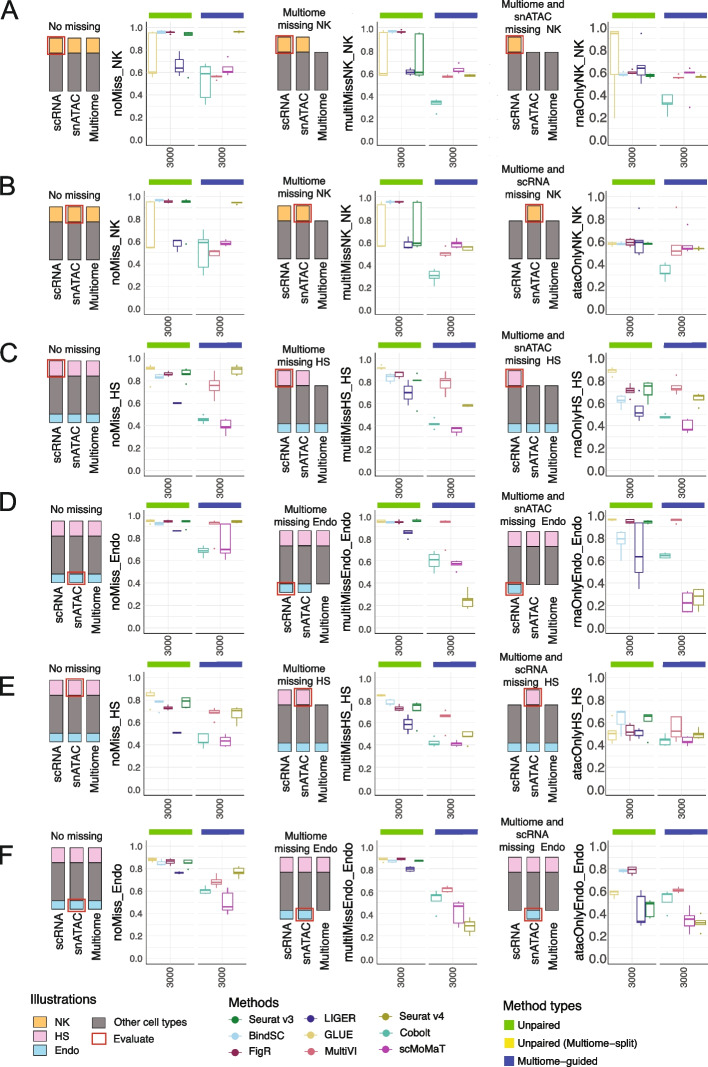
Evaluation of rare cell recovery when the multiome dataset or two of the three datasets to be integrated miss the rare population of cells. **A**, **B** F1 score of recovering “Natural Killer” (NK) cells in PBMC-simulated situations. **A** F1 score of recovering NK cells in scRNA-seq at the baseline situation (left), versus when multiome does not have NK cells (middle), versus when multiome and snATAC-seq do not have NK cells (right). **B** F1 score of recovering NK cells in snATAC-seq at the baseline situation (left), versus when multiome does not have NK cells (middle), versus when multiome and scRNA-seq do not have NK cells (right). **C**–**F** F1 scores of recovering “Hair Shaft” (HS) cells or “Endothelial” (Endo) cells in SHARE-seq-simulated situations. **C**, **E** F1 score for recovering HS (**C**) and Endo (**E**) in scRNA-seq at the baseline situation (left), versus when multiome does not have the cell type (middle), versus when multiome and snATAC-seq do not have the target cell type (right). **D**, **F** F1 score for recovering HS (**E**) and Endo (**F**) in snATAC-seq at the baseline situation (left), versus when multiome does not have the cell type (middle), versus when multiome and scRNA-seq do not have the target cell type (right). For all box plots, the whisker is 1.5 times the inter-quartile range

It is not uncommon to have unique cell types in a certain dataset. This could happen when one of the datasets is generated in a different lab that may have slightly different tissue digestion or single-cell/nucleus isolation protocol. Moreover, there are intrinsic distinctions between scRNA-seq datasets and the gene expression profiling in the multiome dataset, as the former is single cell, with mRNA mostly from the cytoplasm, while the latter is single nucleus, with the mRNA coming from the nucleus. In these situations where the scRNA-seq, snATAC-seq, and multiome have cell types that only exist in two or even one dataset, it is of interest to investigate if the integration algorithms can accurately identify the unique cell populations that are not shared by all three datasets. To this end, we considered two scenarios: (1) scRNA-seq or snATAC-seq do not share the same cell types, e.g., NK cells are found in scRNA-seq and multiome, but not snATAC-seq dataset; (2) multiome dataset does not share the same cell populations as the single-modality datasets, e.g., NK is missing in the multiome dataset, but present scRNA-seq and snATAC. For each situation, we explored varying degrees of cell type overlap, illustrated in Figs. 7 and 8.

We performed simulations for the above-described scenarios using the PBMC and the SHARE-seq data as the source. The BMMC dataset was not used for this simulation because there were not enough numbers of cells in any single donor, making it challenging to evaluate the impact of rare cells, and the batch effects become strong if all donors are combined. For the PBMC and SHARE-seq simulations, we ensured that the targeted cell types being excluded in certain datasets had at least 50 cells during the clustering step. In the PBMC simulation, the NK cells account for 4.6% of the 10,085 total cells. We considered the situation where there are 1000 scRNA-seq cells, 1000 snATAC-seq cells, and 1000, 3000, or 6000 multiome cells. We calculated the F1 score for recovering the NK cells in the scRNA-seq dataset and that in the snATAC-seq dataset separately. By comparing the two plots in Fig. 7A, we found that the absence of NK cells in the snATAC-seq data (right) led to decrease in the correct identification of NK cells in the scRNA-seq data, as compared to the baseline (left) using the “unpaired integration” methods. If we incorporate the multiome dataset, which contained the NK cells, the F1 score was comparable to the baseline, for both the “unpaired (multiome-split)” and the “multiome-guided integration” methods. Figure 7B shows the F1 score of identifying NK cells in snATAC-seq data when scRNA-seq data does not have NK cells. Similar to Fig. 7A, the absence of NK cells from scRNA-seq data led to a decrease in recovering NK cells in snATAC-seq data after integration.

We generalized this evaluation strategy to the SHARE-seq dataset where we filtered out cell types with less than 500 cells and subset the dataset to one with a total of 10,000 cells, in which “Hair Shaft” (HS) cells account for 10% and “endothelial” cells account for 5% of the total cell population. Using this dataset, we evaluated the situation where snATAC-seq dataset does not have HS cells, endothelial cells, or both and calculated the F1 score for identifying the rare cell population in scRNA-seq (Fig. 7C,D). We repeated a similar setup but with the rare cell population missing in the scRNA-seq data and evaluated the recovery of the rare cell population in snATAC-seq (Fig. 7E,F). We also considered the situation when each single-modality dataset has one cell type (scRNA-seq has HS, snATAC-seq has Endo), but the result (data not shown) is similar to the single cell type missing scenarios shown in Fig. 7.

None of the unpaired integration methods were consistently better than the others when a cell type was missing from one of the single-modality datasets. We observed that, in general, if one single-modality dataset lacks a certain cell type, the identification of this cell type after integration is harder, with the exception of the case shown in Fig. 7D. In this case, the baseline performance for some of the unpaired integration methods is comparable to the “snATAC missing Endo” or “snATAC missing HS & Endo” results. One hypothesis is that endothelial cells have very distinct gene expression patterns compared to other cell populations. Therefore, they are distinct enough to be identified as one cluster in the integrated space. “Unpaired (multiome-split)” integration methods and “multiome-guided” integration methods generally achieved a similar accuracy. The top-performing method in the “unpaired (multiome-split)” category was GLUE for the SHARE-seq simulated scenarios, but BindSC and FigR performed better for the PBMC-simulated cases. Seurat v4 is the best-performing method most of the times, except for situations shown in Fig. 7E,F.

For the second group of scenarios, we again simulated data using the PBMC and SHARE-seq datasets. Here, our goal was to test the missing cell type situation where multiome did not contain a specific cell population, or if two of the three datasets missed the cell type. Figure 8A,B shows the integration results for scenarios simulated using the PBMC dataset, and the target cell type is NK cells. Comparing the baseline (left) to the “Multiome missing NK” (middle) result, we observe a large decline in the F1 score for Seurat v4 and Cobolt. The other multiome-guided methods are less affected. Results from the unpaired integration methods are included as a baseline, demonstrating results achieved by the integration of the scRNA-seq and snATAC-seq datasets. The rightmost panel shows an extreme situation where the cell type is only present in scRNA-seq. In this scenario, we observe a decline in performance for the unpaired integration methods as well. We repeated the analysis using the SHARE-seq dataset and simulated with two rare cell populations again, checking if the result would differ depending on the number of cells in the two cell types (Fig. 8C–F). Similar to what we observed in the PBMC-based simulations, Seurat v4 was affected the most. This is expected because Seurat v4 is a supervised integration approach; it builds a reference dataset using the multiome cells and then maps the single-modality dataset onto the reference space. Our results suggest that, if there are single-modality-specific cell types, they are unlikely to be identified as a unique cell population using the Seurat v4 integration. In this situation, GLUE or other unpaired integration methods might better preserve those rare cell populations.

## Discussion

In summary, we evaluated nine multi-omic integration methods under five simulated scenarios and one real data integration problem. Firstly, we showed that the incorporation of multiome data improves the cell type annotation accuracy of scRNA-seq and snATAC-seq data when there are a sufficient number of cells in the multiome data to reveal cell type identities. Secondly, we showed that the number of cells in the multiome data plays a more important role than sequencing depth per cell for cell type annotation accuracy. Thus, when generating a multiome dataset with a fixed budget, a better strategy is to profile more cells so that rare cell types can be captured. Thirdly, when the three datasets to be integrated are confounded by batch effects, Seurat v4 resulted in the best cell type annotation accuracy, for both simulated and real data scenarios. Forthly, we explored the integration performance in situations where there is an incomplete overlap of cell types between the three data types. Lastly, we tested each method on a real data situation using data from the HPAP consortium. 

In all evaluations, GLUE and Seurat v4 both demonstrated superior performance at resolving cell type heterogeneity. In situations where there are many multiome cells, Seurat v4 performed better than GLUE. On the other hand, when the multiome data have an insufficient number of cells to reveal accurate cell types, the Seurat v4 integration resulted in poor annotation accuracy and GLUE was the better option. Seurat v4 is a supervised approach, so it is expected that the number of multiome cells affect the integration performance greatly. However, for the other multiome-guided methods, e.g., MultiVI, Cobolt, and scMoMaT, the hope is that the single-modality cells can help the clustering when multiome cells are small. However, as shown in Figs. 2 and 3, MultiVI, Cobolt, and scMoMaT performed worse than the “unpaired (multiome-splitted)” integration methods that do not leverage the paired relationship of the multiome data. Therefore, when the multiome dataset has a small number of cells, it is better to treat the multiome cells as unpaired and append them to the single-modality datasets for the integration of three datasets.

There are several limitations of this study. Firstly, our simulations represent the most ideal situation, where the single-modality cells are generated from the exact same dataset as the multiome cells. In reality, the single-modality and the multiome data are generated from different experimental kits that could have slight differences since the multiome workflow is optimized to capture both gene expression and chromatin accessibility. Moreover, the gene expression captured through the multiome workflow is, in fact, measuring mRNA in individual nuclei, while scRNA-seq captures mRNA in whole cells. Slight differences between snRNA-seq and scRNA-seq datasets have been reported [22]. We tried to overcome these limitations by including the HPAP dataset as a demonstration of integration performances in a real-life case, and the major findings are consistent. Lastly, the PBMC dataset did not have expert-annotated cell type labels. We followed a tutorial by Seurat v4 to obtain annotations [23], thus the evaluation of PBMC-simulated scenarios might favor Seurat v4. However, the BMMC and SHARE-seq data were manually annotated by experts and Seurat v4 still showed outstanding performance in evaluations based on these datasets.

Secondly, we did not explore the possibility of imputing chromatin accessibility from scRNA-seq or appending imputed profile with observed multiome sample. To truly integrate the three data types and understand the underlying *cis*-regulatory logic, one would hope to impute the missing modality for both the scRNA-seq and snATAC-seq data, and then append the imputed profiles with the multiome dataset to identify peak-gene pairs with the largest number of cells. Therefore, additional work needs to be done to evaluate the performance of different methods in jointly integrating the imputed single-modality datasets with the multiome samples for downstream analyses.

## Conclusions

Our benchmarking evaluations showed that multiome data are helpful for annotating single-modality data. The number of cells in the multiome data is critical to ensure a good cell type annotation after integration and the exact number of cells depends on the complexity of the biological system. When generating a multiome dataset, the number of cells is more important than sequencing depth for cell type annotation. Lastly, Seurat v4 is the best at integrating scRNA-seq, snATAC-seq, and multiome data even in the presence of complex batch effects.

## Methods

### Datasets

#### Peripheral blood mononuclear cell (PBMC) dataset

This dataset was generated using the 10 × Genomics Single Cell Multiome ATAC + Gene Expression kit [13]. The PBMC dataset with granulocytes removed was downloaded from the 10 × Genomics website, which included 11,909 cells. The dataset was processed and annotated into 30 cell types following the Seurat tutorial [8, 23]. We grouped similar cell types and refined the annotations into 9 broad cell types (similar to the level 1 categories from the Azimuth database [3]): B-cells (“B”), CD4 T cells (“CD4 T”), CD8 Naïve T cells (“CD8 Naïve”), CD8 Effector T cells (“CD8 TEM”), Dendritic cells (“DC”), Monocytes (“Mono”), Nature killer cell (“NK”), other T cell (“other_T”), and other cell categories (“other”). The ATAC-seq profile released on 10 × Genomics website was counting the Tn5 insertion events in each genomic region. Here, we retabulated the cell-peak matrix by the number of reads overlapping each genomic region, using the Signac’s FeatureMatrix function [24]. We used the peak-based counting result as input for the peak-gene pair identification (described below) and subsequent simulations. The list of peak-gene pairs identified using all cells in the multiome dataset (10,412 cells) is treated as the ground truth when calculating percentage of peak-gene pair recovery or F1 score. “Other_T” and “other” cells were excluded from the data simulation due to their extensive separation in the UMAP embedding. After removal of cells, there are 10,085 cells used for simulation.

#### Bone marrow mononuclear cells (BMMC) dataset

This dataset was generated as part of the “Open Problems in Single-cell Analysis” competition [25]. BMMC cells from nine healthy donors were profiled at four different research sites using the 10 × Multiome ATAC + Gene Expression kit. The dataset was analyzed by Lance and colleagues [25], who annotated the cells into 22 cell types. The values in the cell-peak matrix of the ATAC-seq data was also the insertion-based counting, so we again converted it into peak-based counting as mentioned above. Data simulations related to Figs. 2 and 4 were performed using cells from the site 1 donor 2 (S1D2) BMMC sample. This sample contains 6740 cells, annotated into 21 cell types. The peak-gene pair prediction accuracies shown in Figs. 2 and 4 were calculated by comparing the result to a ground-truth list generated with the S1D2 sample. To simulate technical batch and biological batch effects (Fig. 5), we used cells generated at research site 1 or from donor 1, which includes a total of 29,486 cells, composed of 21 cell types (Additional file 1: Fig. S1B).

#### SHARE-seq mouse skin dataset

This dataset was generated by Ma et al. [1], who profiled cells from the mouse skin using a multi-omic profiling technique called simultaneous high-throughput ATAC and RNA expression with sequencing (SHARE-seq). We downloaded the RNA-seq and ATAC-seq data from GEO: GSM4156608 and GEO: GSM4156597, respectively. A total of 34,774 cells with both RNA-seq and ATAC-seq profiles are available. Moreover, a ground-truth annotation was provided by the authors as part of the GEO: GSM4156597 data. According to Ma et al. [1], scRNA-seq was normalized with the standard Seurat v3 pipeline, which was first library-size normalized and then log1p transformed. Clustering was done separately for the RNA-seq and ATAC-seq data. Cell types were annotated for the RNA-seq portion by examining marker gene expression while the activity of the lineage-determining transcription factor was inferred from the ATAC-seq portion and used for cell type annotation. The authors compared the RNA-seq and ATAC-seq clustering results and derived one final cell type annotation, which was treated as ground truth in our analyses. We removed cells labeled as “Mixed,” resulting in a total of 32,231 cells from 22 cell types. Furthermore, we removed RNA features expressed in less than 3 cells, and ATAC regions with 0 counts.

#### Human Pancreas Analysis Program (HPAP)

HPAP is a NIDDK-funded initiative that aims to perform deep profiling of human endocrine pancreas and to make the data highly accessible to the broader community of diabetes researchers [26]. We employed the scRNA-seq and snATAC-seq available on PANC-DB (https://hpap.pmacs.upenn.edu), the central space releasing all data generated from the HPAP consortium. We browsed PANC-DB and selected pancreatic islet samples from healthy donors with age ≥ 18. A scRNA-seq sample was included if it had a median number of counts ≥ 2000 and the number of cells was between 2000 and 5000. A snATAC-seq sample was included if it had an average number of reads mapping to peak regions ≥ 10,000 and the number of cells was between 2000 and 5000. There were only four multiome samples available when we performed the integration analyses; therefore, no sample selection was applied for the multiome data. In total, we included 10 scRNA-seq, 8 snATAC-seq, and 4 multiome samples generated from 16 unique donors. The overlap of donors is shown in Fig. 6B, and sequencing details are shown in Table 1.

We processed the three datasets internally and generated count matrices that were then passed through each integration method being evaluated. For the scRNA-seq data, raw FASTQ files were processed with Cellranger-7.1.0, to generate cell-by-gene counts table, which included intronic and exonic reads while tabulating transcript counts. We removed ambient RNA with SoupX [27], and doublets with scDblFinder [28]. Features expressed by less than 3 cells were removed. Cells with less than 200 or more than 10,000 features, with percentage of mitochondrial reads > 25%, or number of UMI counts less than 500 or more than 100,000 were removed. For the snATAC-seq data, the cell-peak count matrix was tabulated using the Signac package [24]. Specifically, FASTQ files were processed with Cellranger-atac-2.0.0. Outputs from cellranger-atac were used to generate a cell-peak counts matrix that tabulate the number of open chromatin regions observed in the peak regions for every cell. The peak set used was the peaks called using the four multiome samples. Cells with less than 1000 or more than 100,000 reads mapped in peak regions had nucleosome signal greater than 2 or TSS enrichment less than 1 were filtered out. For the multiome data, FASTQ files were processed with Cellranger-arc-1.0.0. Cells were filtered by both RNA and ATAC datasets, in a sample-specific manner. Specifically, cells with nucleosome signal > 2, TSS enrichment < 1, percentage of mitochondrial reads > 30%, or number of reads mapped to genes or peaks less than 1000 or greater than 5 median absolute deviation from the median, were removed. Peaks were called for each sample individually with MACS2 [29] and then merged. Lastly, doublets were removed with scDblFinder [28].

### Evaluation metrics

#### Annotation accuracy

Each integration method returns an integrated latent embedding matrix for cells. Louvain clustering was performed to identify *k* clusters, in which *k* is the number of cell types in the ground-truth annotation. To evaluate annotation accuracy, Adjusted Rand Index (ARI) [17] and Normalized Mutual Information (NMI) [18] from the Scib package (v1.0.2) [21] were calculated to compare the predicted cluster labels with the ground truth. Specifically, ARI compares every pair of cells in the dataset and calculates a similarity measurement by considering the number of cell pairs that are in the same cluster in both annotation results, versus the number of cell pairs showing discordant annotations. This metric is then adjusted by chance, as there will be a non-zero similarity between the two clustering results just due to random permutation of labels. The resulting metric ranges from 0 to 1 in which 1 means perfect matching between the two results while 0 means random labeling of cells. NMI is another measurement commonly used for comparison of two clustering results. NMI measures if knowing one label provides information about the other label. If the two lists are highly correlated, then it has high mutual information. NMI is then normalized by a factor to control for differences due to the number of clusters in each set of labels.

#### Cell type separation

We evaluated the separation of clusters and the tightness of cells in the integrated latent space derived from each method. We calculated cell type-specific average silhouette width (ASW) [21], using the ground-truth annotation and the joint embeddings. The resulting score is between 0 and 1 in which 1 means small intra-cluster distance and high inter-cluster distance. We also calculated a cell type Local Inverse Simpson’s Index (cLISI) [21], which is an adaptation of LISI previously used to quantify the degree of batch effects [20]. Here, cLISI was calculated using the ground-truth labels again in which it evaluates how many cells need to be drawn from a cell’s neighborhood to draw a second cell of the same type. The score is normalized again so that 1 means good local neighborhood preservation of the same cell type while 0 is otherwise.

#### Batch mixing

To evaluate batch mixing, two metrics were employed. A batch ASW score was used to evaluate the within-batch distance and the across-batch distance [21]. The score was rescaled so that 0 is the worst and 1 is the best separation. To evaluate the local neighborhood accuracy, *k*-nearest neighbor batch effect test (kBET) was also performed [19]. Specifically, kBET measures the difference between observed batch frequency in the *k*-nearest neighbors compared to an expected frequency based on the number of cells in each batch. The value is rescaled to 0 and 1 in which 1 represents the optimal mixing of cells from different batches in which cells in the neighborhood are highly similar to the expected frequency.

#### Peak-gene pair recovery

To identify correlated peak-gene pairs, we used the methodology introduced in the SHARE-seq paper [1]. Specifically, a Pearson correlation is calculated between the raw accessibility count of every peak and the normalized UMI count of every gene if the peak is within 50,000 base pairs from the transcription start site (TSS) of the gene. The null distribution of correlation coefficients was then generated through selecting 100 peaks that have similar GC content, length, and accessibility as the target peak, and calculating correlation of the background peaks and the target gene. A one-sided *t*-test was used to calculate a *p*-value for every peak-gene pair by comparing to the background peaks and the peak-gene pairs with *p*-value less than 0.05 and *z*-score greater than 0.05 identified as significant peak-gene pairs. Associated peak-gene pairs were identified using all cells from each dataset. To evaluate the performance of each method at imputing gene expression from snATAC-seq data, a peak-gene association was calculated in the same manner using the raw cell-peak count of the unpaired ATAC data and the predicted gene expression generated by the evaluated methods. To evaluate the in silico imputed gene expression results, we calculated the percentage of peak-gene pairs recovered using the imputed gene expression and the observed snATAC-seq peak counts. To account for false negative results, we calculated an F1 score. Thus, the peak-gene pair percent recovery and the F1 score were used to evaluate each method that can impute missing gene expression.

#### Rare cell type recovery

To assess the ability of a method in identifying rare cell populations, we computed an F1 score. For each method, we first perform clustering analysis. Since the ground-truth cell type identity is known, for clusters that contain the target rare cell population, we calculate the F1 score for that cluster, which considers the number of true positives (TP), false positives (FP), and false negatives (FN). The F1 score is calculated as follows: F1 = TP / (TP + 0.5 × (FP + FN)). Therefore, for each method in each scenario, we have a list of F1 scores, with each corresponding to one cluster being the rare cell type. We then select the highest F1 score to represent the performance of this method in this scenario. Through visual inspections, we determined that an F1 score greater than 0.8 indicates a good identification of the rare cell population. Conversely, an F1 score below 0.5 suggests poor identification, as it means that there are twice as many false positives or false negatives compared to true positives.

### Evaluation scenarios

We simulated five scenarios to evaluate the performance of each method. For each scenario, we simulated five independent replicates. Details regarding how each method was implemented are described in the Additional file 1: Supplementary methods.

#### Scenario 1: evaluating the effect of multiome cells on single-modality integration

##### Data simulation

In this task, we first defined the number of cells to be drawn for each data type with an example shown in Fig. 2A. Then, we randomly selected cells from the ground-truth multiome dataset according to the desired number of cells for each data type. For scRNA-seq, we kept the gene expression matrix; for snATAC-seq, we kept the cell-by-peak matrix and the fragment file; lastly, for the multiome sample, we kept all three data files. The cells were sampled without replacement.

##### Evaluated methods

We first ran the five unpaired integration methods (Seurat v3, LIGER, FigR, BindSC, and GLUE) to integrate the simulated scRNA-seq and snATAC-seq datasets and the results were summarized under the “Unpaired” categories. To make use of the multiome data, we ran the five methods again, with the multiome cells treated as unpaired. Specifically, the RNA profile from the multiome cells was appended to the scRNA-seq dataset, and the ATAC-seq profile was appended to the snATAC-seq dataset. The results from this category were summarized under “Unpaired (multiome-split).” Lastly, we ran the multiome-guided methods with the scRNA-seq, snATAC-seq, and multiome datasets as input.

##### Evaluations

To evaluate if the presence of multiome cells improves the integration of single-modality datasets, we evaluated the annotation accuracy, peak-gene pair recovery, cell type separation, and batch mixing of the scRNA-seq and snATAC-seq cells.

#### Scenario 2: evaluating the impact of sequencing depth in multiome cells on multi-omic data integration

##### Data simulation

For this task, we first defined the number of cells in each data type as well as the percentage of original depth the multiome cells will be downsampled to; an example is shown in Fig. 4A. We first generated the three data types according to the number of cells defined. Then, we performed depth-downsampling for both the gene expression and chromatin accessibility profiles of the multiome dataset. To downsample the cell-by-gene count matrix for gene expression, we used Scuttle::downsample [30] to reduce the sample depth to a percentage of the original dataset. To downsample the ATAC-seq depth, we performed downsampling on the fragment file and then regenerated the cell-by-peak count matrix. Specifically, we first counted the number of fragments corresponding to the selected cells, then we calculated the target depth by multiplying the original depth to the percentage factor. We randomly selected the number of reads as calculated, without replacement, and saved this file as the new fragment file. Then the downsampled fragment file was sorted, recompressed, indexed with tabix, and tabulated into peak counts with the original feature set with Signac:: FeatureMatrix [24] function. This often resulted in less reduction in peak counts, as some of the fragments removed were not previously assigned to the peaks.

##### Evaluated methods

We ran the unpaired integration methods with the multiome data appended to the single-modality datasets as described above, the results were summarized under “Unpaired (multiome-split).” We also ran the three multiome-guided methods.

##### Evaluations

The evaluation of annotation accuracy, cell type separation, and batch mixing were calculated using all cells present in simulated scRNA-seq, snATAC-seq, and the multiome datasets. Given how the multiome data were split and appended to the single-modality datasets for the “unpaired (multiome-split)” category, it resulted in doubling the number of multiome cells. Thus, to ensure a fair comparison between the two categories of methods, half of the multiome cells appended to the RNA-seq were dropped while the other half of the multiome cells appended to the ATAC-seq were dropped. As a result, the same number of cells was evaluated for the “unpaired (multiome-split)” and “multiome-guided” methods.

#### Scenario 3: evaluating the impact of batch effects on multi-omic data integration

##### Data simulation

The analysis of batch effects was only possible for the BMMC dataset. As mentioned before, the BMMC dataset contains multiome cells generated at four different research sites and nine donors. To create different types of batches, we used the multiome cells from donor 1 but processed at three different sites (S1D1, S2D1, S4D1) as the data source to generate technical batches. We used the multiome cells generated at research site 1 but from different donors (S1D1, S1D2, S1D3) as the source of biological batches. To generate scenarios with mixed technical and biological batch effects, we created more complex batch structures described as “complex test” in Fig. 5D using all samples that were either generated at research site 1 or donor 1. After defining which sample each data type comes from and the number of cells, the simulation is the same as described in “Scenario 1,” in which cells were randomly drawn from the ground-truth multiome dataset to simulate scRNA-seq, snATAC-seq, and multiome samples.

##### Evaluated methods

The same seven methods, four from the “unpaired (multiome-split)” and three from “multiome-guided” were ran. For situations were multiome were composed of two donors, an additional variation of Seurat v4 was added, termed “Seurat v4 integrate.” Specifically, the two multiome datasets were first integrated across donors to generate one integrated reference before it was used to integrate scRNA-seq and snATAC-seq datasets.

##### Evaluations

We calculated metrics measuring annotation accuracy, cell type separation, and batch mixing. For batch mixing, we calculated both the mixing of data types, as well as the mixing of samples. Similar to what was described in “Scenario 2,” to ensure that the same number of cells were evaluated for the unpaired (multiome-split) methods and the multiome-guided methods, half of multiome cells appended to the RNA-seq and the other half of the ATAC-seq dataset were dropped.

#### Scenario 4: evaluating the recovery of a rare cell population when it is not present in one of the single-modality datasets

##### Data simulation

The PBMC and SHARE-seq datasets were used for this task. For the PBMC dataset, we chose NK cells to be the rare cell type that may not be present in all three datasets. The simulation process was similar to scenario 1 where the source dataset was split into scRNA-seq, snATAC-seq, and multiome datasets but in addition, we specified if each data type has the target cell type (e.g., NK cells for the PBMC dataset). If yes, cells from the target cell group were first sampled to represent the exact same percentage as the source dataset. In the PBMC dataset, NK cells account for 4.6% of the whole population, thus, for scRNA-seq and snATAC-seq, which were 1000 cells in total, we sampled 46 NK cells. For the multiome dataset, depending on the total number of cells, 0.046 × total number of multiome cells were first sampled before sampling the cells from the other cell types. In situations where the specific data type was missing NK cells, cells were sampled from the source dataset excluding the target cell type. For the SHARE-seq dataset, a similar simulation process was carried out. The only difference was that we downsampled the SHARE-seq dataset to 10,000 cells. Specifically, we first filtered out cell types with less than 500 cells, and then sampled 10,000 cells from the source dataset without replacement, constructing a dataset where “Hair Shaft (HS)” cells account for 10% and “Endothelial (Endo)” cells account for 5% of the total population.

All evaluated scenarios can be found in Fig. 7; there’s an illustration next to each result indicating what cell types were missing and which dataset was evaluated. For the PBMC dataset, we simulated these situations: (1) all three data types had the NK cells (“No missing”), snATAC-seq did not have NK cells (“snATAC missing NK”), and (2) scRNA-seq did not have NK cells (“scRNA missing NK”). For the simulations with the SHARE-seq dataset, we again had scRNA-seq or snATAC-seq missing Endo cells or HS cells, but in addition, we had situations where a single-modality dataset did not have both cell types (“snATAC missing HS & Endo” and “scRNA missing HS & Endo”).

##### Evaluated methods

The same methods as described in scenario 1 were ran here. Specifically, we ran all methods belonging to the “unpaired integration,” “unpaired (multiome-split),” and “multiome-guided integration” categories.

##### Evaluations

For each simulation, we calculated the rare cell type recovery score for scRNA-seq cells and snATAC-seq cells separately. For example, in Fig. 7A (left), we computed the recovery of NK cells in the scRNA-seq dataset when the snATAC-seq does not have the NK cells. We compared this score with the score calculated using the scRNA-seq cells under the “No Missing” scenario. This ensured that the same number of cells was used for the metric calculation, and we were only evaluating when certain datasets missed the target cell population.

The rare cell type recovery score used here was essentially an F1 score. We calculated the number of cells annotated correctly as the rare cell population as well as the number of false positives and false negatives. See the “Rare cell type recovery” section of the “Evaluation Metrics” for more details on the specific calculations.

#### Scenario 5: evaluating the recovery of a rare cell population when it is not present in the multiome dataset and/or a single-modality dataset

##### Data simulation

Similar simulation steps as scenario 4 were carried out here, except we evaluated the effect of the multiome dataset lacking the target cell type. Using the PBMC dataset and NK cells as an example, the situations tested were as follows: (1) all three data types had the target cell type (“No missing”), and the multiome dataset did not have the NK cells (“Multiome missing NK”), and (2) multiome and one of the single-modality datasets did not have the NK cells (“Multiome and snATAC missing NK” and “Multiome and scRNA missing NK”). The same setup was replicated for the SHARE-seq dataset for HS cells and Endo cells separately.

##### Evaluated methods

Methods belonging to the “unpaired integration” and “multiome-guided integration” categories were run. In these scenarios, the multiome dataset did have the target cell population, thus, the “unpaired (multiome-split)” methods should have the same result as the “unpaired integration” methods.

##### Evaluations

Similar to scenario 4, F1 scores were calculated for scRNA-seq cells and snATAC-seq cells separately for each method under each simulated situation.

### Supplementary Information

**Additional file 1.** Includes supplementary figures demonstrating the performance of all methods being evaluated under each simulated scenario. Additional file 1 also includes supplementary methods that documents how each integration method was carried out.

**Additional file 2.** Review history.

## Data Availability

The source codes for simulation and evaluations are available online under the MIT license on GitHub at https://github.com/myylee/benchmark_sc_multiomic_integration [31] and on Zenodo at https://zenodo.org/record/8353265 [32]. For the multiome datasets used to generate simulated data, the 10 × PBMC dataset was downloaded from https://www.10xgenomics.com/resources/datasets/pbmc-from-a-healthy-donor-granulocytes-removed-through-cell-sorting-10-k-1-standard-2-0-0 [13]. The BMMC dataset [14, 15] was downloaded from GEO accession GSE194122 [33], the fragment files were obtained from the authors [14, 15]. The SHARE-seq dataset [1] was downloaded from GEO series GSM4156608 [34] and GSM4156597 [35]. HPAP datasets [26] were downloaded from https://hpap.pmacs.upenn.edu [36]. No custom scripts and software other than those mentioned in the “Methods” section were used in this study.
