# Supplementary material for: Benchmarking algorithms for joint integration of unpaired and paired single-cell RNA-seq and ATAC-seq data
Source: Genome Biol. 2023 Oct 24;24:244. doi: 10.1186/s13059-023-03073-x (PMC10594700; doi:10.1186/s13059-023-03073-x)
Supplement: Supplementary file 1 — Additional file 1. Includes supplementary figures demonstrating the performance of all methods being evaluated under each simulated scenario. Additional file 1 also includes supplementary methods that documents how each integration method was carried out. [file 13059_2023_3073_MOESM1_ESM.pdf]

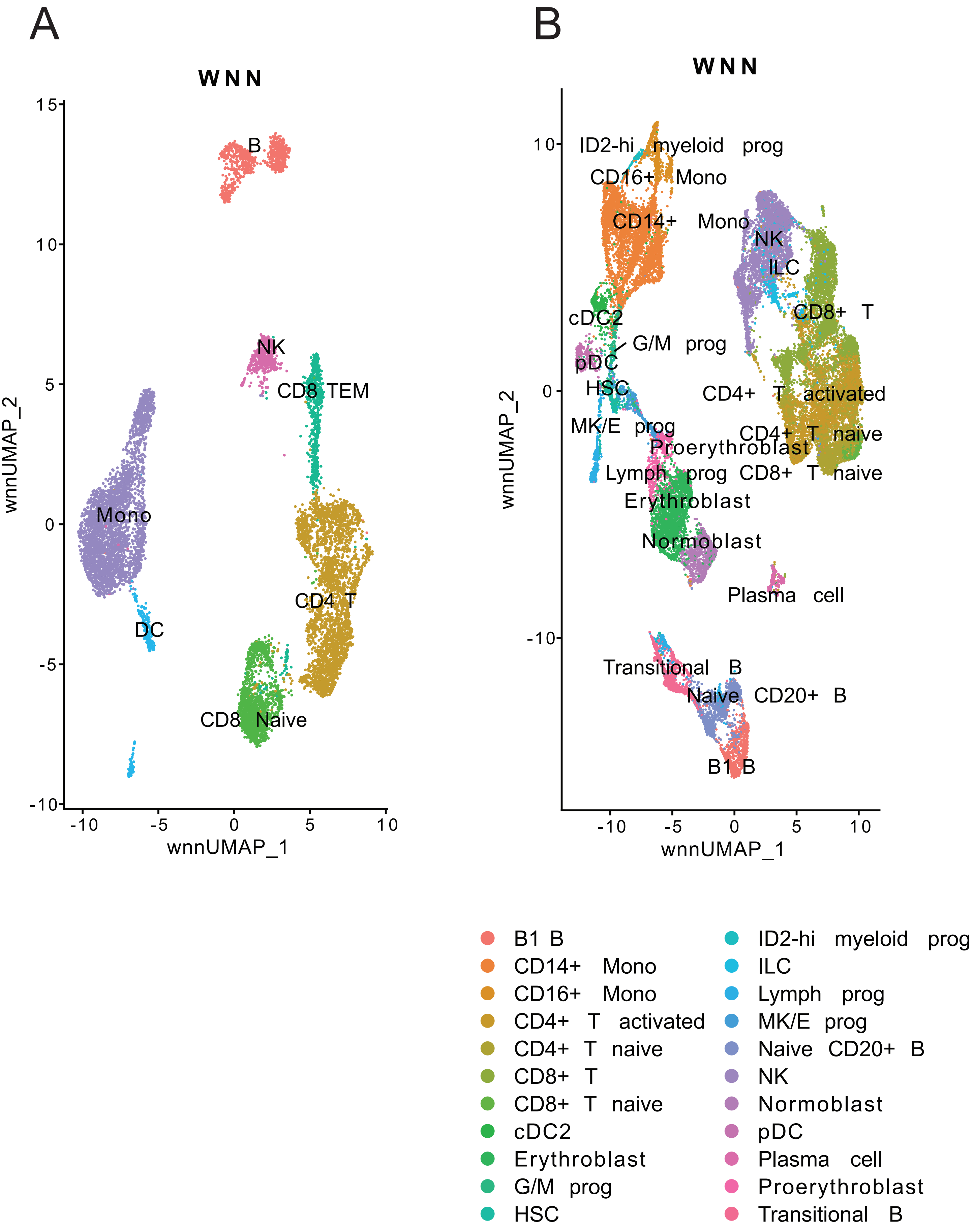

Fig. S1: UMAP plots of (A) PBMC dataset and (B) BMMC dataset. Cells are colored by cell types.

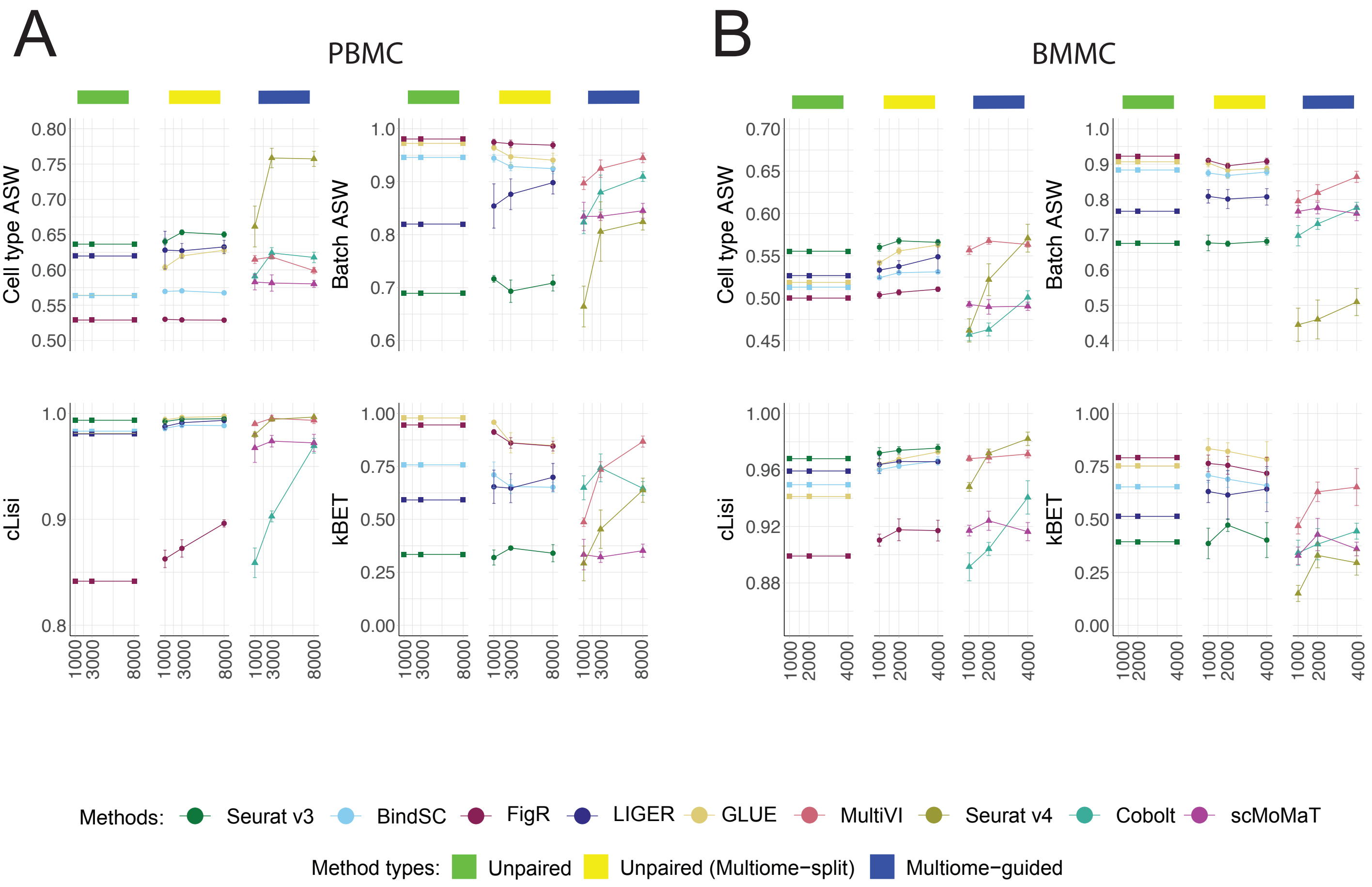

Fig. S2: Additional evaluation metrics for each method at integrating single-modality cells in the presence of multiome data, as described in Figure 2. (A) PBMC-based simulations. (B) BMMC-based simulations. Cell type average silhouette width (ASW) and cell type Local Inverse Simpson's Index (cLISI) measure separation of cell types. Batch ASW and k-nearest n eighbor batch effect test (kBET) measure the mixing of scRNA-seq and snATAC-seq cells. Error bar is mean ± standard deviation.

PBMC varying number of multiome cells

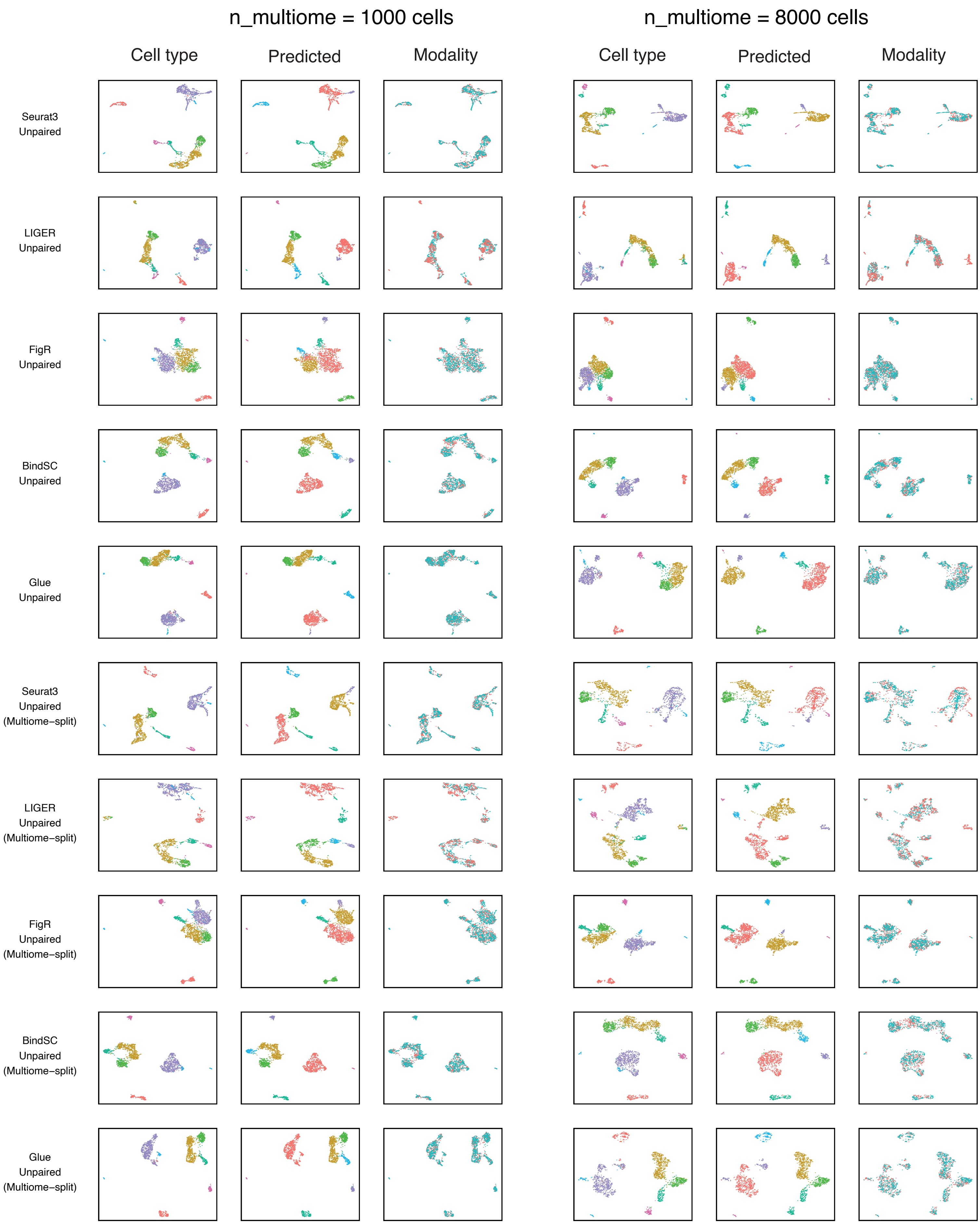

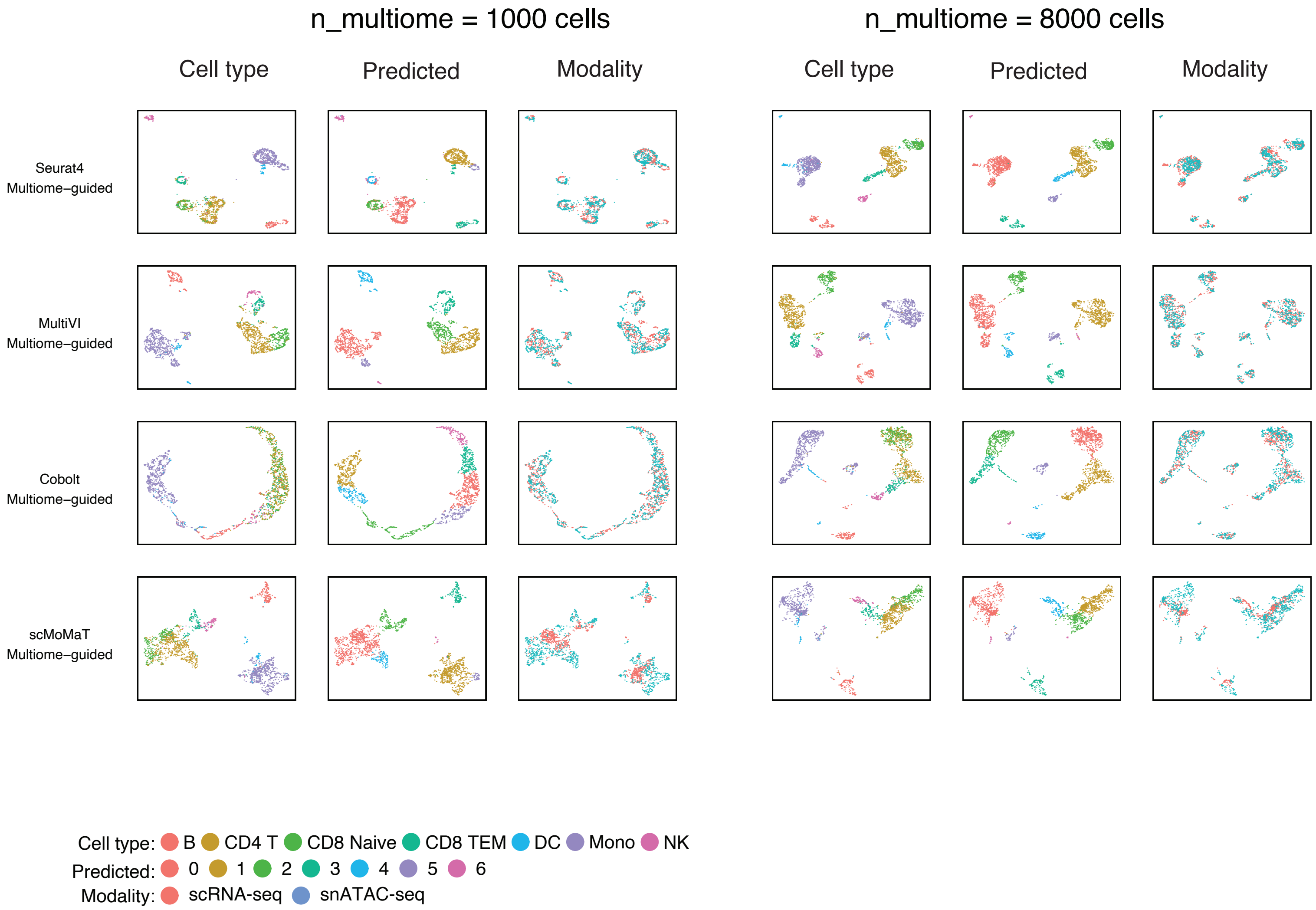

Fig. S3: UMAP plots for the PBMC-based simulations shown in Figure 2B.

BMMC varying number of multiome cells

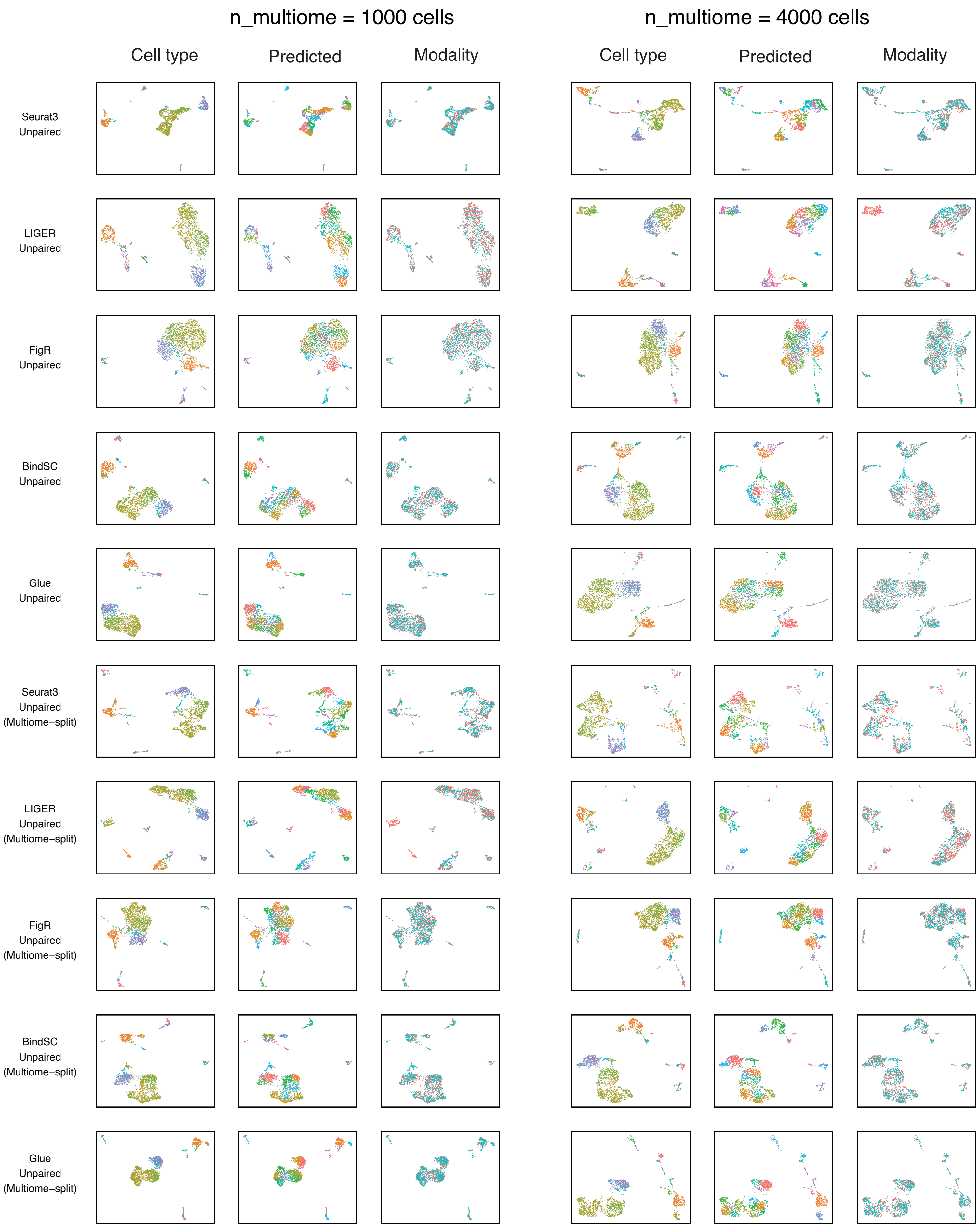

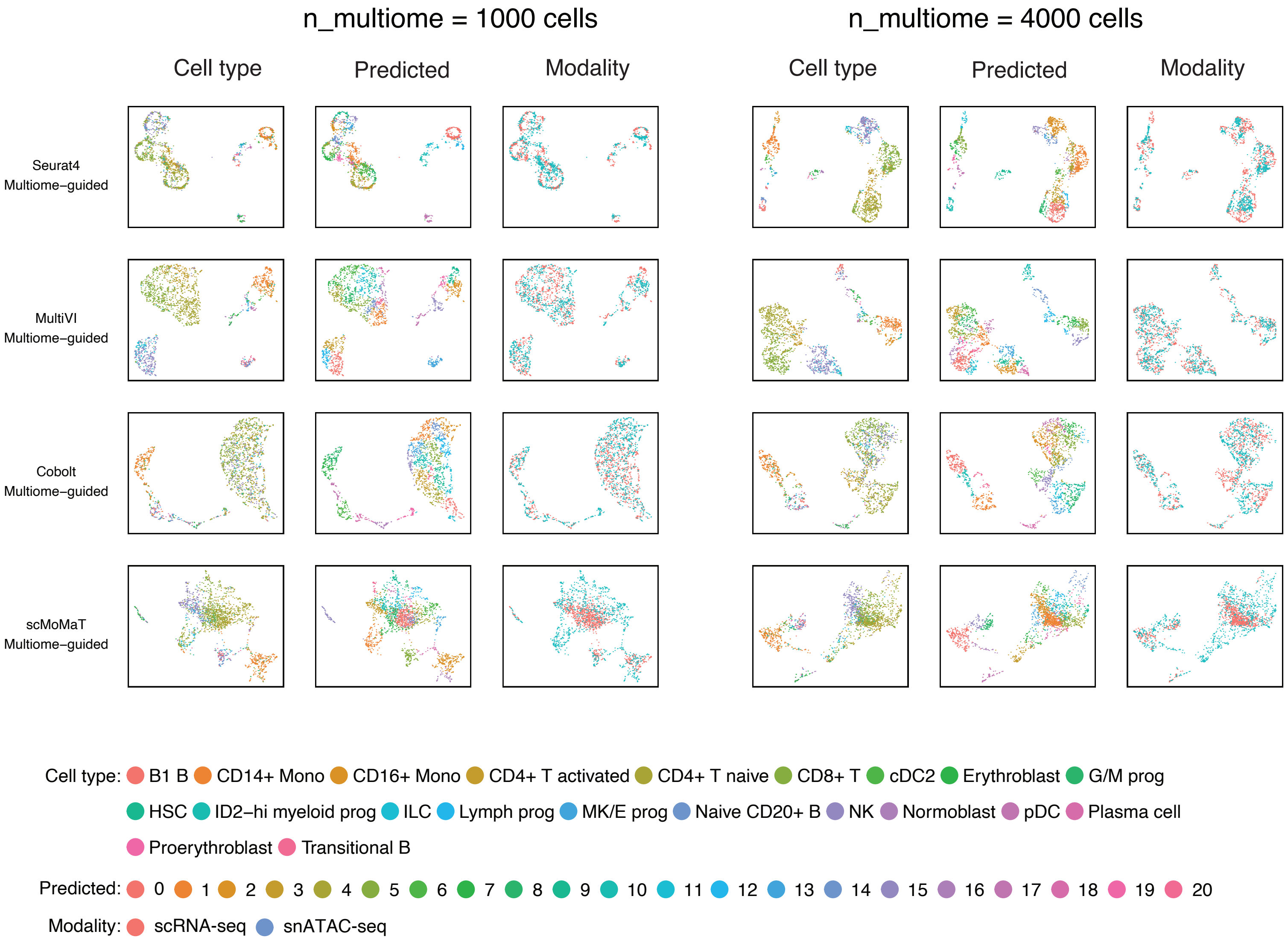

Fig. S4: UMAP plots for the BMMC-based simulations shown in Figure 2C.

A

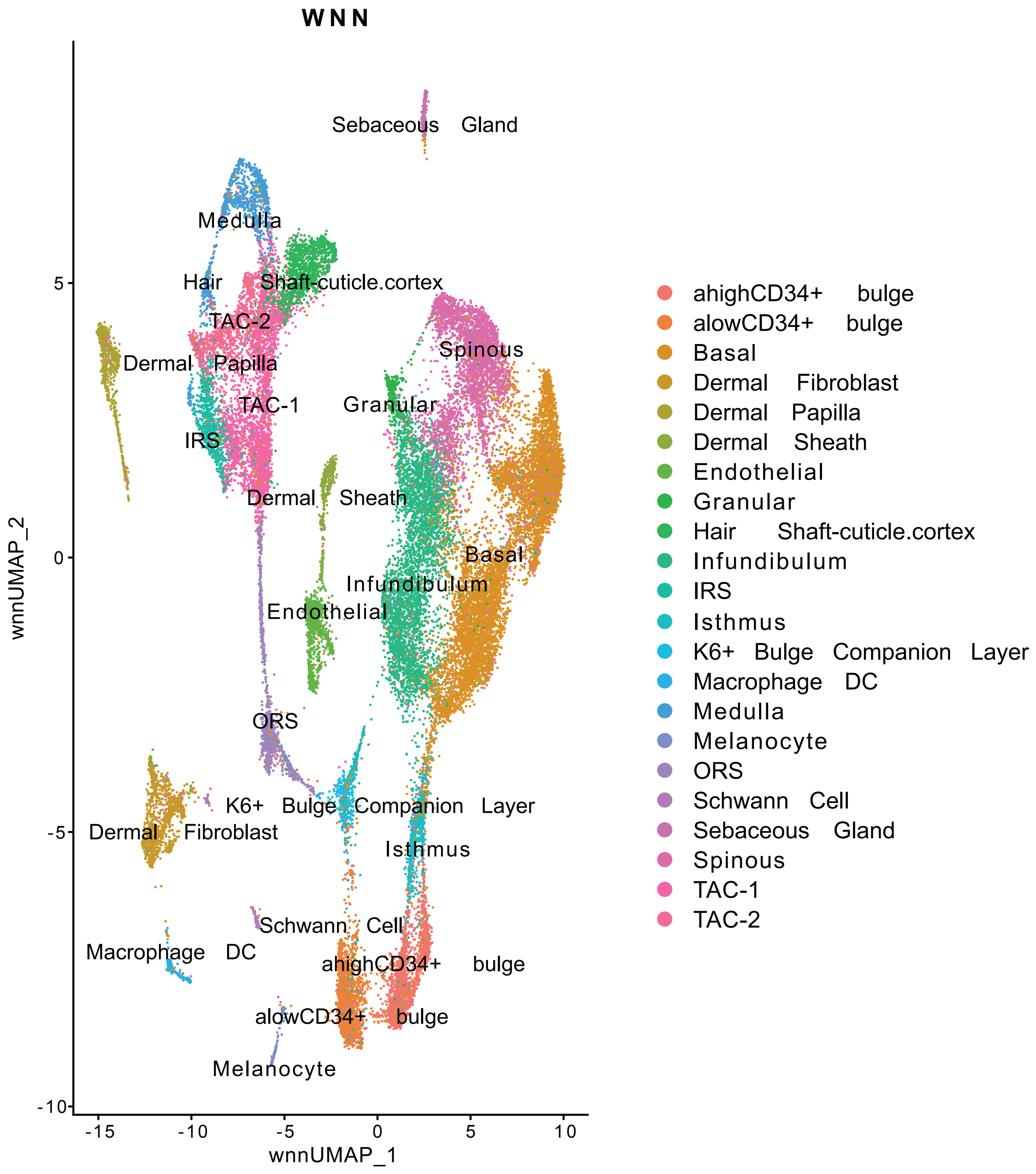

Fig. S5: UMAP plot of the SHARE-seq mouse skin dataset, colored by cell types.

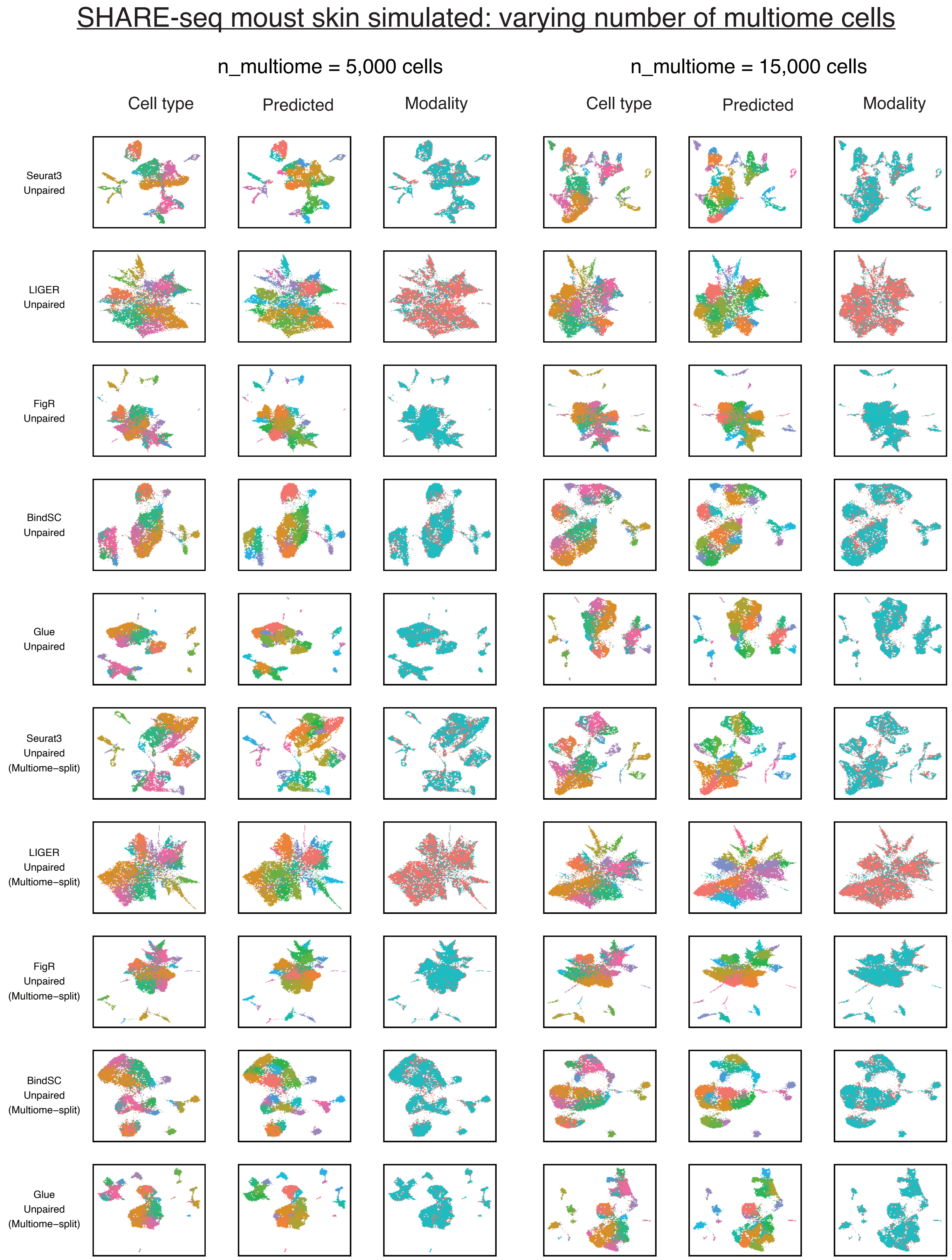
SHARE-seq moust skin simulated: varying number of multiome cells
n_multiome = 5,000 cells
n_multiome = 15,000 cells
Cell type
Predicted
Modality
Seurat3 Unpaired
LIGER Unpaired
FigR Unpaired
BindSC Unpaired
Glue Unpaired
Seurat3 Unpaired (Multiome−split)
LIGER Unpaired (Multiome−split)
FigR Unpaired (Multiome−split)
BindSC Unpaired (Multiome−split)
Glue Unpaired (Multiome−split)

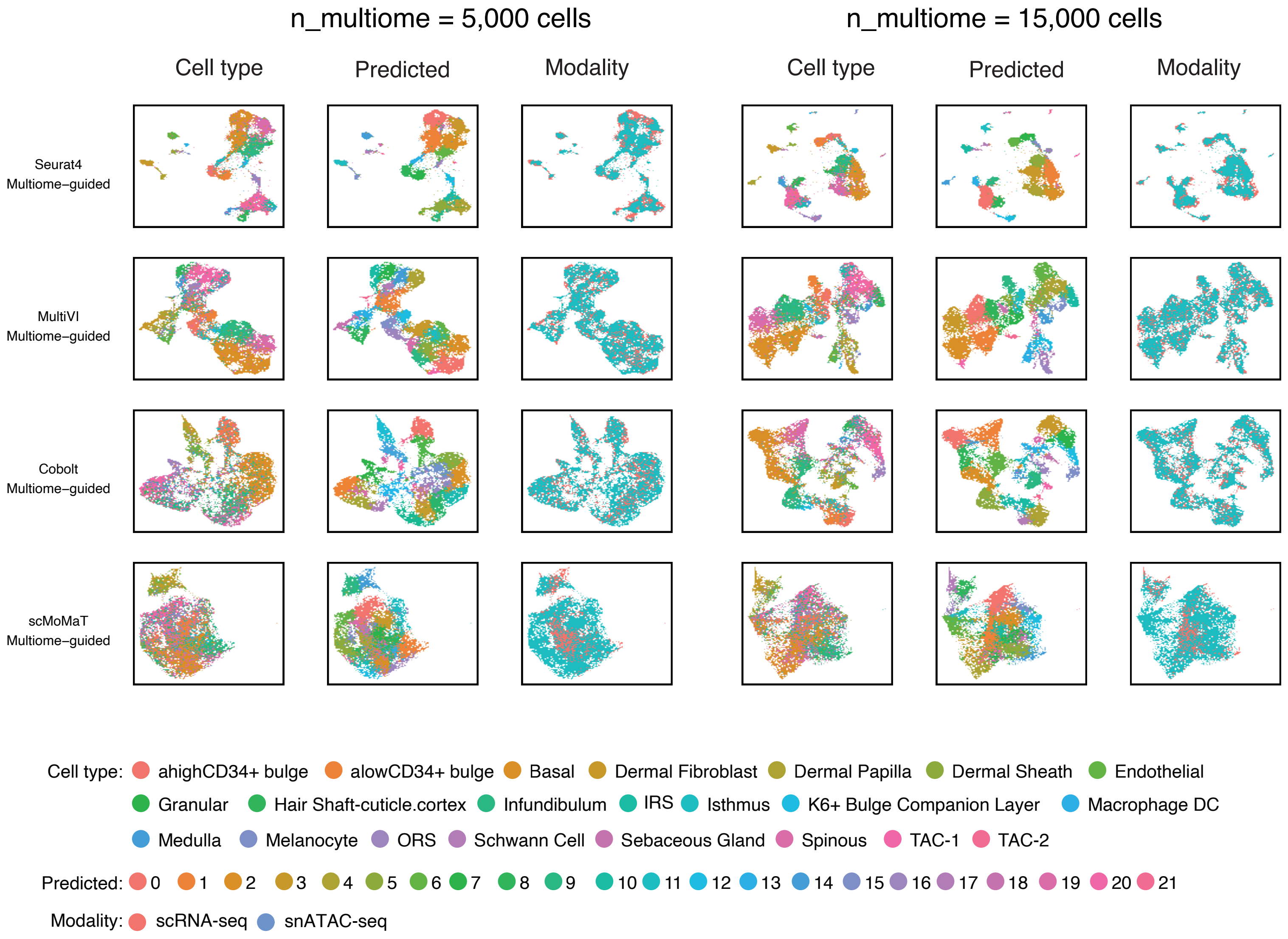

Fig. S6: UMAP plots for the SHARE-seq based simulations shown in Figure 3.

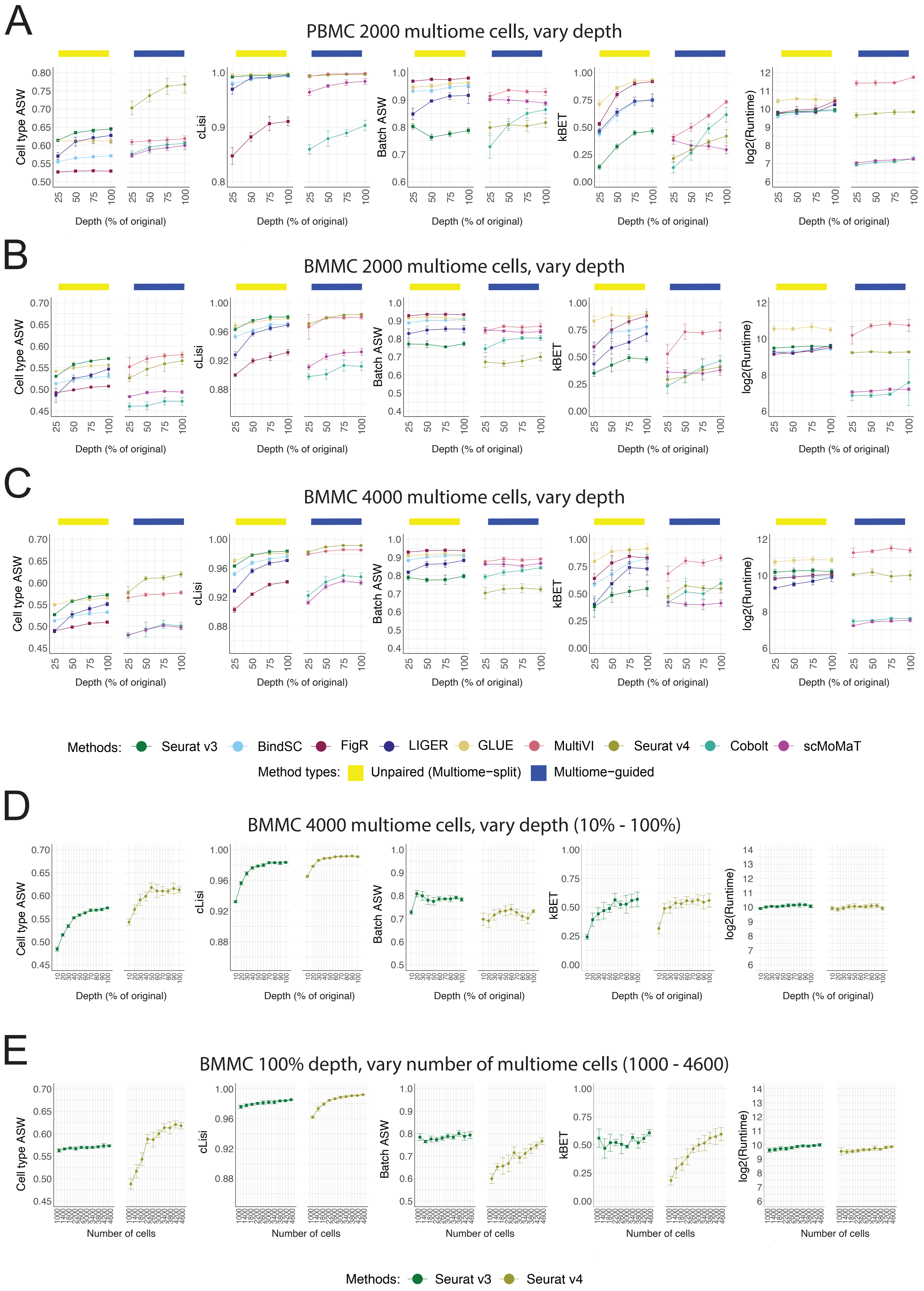
A
PBMC 2000 multiome cells, vary depth
B
BMMC 2000 multiome cells, vary depth
C
BMMC 4000 multiome cells, vary depth
D
BMMC 4000 multiome cells, vary depth (10% - 100%)
E
BMMC 100% depth, vary number of multiome cells (1000 - 4600)
Cell type ASW
cLisi
Batch ASW
kBET
log2(Runtime)
Depth (% of original)
Number of cells
Methods: Seurat v3 BindSC FigR LIGER GLUE MultiVI Seurat v4 Cobolt scMoMaT
Method types: Unpaired (Multiome−split) Multiome−guided
Methods: Seurat v3 Seurat v4

Fig. S7: Additional evaluation metrics for each method at integrating scRNA- seq, snATAC-seq and multiome data, at different depths of the multiome data, as described in Figure 3. (A) PBMC-based simulations. (B) BMMC-based simulations with 2000 multiome cells. (C) BMMC-based simulations with 4000 multiome cells. (D) BMMC-based simulations with 4000 cells at 10 different depths. (E) BMMC-based simulations at 100% of depth but with 10 different numbers of multiome cells. Cell type ASW and cLISI measure separation of cell types. Batch ASW and kBET measure the mixing of scRNA-seq, snATAC-seq, and multiome cells. Runtime is measured in seconds, for each method, in log2 scale. Error bar is mean ± standard deviation.

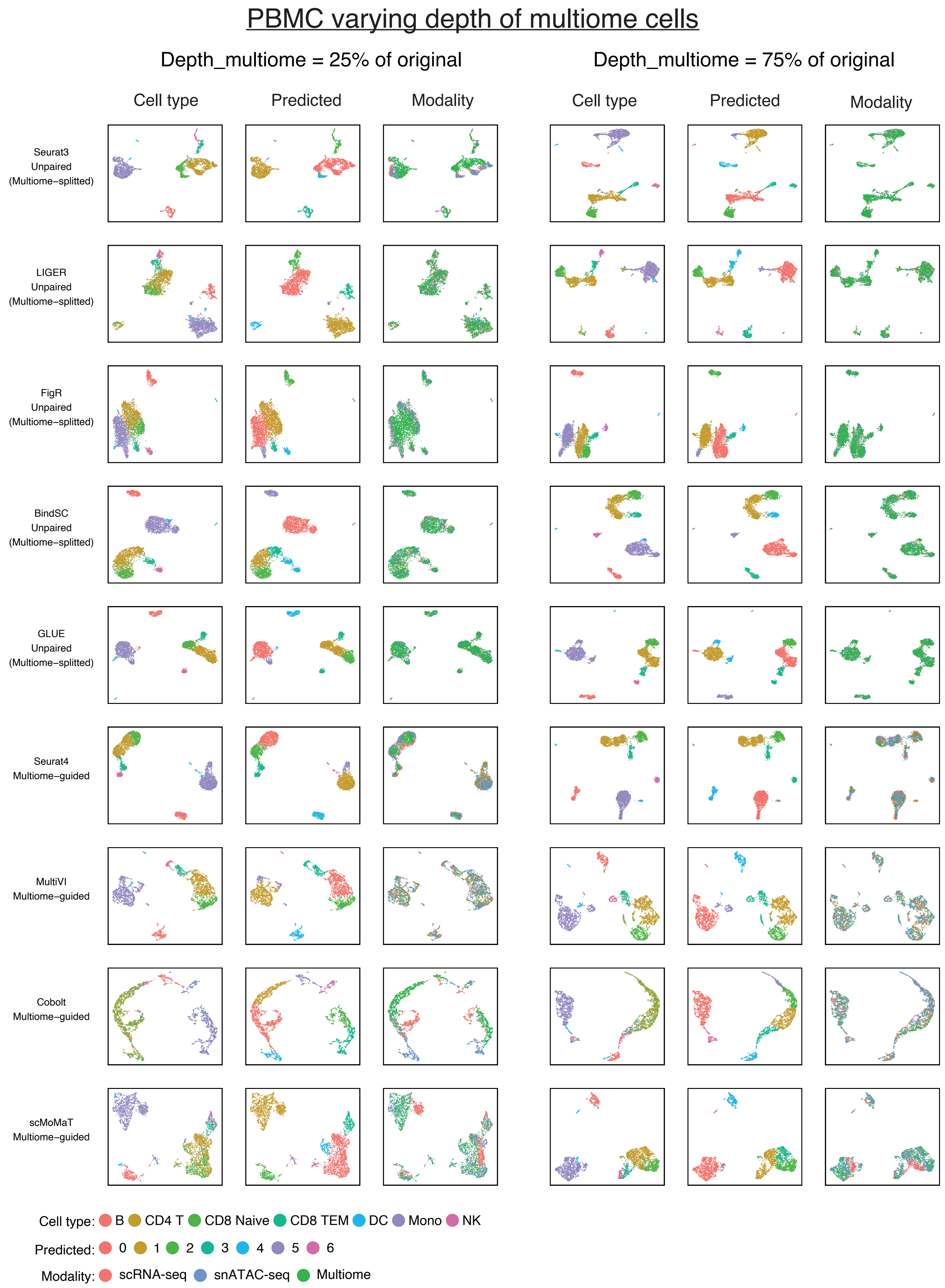

Fig. S8: UMAP plots for the PBMC-based simulations shown in Figure 4B.

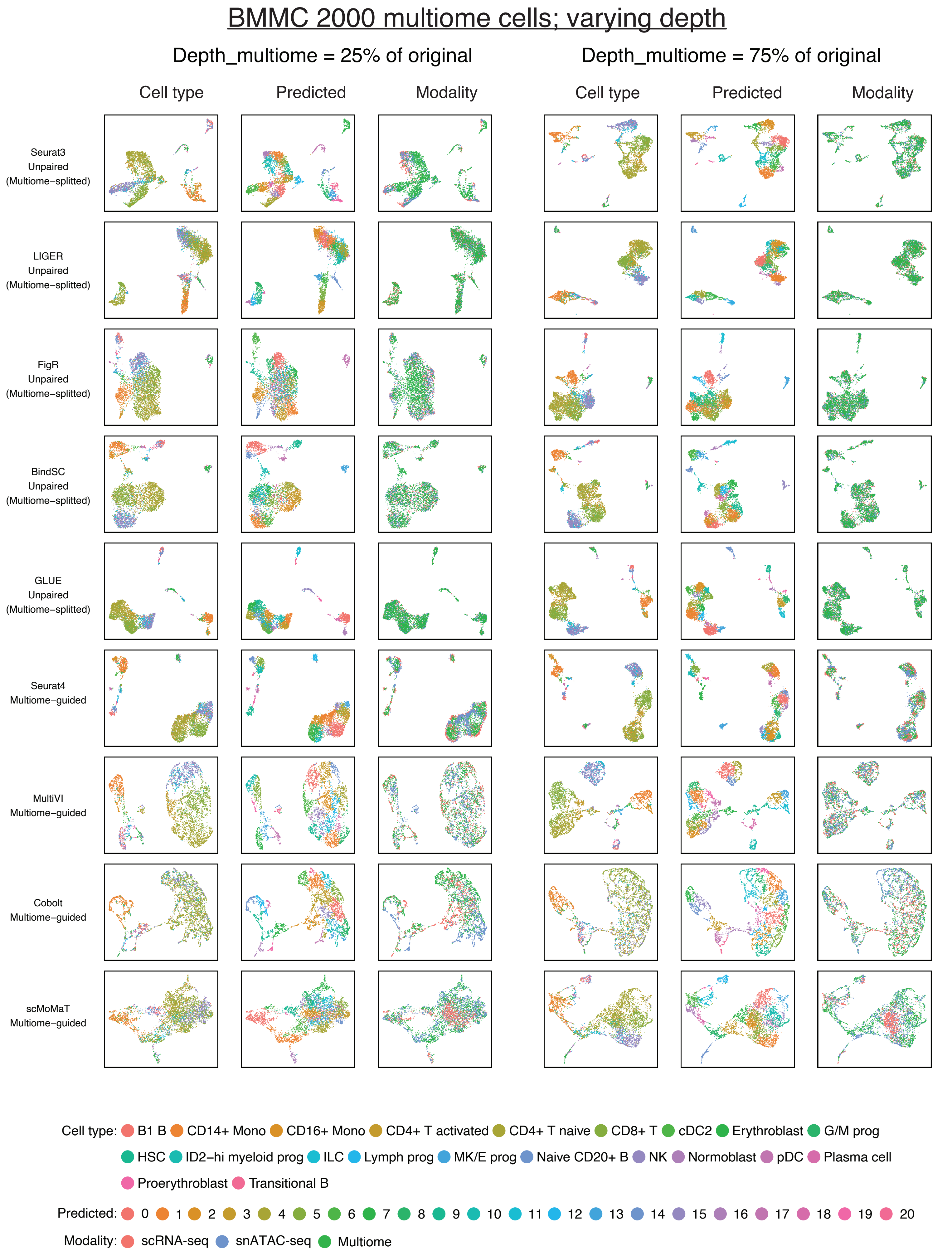

Fig. S9: UMAP plots for the BMMC-based simulations with 2000 multiome cells shown in Figure 4C (left).

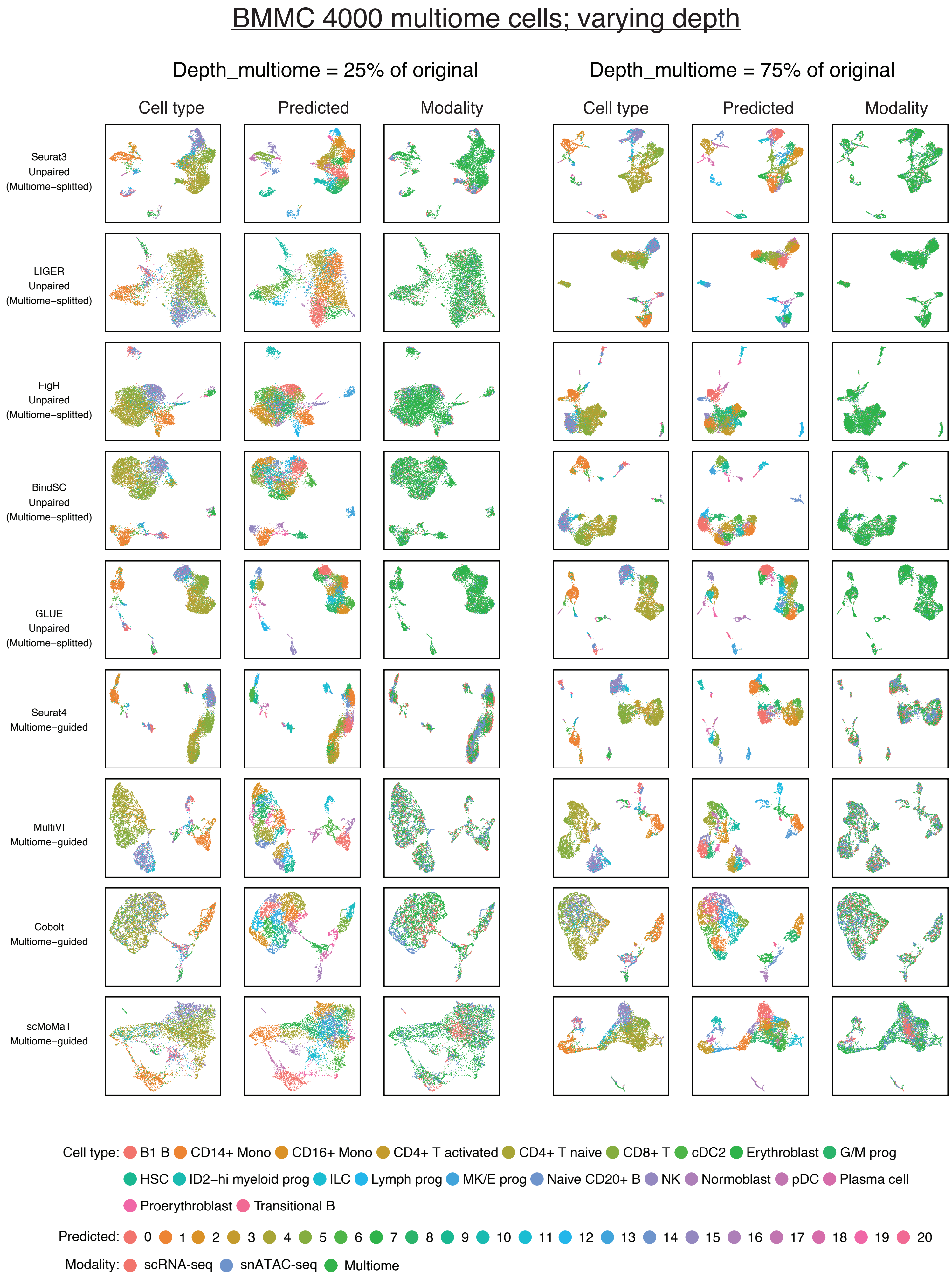

Fig. S10: UMAP plots for the BMMC-based simulations with 4000 multiome cells shown in Figure 4C (right).

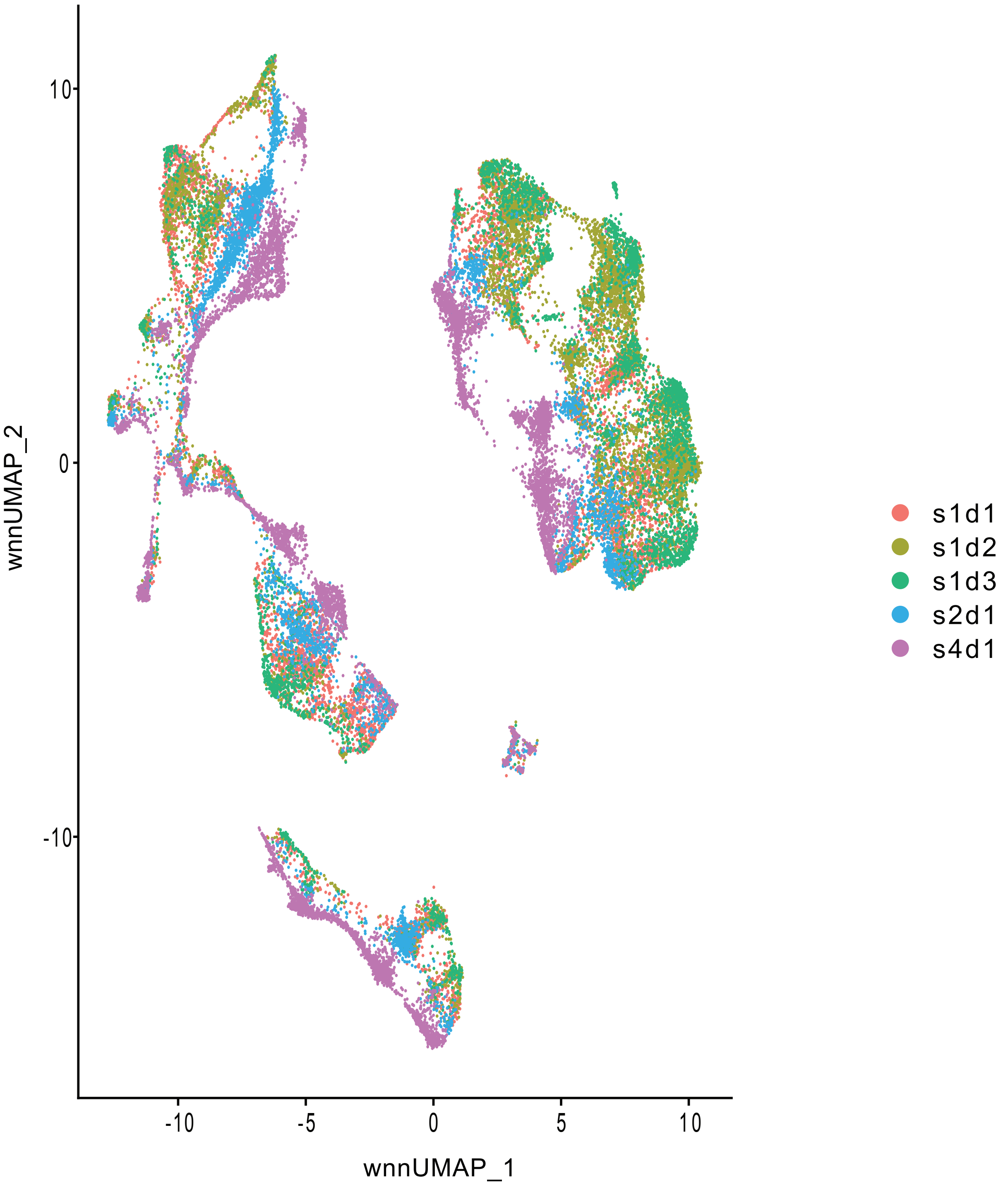

Fig. S11: UMAP plots colored by sample origins, containing 5 BMMC samples generated from research site 1 or donor 1 samples.

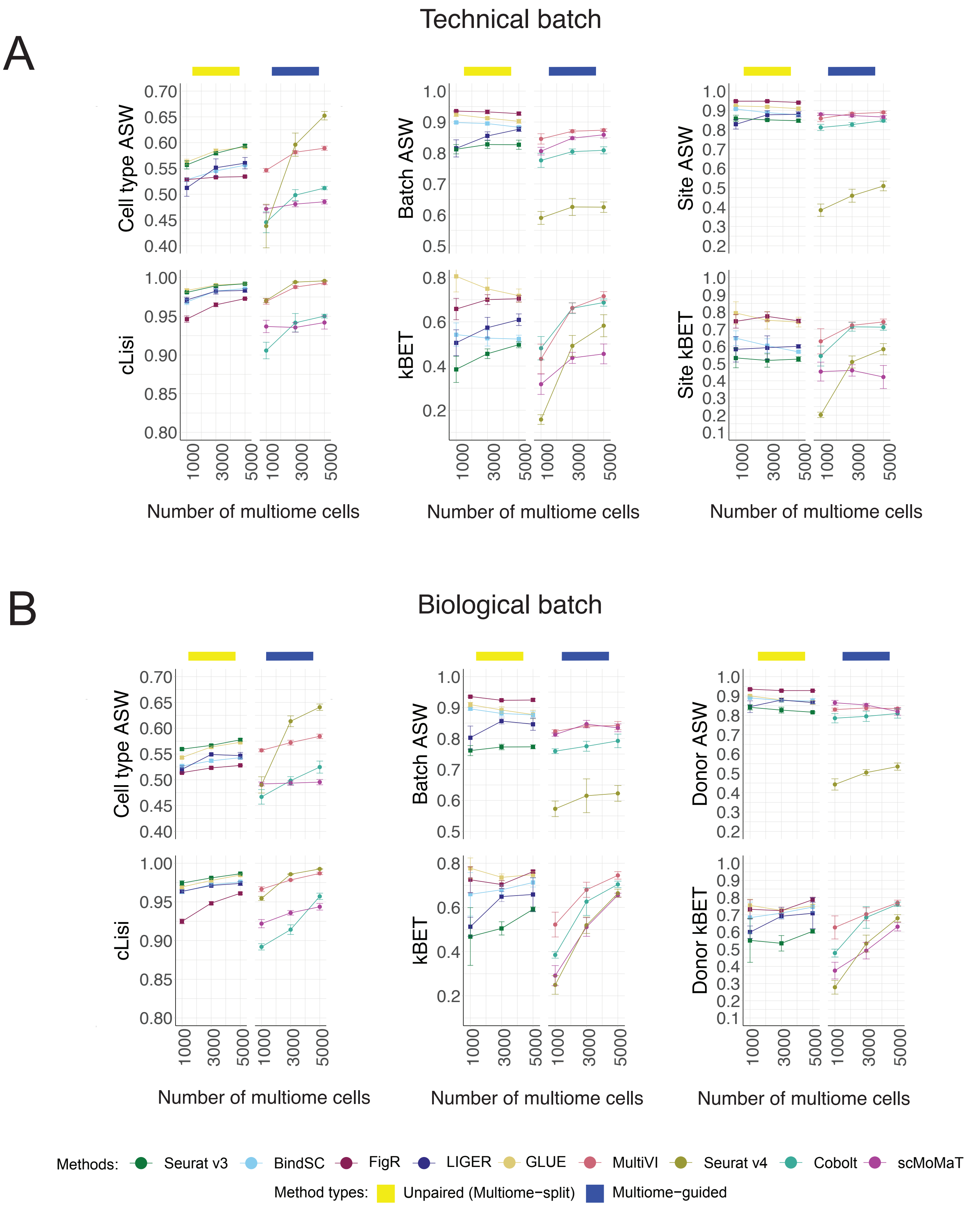

Fig. S12: Additional evaluation metrics for each method at integrating scRNA-seq, snATAC-seq and multiome data, in the presence of batch effects as described in Figure 5B. (A) Technical batch effect (Figure 5B left). (B) Biological batch effect (Figure 5B right). Cell type ASW and cLISI measure separation of cell types. Batch ASW and batch kBET measure the mixing of scRNA-seq, snATAC-seq, and multiome cells. Site ASW and site kBET measure the mixing of cells by site ID. Donor ASW and donor kBET measure the mixing of cells by donor ID. Error bar is mean ± standard deviation.

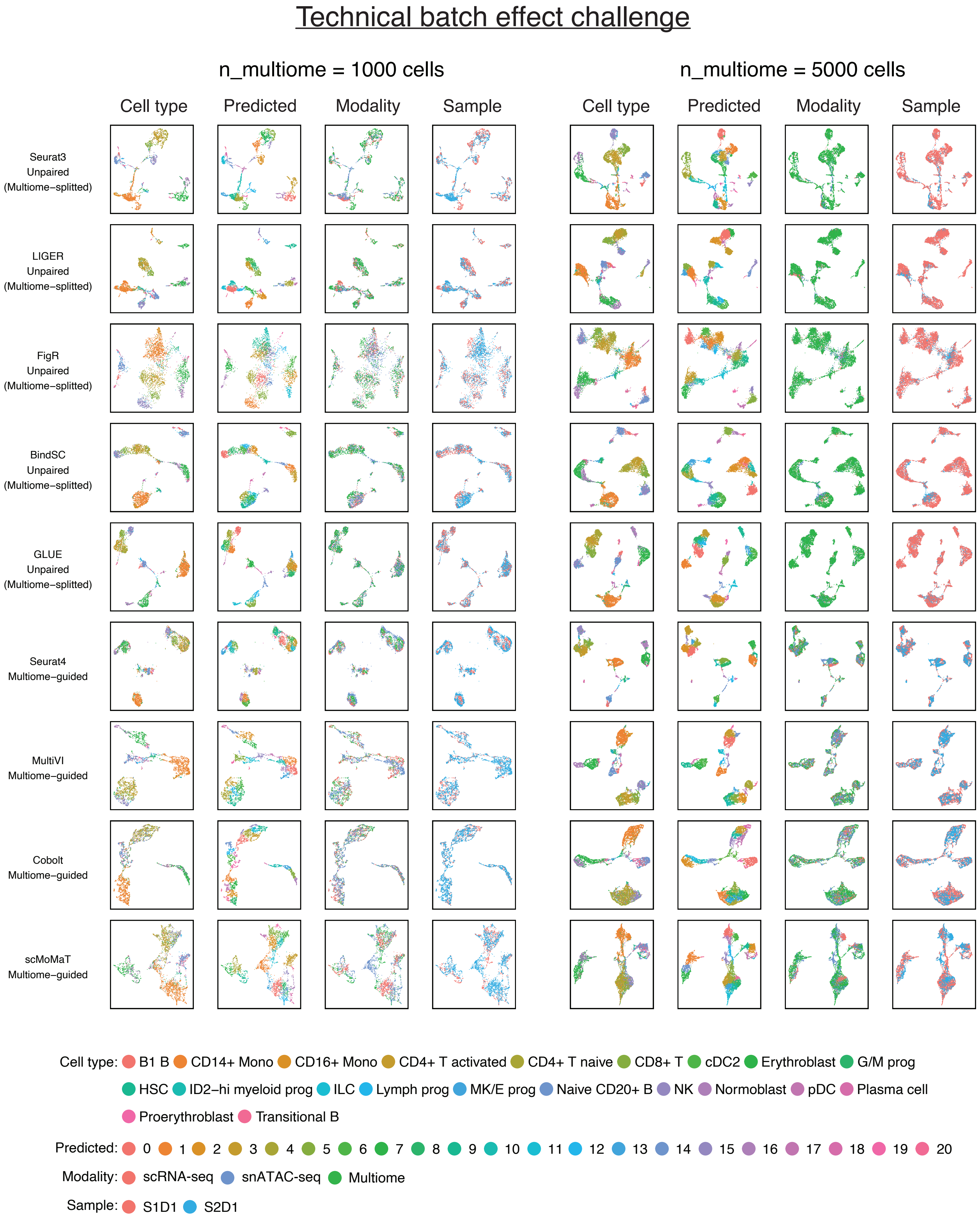

Fig. S13: UMAP plots for the BMMC-based simulations with technical batch effect challenge shown in Figure 5B (left).

Biological batch effect challenge

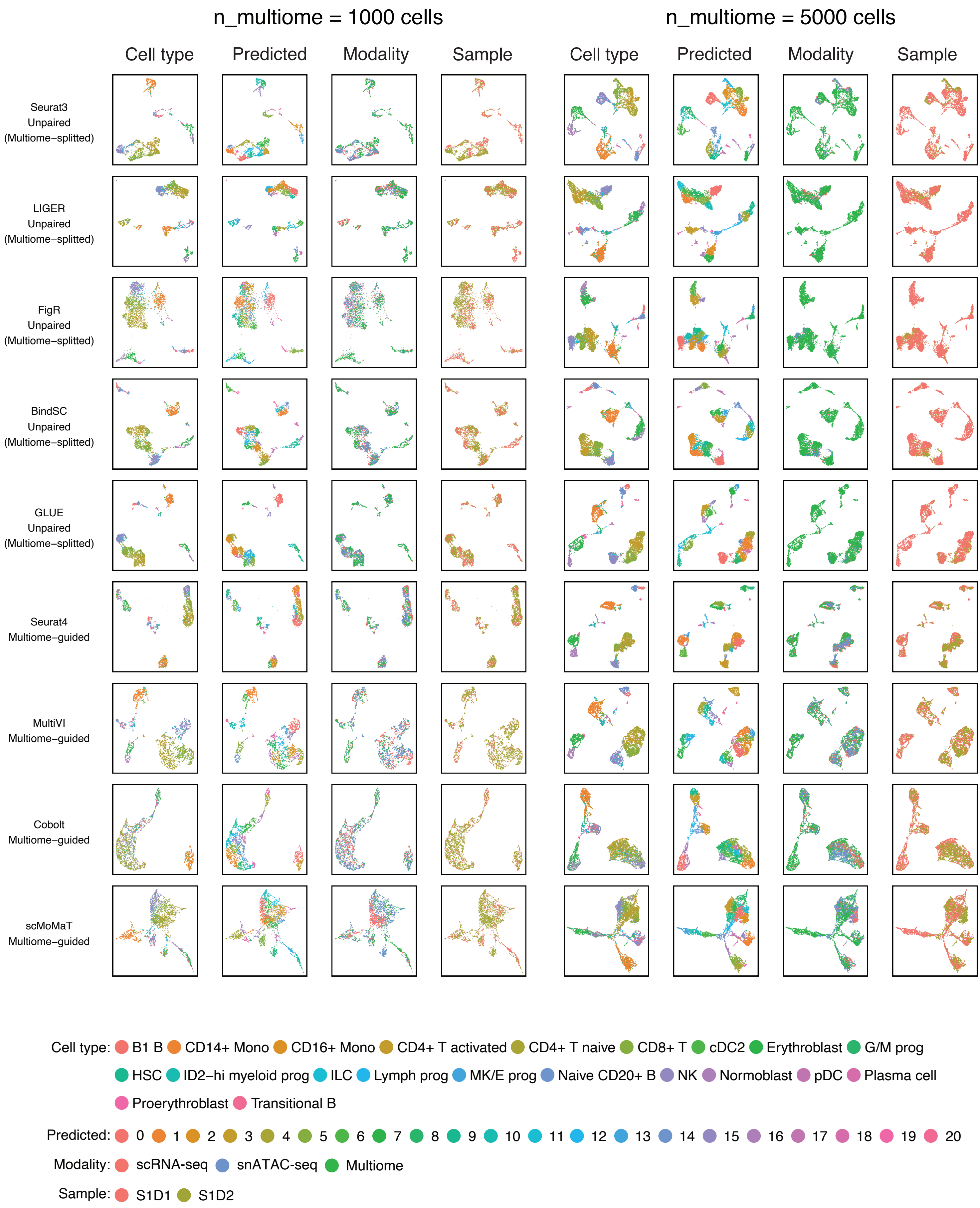

Fig. S14: UMAP plots for the BMMC-based simulations with biological batch effect challenge shown in Figure 5B (right).

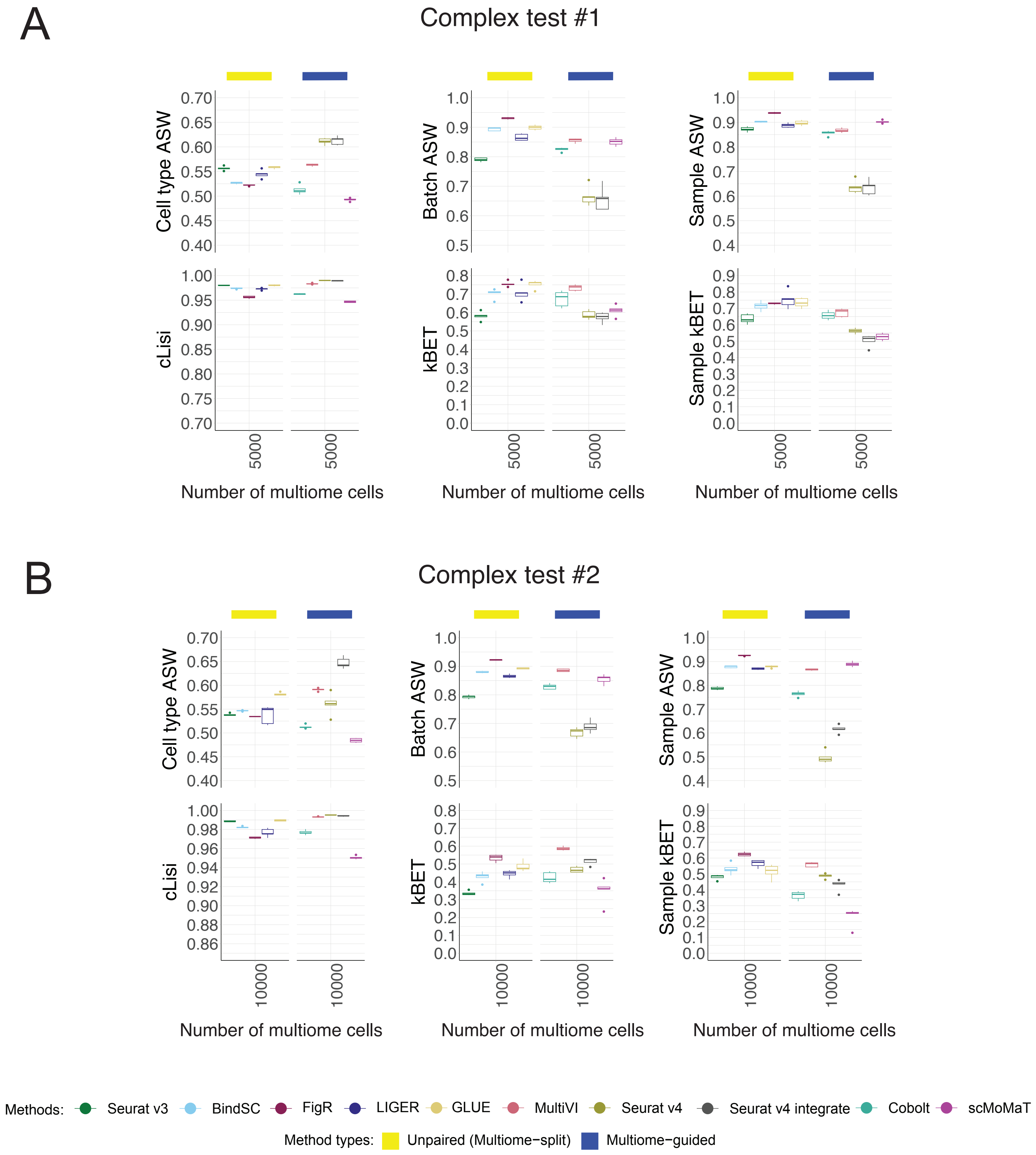

Fig. S15: Additional evaluation metrics for each method at integrating scRNA-seq, snATAC-seq and multiome data in the presence of more complex batch effects as described in Figure 4D. (A) Complex test #1 (Figure 5D left). (B) Complex test #2 (Figure 5D right). Cell type ASW and cLISI measure separation of cell types. Batch ASW and batch kBET measure the mixing of scRNA-seq, snATAC-seq, and multiome cells. Sample ASW and sample kBET measure the mixing of cells by sample ID (defined by site ID and donor ID). Runtime is measured in seconds, for each method, in log2 scale. Whisker is 1.5 times the inter-quartile range.

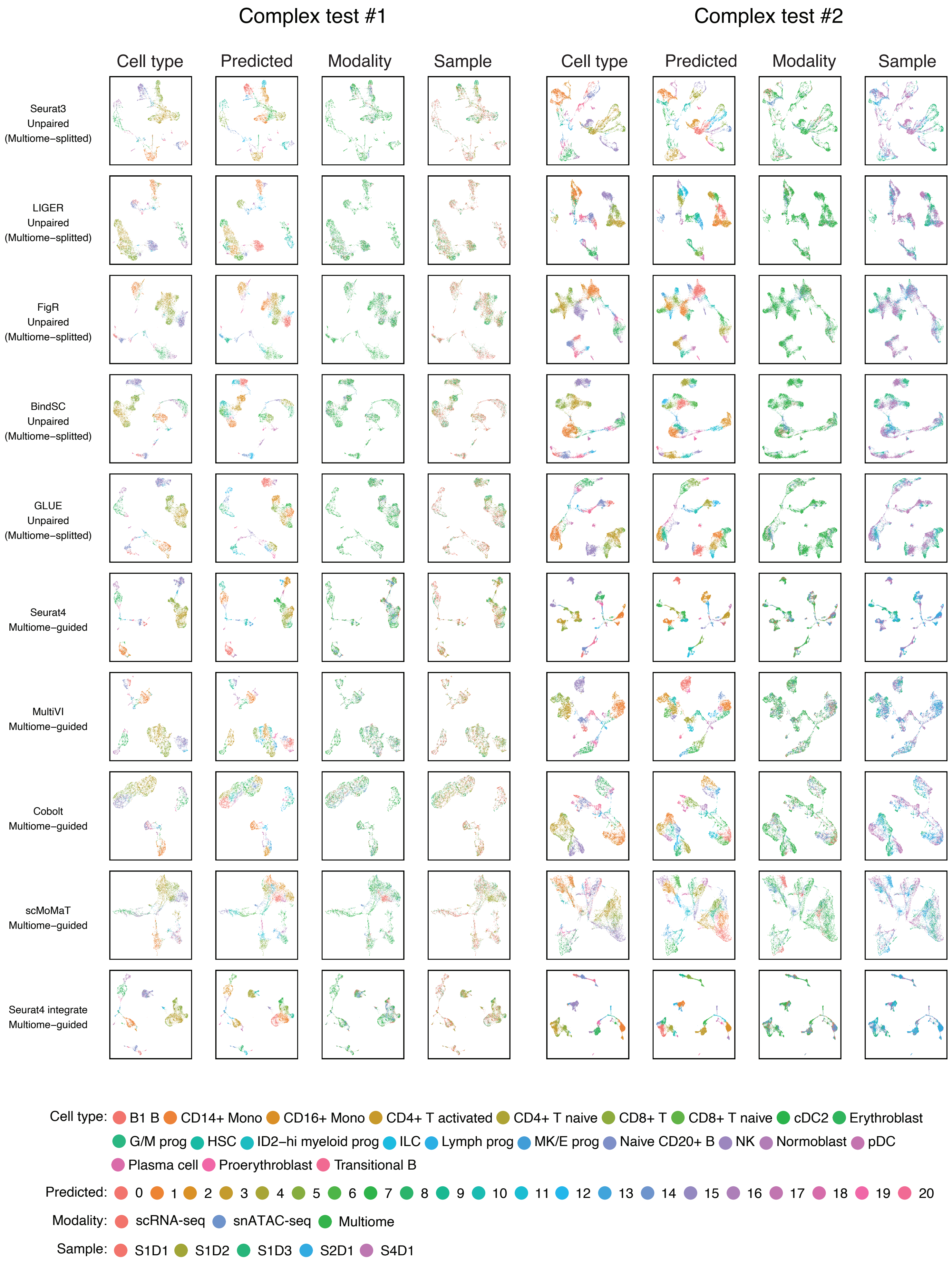

Fig. S16: UMAP plots for the BMMC-based simulations with complex batch effect challenge shown in Figure 5D.

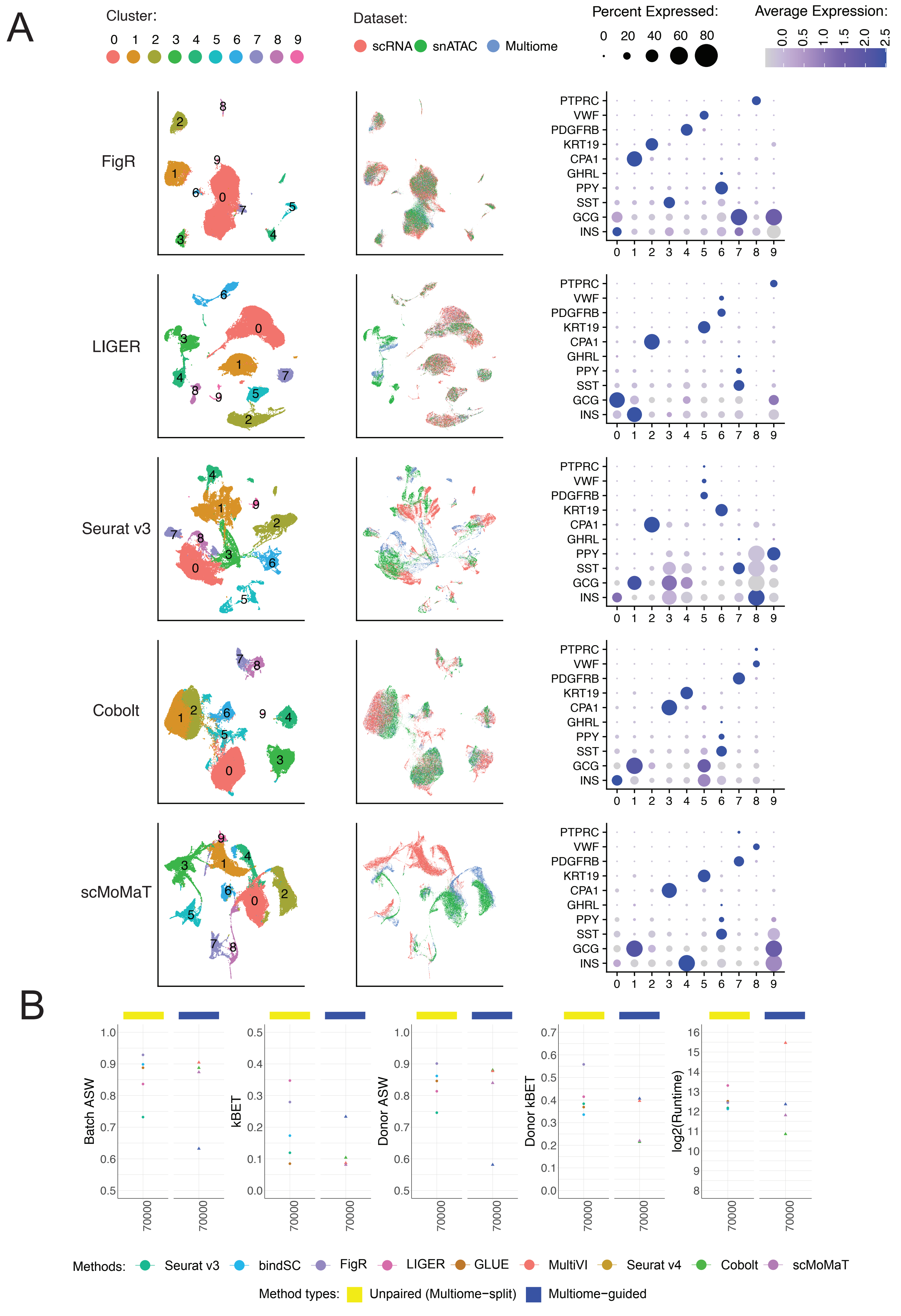

A
Cluster:
0 1 2 3 4 5 6 7 8 9
Dataset:
scRNA
snATAC
Multiome
Percent Expressed:
0 20 40 60 80
Average Expression:
0.0 0.5 1.0 1.5 2.0 2.5
FigR
LIGER
Seurat v3
Cobolt
scMoMaT
PTPRC
VWF
PDGFRB
KRT19
CPA1
GHRL
PPY
SST
GCG
INS
B
Batch ASW
kBET
Donor ASW
Donor kBET
log2(Runtime)
70000
Methods:
Seurat v3
bindSC
FigR
LIGER
GLUE
MultiVI
Seurat v4
Cobolt
scMoMaT
Method types:
Unpaired (Multiome−split)
Multiome−guided

Fig. S17: Additional integration results for the Human Pancreas Analysis Program (HPAP) datasets as described in Figure6. (A) Integration results of additional methods: UMAP projection using integrated embedding for a select number of methods, colored by cluster ID (left), data type (middle), and dot-plots showing the expression of marker genes per cluster, colored by average expression, sized by the percentage of cells expressing the gene
(right). B) Additional evaluation metrics for each method at integrating scRNA-seq, snATAC-seq and multiome datasets. Batch ASW and batch kBET measure the mixing of scRNA-seq, snATAC-seq, and multiome cells. Donor ASW and donor kBET measure the mixing of cells by donor ID. Runtime is measured in seconds, for each method, in log2 scale.

Supplementary methods

We evaluated a total of nine methods. Each method was run according to the most relevant tutorial available. The source code and processed data used for simulations are available on GitHub at https://github.com/myylee/benchmark_sc_multiomic_integration.

Multiome-guided integration methods

Seurat v4 [1]

Each modality in the multiome dataset was processed with a standard approach. Specifically, RNA-seq profile was normalized with scTransform and reduced to 50 dimensions using principal component analysis (PCA). ATAC-seq profile was processed with the latent semantic indexing (LSI). Specifically, the cell-by-peak matrix was normalized with term frequency-inverse document frequency (TF-IDF) and reduced to 50 dimensions using singular vector decomposition (SVD). The two separate latent embeddings were joined through the weighted-nearest neighbor (WNN) approach, using the top 50 principal components for the RNA-seq and the top 2-50 component for ATAC-seq, excluding the first component mainly correlated with sequencing depth. Single modality datasets were processed using the same standard workflow. Then, the raw data matrix was projected to the WNN-integrated space using supervised PCA and supervised LSI. Normalized gene expression values were imputed using anchor-based approximation for the ATAC-seq cells. The multiome analysis was done https://satijalab.org/seurat/articles/weighted_nearest_neighbor_analysis.html#wnn-analysis-of-10x-multiome-rna-atac-1. ScRNA-seq was mapped following https://satijalab.org/seurat/articles/multimodal_reference_mapping.html#example-1-mapping-human-peripheral-blood-cells-1. We then adopted this framework to project the snATAC-seq dataset to the multiome reference.

Seurat v4 integrate

For the last two simulations described in Figure 4, the multiome dataset was composed of samples from multiple donors exhibiting batch effects. Before learning a joint representation for the cells using WNN, a within-modality integration was employed to

mitigate batch effects. The RNA-seq profiles were integrated using canonical correlation analysis (CCA) described in https://satijalab.org/seurat/articles/sctransform_v2_vignette.html#perform-normalization-and-dimensionality-reduction-1. The ATAC-seq cells were integrated following steps described in https://stuartlab.org/signac/articles/integrate_atac.html. Then, the same WNN workflow and the supervised mapping of single-modality datasets were performed to project single-modality datasets to the batch-corrected space.

MultiVI [2]

MultiVI trains a variational autoencoder to learn a latent representation for cells in all three data types. Firstly, the two modalities of the paired datasets were horizontally stacked. Then, the paired and unpaired datasets were combined, with a modality column indicating which technology the cell belongs to. Features appearing in fewer than 1% of cells were filtered out. Then, the MultiVI model was trained with default parameters. Specifically, an autoencoder was trained for each modality using both the single-modality dataset and multi-modal dataset. Then, a symmetric Kullback-Leibler (KL) divergence loss was used to align the RNA-seq latent embedding and the ATAC-seq latent embedding of the paired cells. The model was trained using default parameters as described in https://docs.scvi-tools.org/en/stable/tutorials/notebooks/MultiVI_tutorial.html. The latent state of each cell was extracted using the converged model to represent the integrated cell state. The unmeasured RNA-seq profile of the unpaired ATAC-seq cells were inferred by passing the latent embedding through the learned decoder. When integrating datasets with additional batch labels, such as donor ID or site ID, these were included as covariates in the model.

Cobolt [3]

Cobolt also learns a latent representation of the cells using a variational autoencoder structure. Specifically, a modality-specific neural network was trained for each modality, using both unpaired and paired cells. For the paired cells, the latent representation of its

ATAC-seq profile and RNA-seq profile were multiplied to jointly define a cell's identity. A projection from the single modality latent embedding to the multi-modal latent representation was trained using the paired cells and later applied to the single-modality cells to ensure both unpaired and paired cells reside on the same space. Cobolt was run as described in the tutorial https://github.com/epurdom/cobolt/blob/master/docs/tutorial.ipynb.

scMoMaT [4]

scMoMaT uses a matrix tri-factorization model to deconvolute each cell-by-feature into a cell embedding factor (Ci), a feature factor (Cj), and an association matrix between cell i and feature j. For datasets simulated with PBMC, BMMC, or SHARE-seq mouse skin dataset as the source, we first selected 5000 highly variable genes for scRNA-seq and the RNA-seq profile of the multiome dataset separately. Then, we found the intersection of the two and used that as the final highly variable genes. For integration of the HPAP datasets, highly variable genes were selected using only the multiome datasets. RNA-seq profiles were normalized with quantile normalization.

Regarding ATAC-seq profiles, peak counts were first binarized. Then, a pseudo-counts matrix was calculated for snATAC-seq datasets. Essentially, for each gene, accessible ATAC peaks that overlap with the gene body or 2kb upstream of its transcriptional start site are summed up, and then binarized, to represent the pseudo-count of the gene. As a result, a list of RNA matrices and a list of ATAC-seq matrices were passed into the scMoMaT model. A scRNA-seq dataset is represented by just a normalized cell-by-gene matrix. A snATAC-seq dataset is represented by a binarized cell-by-peak matrix and a binarized cell-by-gene pseudo-counts matrix. Multiome datasets are represented by a normalized cell-by-gene matrix and a binarized cell-by-peak matrix. The scMoMaT model was trained using lambda=0.001, batch size=0.1, seed=0, K=30, interval=1000, T=4000, and learning rate=0.001. After training, cell factors are extracted to represent cells in the integrated space. We tried post-processing steps described in the original publication [4] for some of the simulated data, but no improvement on clustering result was observed. Thus, we did not include this step in our standard pipeline. ScMoMaT

was run following the steps described in this tutorial https://github.com/PeterZZQ/scMoMaT/blob/main/test/test_mop_5batches.py.

Unpaired integration methods

Liger [5]

This method can integrate unpaired scRNA-seq and snATAC-seq datasets. The peak-count matrix was first converted into a gene activity matrix, aggregating reads mapped within 2-kb upstream or downstream of the transcription start site (TSS). Both ATAC-seq and RNA-seq profiles were normalized by total expression across each cell. Highly variable genes were selected using the second dataset, the RNA-seq profiles, using default parameters that select genes with variance greater than 0.1. Then the scaleNotCenter was used to scale the normalized data matrices. Joint matrix factorization was performed using optimizeALS with k = 20. Quantile normalization was performed on the resulting cell loading across RNA-seq and ATAC-seq profiles, allowing the datasets from two modalities to be integrated. Louvain clustering was then used with the goal to generate a specific number of clusters. UMAP loading was calculated using cosine distance between quantile normalized cell loadings, with 30 neighbors and a minimum distance of 0.3. We followed the tutorial at http://htmlpreview.github.io/?https://github.com/welch-lab/liger/blob/master/vignettes/Integrating_scRNA_and_scATAC_data.html.

Seurat v3 [6]

Seurat v3 integrates unpaired RNA and ATAC datasets using the canonical correlation analysis (CCA). RNA-seq was normalized by library size and log-transformed. Top 2,000 highly variable genes were identified and used for dimensional reduction with PCA. ScATAC-seq data was converted to gene activity matrix as described above and normalized in the same way as the RNA-seq data. Using the highly variable genes identified in the gene expression data as the features, anchors between RNA-seq and ATAC-seq were identified, and the two profiles were aligned using CCA. Canonical aligned components were used as integrated cellular loadings. Normalized gene

expression was imputed by merging the expression of neighboring RNA cells. We followed steps from https://satijalab.org/seurat/articles/atacseq_integration_vignette.html.

FigR [7]

FigR projects scRNA-seq and snATAC-seq to one shared latent embedding using a similar workflow as Seurat v3. In addition, it computationally pairs cells from different modalities, creating a pseudo-multiome dataset for downstream analyses such as peak-gene pair identification. Specifically, the cell-peak count matrix was converted to gene activity matrix and normalized in the same way as the gene expression matrix. The top 5,000 most variable genes were identified using the gene expression matrix and the gene activity matrix individually, and the union of the two lists was obtained as the feature for downstream analysis. Canonical correlation analysis (CCA) was applied to align the ATAC and RNA profiles. L2 normalization was applied to the top 30 CCA components. OptMatch algorithms were used to pair the RNA and ATAC cells, using the 30 CCA component are latent embedding. Cells paired with multiple cells were removed, resulting in a 1-to-1 cell pairing. We implemented the preprocessing and CCA alignment of the unpaired datasets using functions in the Seurat package, and we used the OptMatch from the FigR GitHub page [7].

BindSC [8]

BindSC integrates unpaired single-cell datasets by estimating a cell-by-gene matrix Z for the snATAC-seq cells that maximizes the correlation between Z and the cell-by-peak matrix (Y) as well as between Z and the scRNA-seq data (X). The simultaneous similarity maximization is done in the latent embedding space through the bi-direction CCA. Firstly, 5,000 highly variable genes were selected using the RNA-seq profile. Then, the ATAC-seq profile was converted into the gene activity matrix as an initial approximation of the transformed matrix, Z. BiCCA was run with using default setting with lambda=0.5 and alpha=0.5, K=15, num.iteration=100, block.size=0. Through this process, Z was iteratively improved to better approximate the gene expression for the snATAC-seq cells while maintaining its similarity with the ATAC-seq profile. After

training, a latent representation of the scRNA-seq and snATAC-seq cells was learned and could be used for clustering. Moreover, the imputation of gene expression was performed using impuZ and fold-change normalization is performed to scale the imputed RNA expression. Only the 5,000 highly variable genes used for integration were imputed. We followed the steps from https://htmlpreview.github.io/?https://github.com/KChen-lab/bindSC/blob/master/vignettes/mouse_retina/retina.html.

GLUE [9]

GLUE integrates unpaired single-cell datasets by building a modality-specific autoencoder to extract a low dimensional embedding per modality, while using a knowledge-based (‘guidance’) graph to learn relationship between RNA and ATAC features. GLUE first requires the preprocessing of scRNA-seq and snATAC-seq datasets separately. Standard preprocessing steps for scRNA-seq dataset include filtering genes expressed by less than 3 cells, identifying top 2,000 highly variable genes, normalize by library size, log1p transformation, z-score standardization and PCA reduction (100 components). For snATAC-seq, peaks accessible in less than 1 cells are removed and the LSI dimensional reduction was performed to reduce the data into 100 dimensions, using the scglue.data.lsi function. A guidance graph was built using hg38 genome-build for human datasets and mm10 genome-build for mouse datasets. Regarding the guidance graph, an ATAC peak is considered to be connected to a gene if it overlaps in either the body of the gene or promoter region, which is defined as 2kb upstream of the transcriptional start site of the gene. Lastly, the GLUE model is fit and an integrated latent representation (50 components) is extracted for each cell. We followed the steps from https://scglue.readthedocs.io/en/latest/tutorials.html. When integrating datasets with additional batch labels, such as donor ID or site ID, these were included as covariates in the model by setting the batch parameter in scglue.models.configure_dataset.

Integration of HPAP dataset

Some unpaired integration methods do not have clear instruction for dealing with batch effects across samples. HPAP samples show large batch effects as these are cells from different human donors. Therefore, for methods that perform z-score standardization (Seurat v3, BindSC, FigR), we tried to correct for some batch effects by performing library-size and z-score standardization per sample, and then aggregate the data across donors. Moreover, to select for highly variable genes that could represent all samples equally, we first ran 10,000 highly variable gene selection for every sample, and the 5,000 high variable genes ranked the highest across samples were selected.

For LIGER, instead of providing two counts matrix (one for snATAC-seq and one for scRNA-seq data), we treated each HPAP sample (defined by donor and technology) as one counts matrix, therefore, a total of 14 RNA-seq profiles (10 scRNA-seq and 4 multiome-RNA data), and 12 ATAC-seq profiles (8 snATAC-seq and 4 multiome-ATAC data) are the inputs to LIGER.

For GLUE and MultiVI, we provided sample ID as a batch covariate to the model. For scMoMaT, HPAP samples were processed as individual batches, so it knows that it is dealing with 22 samples. For Cobolt, we tried inputting each sample as individual datasets to the model, but results were worse than the original workflow. Thus, we ran the original workflow for this task. For Seurat v4, we ran the 'Seurat v4 integrate' pipeline as described above. This explicitly corrected for batch effects in the multiome dataset first, then mapped the single-modality datasets onto the reference. For more details, check the codes on our GitHub repository, https://github.com/myylee/benchmark_sc_multiomic_integration.

References

1. Hao Y, Hao S, Andersen-Nissen E, Mauck WM, 3rd, Zheng S, Butler A, Lee MJ, Wilk AJ, Darby C, Zager M, et al: **Integrated analysis of multimodal single-cell data.** *Cell* 2021, **184:**3573-3587 e3529.
2. Ashuach T, Gabitto MI, Koodli RV, Saldi GA, Jordan MI, Yosef N: **MultiVI: deep generative model for the integration of multimodal data.** *Nat Methods* 2023.
3. Gong B, Zhou Y, Purdom E: **Cobolt: integrative analysis of multimodal single-cell sequencing data.** *Genome Biol* 2021, **22:**351.
4. Zhang Z, Sun H, Mariappan R, Chen X, Chen X, Jain MS, Efremova M, Teichmann SA, Rajan V, Zhang X: **scMoMaT jointly performs single cell mosaic integration and multi-modal bio-marker detection.** *Nat Commun* 2023, **14:**384.
5. Liu J, Gao C, Sodicoff J, Kozareva V, Macosko EZ, Welch JD: **Jointly defining cell types from multiple single-cell datasets using LIGER.** *Nat Protoc* 2020, **15:**3632-3662.
6. Stuart T, Butler A, Hoffman P, Hafemeister C, Papalexi E, Mauck WM, 3rd, Hao Y, Stoeckius M, Smibert P, Satija R: **Comprehensive Integration of Single-Cell Data.** *Cell* 2019, **177:**1888-1902 e1821.
7. Kartha VK, Duarte FM, Hu Y, Ma S, Chew JG, Lareau CA, Earl A, Burkett ZD, Kohlway AS, Lebofsky R, Buenrostro JD: **Functional inference of gene regulation using single-cell multi-omics.** *Cell Genom* 2022, **2**.
8. Dou J, Liang S, Mohanty V, Miao Q, Huang Y, Liang Q, Cheng X, Kim S, Choi J, Li Y, et al: **Bi-order multimodal integration of single-cell data.** *Genome Biol* 2022, **23:**112.
9. Cao ZJ, Gao G: **Multi-omics single-cell data integration and regulatory inference with graph-linked embedding.** *Nat Biotechnol* 2022, **40:**1458-1466.
