# Supplementary material for: Benchmarking algorithms for joint integration of unpaired and paired single-cell RNA-seq and ATAC-seq data
Source: Genome Biol. 2023 Oct 24;24:244. doi: 10.1186/s13059-023-03073-x (PMC10594700; doi:10.1186/s13059-023-03073-x)
Supplement: Supplementary file 2 — Additional file 2. Review history. [file 13059_2023_3073_MOESM2_ESM.docx]

Review History

First round of review

Reviewer 1

Were you able to assess all statistics in the manuscript, including the appropriateness of statistical ts used?

Yes, and I have assessed the statistics in my report.

Comments to author:

This paper presents the benchmark of single-cell multi-omics integration methods. However, there are several points that need to be fixed in my opinion. My comments are as follows:

1. I think there are some other integration methods the paper is missing, such as GLUE (https://www.nature.com/articles/s41587-022-01284-4). The authors need to do a more comprehensive literature search to include all relevant methods.

2. Some other integration possibilities are to predict the genome-wide chromatin accessibility using gene expression and to integrate the predicted and actual chromatin accessibility. These methods include BIRD (https://academic.oup.com/nar/article/47/19/e121/5552071) and ChromDragoNN (https://www.ncbi.nlm.nih.gov/pmc/articles/PMC6612838/). Consider adding these methods into comparison.

3. The PBMC simulation datasets depend on computational cell type annotation based on Seurat pipeline, which is not convincing enough to serve as gold standard. For example, there are clearly two subsets of b cells and DC. Will the results change if they are split into two groups? Also will using Seurat pipeline introduce bias since Seurat itself is a method being compared to (although different parts are used)? What happens if other computational pipelines like scanpy are used? If the results depend on clustering resolution or computational pipeline, the results may not be convincing.

4. The BMMC simulation dataset actually represents three differentiation trajectories. I wonder if using discrete cell types as the gold standard is appropriate since some computationally defined cell types are actually intermediate cell states between cell types which may not be homogeneous. How about using trajectories to evaluate the results?

5. Cell types defined in PBMC and BMMC are only inferred using scRNA-seq. Why not using snATAC-seq to define cell types or jointly use scRNA and snATAC? This is related to my previous concern that the gold standard may not be good enough.

6. For the 'peak to gene pair' analysis, is it also possible to evaluate the imputation of chromatin accessibility using gene expression using the methods mentioned in point 1?

7. The authors mentioned that the peak gene pair analysis suffers from the high sparsity of the data? How about using imputation methods for scRNA-seq such as SAVER and MAGIC and for snATAC-seq such as SCALE and SCATE?

8. Benchmarking on only two simulation studies may not be convincing enough. Consider benchmarking on at least one real dataset.

9. The running time experiment needs to include cases with a much larger number of cells. 8000 cells is not a large number given the scale of datasets now and in the future. Running time for datasets with 1 million cells will be desirable.

10. The plots need to be improved. They are too monotonous right now. The scale of the y-axis also need to be adjusted to enlarge the actual plotting part. For example Figure 4D NMI the y-axis can be rescaled to (0.5,0.8).

Reviewer 2

Were you able to assess all statistics in the manuscript, including the appropriateness of statistical tests used?

Yes, and I have assessed the statistics in my report.

Comments to author:

Integrating data from different modalities is an important problem in the analysis of single-cell genomic data. In this manuscript, the authors benchmarked seven methods for integrating single-cell RNA-seq (scRNA-seq) and single-cell ATAC-seq (scATAC-seq) data using two datasets with different complexity levels (PBMC and BMMC). They found that single-cell multiome data are helpful for annotating cell types in single modality data. The cell number needs to be large enough for multiome-guided integration to be effective. With fixed cost, increasing cell number is more important than increasing sequence depth. These findings provide a number of useful guidelines for designing future single-cell multiome studies. Therefore, it is worthwhile to have them documented in the literature. I have several questions and comments listed below. I hope that they can help the authors to improve their study and make the conclusions more robust and broadly applicable.

Major issues:

1. It may be useful to evaluate performance and robustness of different methods when the cell types in single-modality scRNA-seq and scATAC-seq are not exactly the same. Will the addition of multiome data help in this case? How will the integration performance depend on cell number of the multiome data? You may simulate data with different degrees of cell type overlap between the two data modalities for this evaluation.

2. Additionally, it will also be useful to evaluate performance and robustness of different methods when the cell types in multiome data and the cell types in single modality data are not exactly the same. You may simulate data with different degrees of cell type overlap between the multiome and single modality data for this evaluation.

3. Another question of interest is that when multiome data are already available, is it still useful to use previously generated single modality data? To answer this question, it will be helpful to compare the unpaired, unpaired (multiome split), and multiome-guided integration methods to analysis results one would obtain using only the multiome data without any single modality data.

4. Line 243 of the manuscript: "Seurat v3 revealed a higher percentage of recovered peak-gene pairs and better F1 score than the measured paired gene expression and chromatin accessibility profile from 1000 cells". The higher percentage of recovered peak-gene pairs is true in Fig 2B, but the better F1 score is not supported by Fig 2B. Indeed, Fig 2B shows that Seurat v3 has a lower F1 score compared to paired data from 1000 cells.

5. Line 246-247: "However, we also note that the predicted profiles only recovered less than 45% of the ground truth list … It is only recovering a small percentage of peak-gene pairs revealed by the experimentally generated multiome dataset". This statement is somewhat misleading. The peak-gene list from unpaired data was obtained using only 1000 cells per data modality, but the ground truth list was obtained using over 10,000 cells. The small percentage (45%) here cannot be used to conclude that imputation-based peak-gene list can only recover a small percentage of true peak-gene list. A fair comparison should be based on the same cell number. For example, if one would have 10,000 single-modality cells and their imputed values, what percentage of ground truth peak-gene list would be recovered where the ground truth is obtained using 10,000 multiome cells.

6. Only two datasets (PBMC, BMMC) are used for this evaluation. Among them, simulations based on the PBMC dataset do not have batch effect between the single-modality and multiome cells, and the "ground truth" cell type label of this dataset is computationally inferred using Seuratv4 (which is also a method to be evaluated) rather than experimentally determined. Thus, many evaluations (e.g. batch effects, cell type annotation) can only be rigorously done using one benchmark dataset (BMMC). If it is possible to compile more benchmark datasets, it would be very useful to see whether the main findings remain consistent across two or more benchmark datasets. If so, the conclusions would be more robust.

Minor issues:

7. The first sentence in the Background section (i.e., line 37-39 of the manuscript): "Over the past ten years, hundreds of single-cell RNA-seq … or single-nucleus ATAC-seq … [datasets] have been produced by laboratories worldwide". The word "[datasets]" is missing.

8. Line 154: "We evaluated four popular unpaired integration methods (Seurat v4, …)". Here Seurat v4 should be Seurat v3.

9. Line 346: (Figure 3D) should be (Figure 3E)est

Authors Response

Point-by-point responses to the reviewers’ comments:

*Response: We would like to express our sincere gratitude to the reviewers for their valuable comments, which have greatly contributed to the improvement of our manuscript. Based on the feedback provided, we have made substantial revisions to our work, and have highlighted the revised and new sections in red for ease of reviewing. Below, we have provided a point-by-point response to the reviewers’ comments. The original reviewers’ comments are in bold italics and our responses are in normal font colored in blue. The major changes that we made in this revision include the following: 1. Added two methods to the evaluations: GLUE (Cao and Gao Nature Biotech 2022, PMCID: PMC9546775) and scMoMaT (Zhang et al. Nature Comm 2023, PMCID: PMC9873790). 2. Evaluated two additional datasets: a multimodal dataset with ground-truth cell type labels from Ma et al. (Cell 2020, PMCID: PMC7669735) for benchmark evaluation, and the scRNA-seq, snATAC-seq, and multiome datasets from the Human Pancreas Analysis Program (https://hpap.pmacs.upenn.edu) to represent a real data scenario. 3. Added simulations where the single-modality datasets have different cell type overlaps, as well as situations where the multiome dataset has different cell type overlap with the single-modality datasets.*

Reviewer #1:

This paper presents the benchmark of single-cell multi-omics integration methods. However, there are several points that need to be fixed in my opinion. My comments are as follows:

1. I think there are some other integration methods the paper is missing, such as GLUE (https://www.nature.com/articles/s41587-022-01284-4). The authors need to do a more comprehensive literature search to include all relevant methods.

*Response: Thank you for the constructive feedback. To ensure the inclusion of all relevant methods, we did a comprehensive literature search. Specifically, we checked all methods listed in three review articles on the topic of single-cell multi-omic data integration methods (Miao et al., Nature Reviews Nephrology 2021, PMCID: PMC9191639; Adossa et al., Computational and Structural Biotechnology Journal 2021, PMCID: PMC8114078; Argelaguet et al., Nature Biotechnology 2021, PMID: 33941931). In addition, we did PubMed and Google search using keyword ‘single-cell multiomic integration’ (as of 3/28/2023). This resulted in 18 methods that were not included in the original submission. However, after careful reading of these papers, we found that many of them did not fall in the scope of this benchmarking manuscript, and hence were excluded from further evaluations.*

*The table below summarizes all relevant methods, which are categorized into five groups:*

*• ‘Unpaired integration’: integrating two datasets with measurement of different modalities and different cells.*

*• ‘Multiome-guided integration’: integration of paired dataset, and two unpaired datasets, using the paired dataset as a guide.*

*• ‘Mosaic integration’: integration of any number of datasets, can be paired or unpaired, of two or more types of features.*

*• ‘Paired integration’: integrating measurement of different modalities of the same cells.*

*• ‘Others’: other types of integration not mentioned above.*

*If a method belongs to the ‘Unpaired integration’ category, we check if there’s instruction on how to apply this method for the integration of scRNA-seq and snATAC-seq datasets.*

*If a method belongs to the ‘Multiome-guided integration’ or ‘Mosaic integration’ category, we check if there’s instruction on how to apply this method for the integration of scRNA-seq, snATAC-seq, and multiome datasets.*

*The main goal of this benchmark evaluation paper is to assess the ability of different methods in integrating both paired and unpaired single-cell multi-modal data. As such, we include a method if it meets the following criteria:*

*1) It is able to integrate unpaired scRNA-seq and snATAC-seq datasets (methods in the ‘unpaired integration’ category) and have instructions demonstrating how the integration is performed. We do not include paired integration method because it has the assumption that the two modalities are measured in the same cells, thus not applicable to unpaired scRNA-seq and snATAC-seq datasets.*

*2) It is able to integrate scRNA-seq, snATAC-seq, and multiome datasets (methods in the ‘Multiome-guided integration’ or ‘Mosaic integration’ category) and have instructions demonstrating how the integration is performed.*

*Among the 18 methods, we added GLUE (Cao and Gao, Nature Biotech 2022, PMCID: PMC9546775) and scMoMaT (Zhang et al., Nature Communications 2023, PMCID: PMC9873790) to our evaluations. GLUE is a recently published unpaired-integration method that demonstrated superior performance when integrating unpaired scRNA-seq and snATAC-seq datasets. ScMoMaT is also a recently published method that belongs to the category of mosaic integration methods, capable of integrating datasets that share only cells, only features, or none of the two. These situations are the most complex cases of the single-cell multi-modal integration problem, and the situations our manuscript focuses on are only a subset of the mosaic integration problems. We chose to include scMoMaT to represent the mosaic integration methods due to its superior performance compared to other published methods in this category. With the addition of GLUE and scMoMaT, we now have 9 methods in our evaluations.*

2. Some other integration possibilities are to predict the genome-wide chromatin accessibility using gene expression and to integrate the predicted and actual chromatin accessibility. These methods include BIRD (https://academic.oup.com/nar/article/47/19/e121/5552071) and ChromDragoNN (https://www.ncbi.nlm.nih.gov/pmc/articles/PMC6612838/). Consider adding these methods into comparison.

*Response: Thank you for the suggestion and we agree that imputing genome-wide chromatin accessibility is an important problem. However, the main goal of our paper is to evaluate the ability of different methods in integrating paired and unpaired scRNA-seq and snATAC-seq data rather than imputing missing modalities. While the missing-modality prediction performed by the applicable methods in our current evaluations serves as an intermediate step towards predicting peak-gene pairs, we didn’t specifically focus on missing modality imputation due to the lack of consensus on how to quantify chromatin accessibility in snATAC-seq data. Unlike scRNA-seq where the counts estimate numbers of molecules, there is not a common agreement on what biological state is being estimated from snATAC-seq data. Existing snATAC-seq analysis methods create chromosomal domain features either by arbitrarily dividing the entire genome into fixed-width segments (features usually referred to as bins) or estimating discrete domains by peak-calling from aggregated pseudo-bulk data (features usually referred to as peaks). As such, it is challenging to evaluate the performance of different methods for chromatin accessibility prediction and we feel adding these methods in comparison is beyond the scope of this manuscript.*

3. The PBMC simulation datasets depend on computational cell type annotation based on Seurat pipeline, which is not convincing enough to serve as gold standard. For example, there are clearly two subsets of b cells and DC. Will the results change if they are split into two groups? Also will using Seurat pipeline introduce bias since Seurat itself is a method being compared to (although different parts are used)? What happens if other computational pipelines like scanpy are used? If the results depend on clustering resolution or computational pipeline, the results may not be convincing.

*Response: We agree that PBMC dataset is not the most optimal dataset as its annotation was obtained using the Seurat pipeline, which biased the results towards Seurat v4.*

*Regarding the question of whether a different number of clusters will change the result, we believe this is not the case. All methods were run to obtain the same number of clusters, and this is achieved by finding a resolution parameter that returns the desired number of clusters in every run. By calculating resolution value in this data-dependent manner per replicate, we believe the results would not be dependent on specific hyperparameter values.*

*We appreciate your suggestion on using Scanpy. As far as we know, Scanpy unfortunately has not implemented in the Seurat v4 algorithm, which annotates cells using both gene expression and chromatin accessibility profiles. Nevertheless, we agree that there is a bias towards Seurat v4 for PBMCsimulated situations. Therefore, we did another comprehensive literature search for additional multimodal datasets that can be used as ground-truth datasets. In the end, we only found one applicable dataset, a mouse-skin dataset generated by SHARE-seq (Ma et al., Cell 2019). Below is the description of the SHARE-seq mouse skin dataset (lines 161-168 in the revised manuscript).*

*The third multiome dataset [1] employed a distinct technology called SHARE-seq, developed by the Buenrostro group, to profile cells from the mouse skin. This dataset offers joint profiles of 32,231 cells, which have been annotated into 22 different cell types (Supplementary Figure 5). It encompasses cells from various lineages, including regenerative compartments of the hair follicles, as well as distinct cell groups like endothelial and macrophages[1]. Although the sequencing depth of this dataset is slightly lower than the two previous multiome datasets, it has the largest number of cells. Table 1 provides detailed information about each dataset.*

*Using this additional dataset, we repeated the same simulations as described in Figure 2, testing the integration result of sinlge-modality datasets with versus without the inclusion of multiome dataset. Below is the description of our evaluation results (lines 319-339 of the revised manuscript.)*

*SHARE-seq Mouse Skin dataset We conducted a similar study using the SHARE-seq mouse skin dataset to validate our findings. Given the larger number of cells available in this dataset, we designed a simulation with 8,000 scRNA-seq cells and 8,000 snATAC-seq cells. We then performed integrations without or with the inclusion of 5,000, 10,000, or 15,000 cells from the multiome dataset (Figure 3A). The integration performance of each method is depicted in Figure 3B. Consistent with the evaluation results from the PBMC- and BMMC-simulated scenarios, GLUE and Seurat v3 emerged as top-performing methods in the ‘unpaired (multiome-split)’ category, with GLUE being the best performer. Among the ‘multiome-guided’ methods, Seurat v4 exhibited the best performance and outperformed all methods in the two ‘unpaired integration’ categories. Regarding peak-gene pair recovery, Seurat v3 demonstrated the highest recovery in terms of the absolute number of peak-gene pairs, surpassing the value obtained from the truly paired profiles. However, when considering the F1 score, which corrects for false positive and false negative rates, FigR performed significantly better than the other methods and even outperformed the F1 score from the truly paired profiles. Seurat v4 achieved the highest F1 score among the multiome-guided methods and ranked second in overall performance, just behind FigR. In terms of runtime, GLUE had the longest execution time among the ‘unpaired integration’ category, while the remaining methods had similar runtime overall, comparable to that of Seurat v4. MultiVI had the longest runtime, whereas Cobolt and scMoMaT were the fastest (Figure 3C). Representative UMAP plots after integration are displayed in Figure 3D. UMAP plots of other integration methods are shown in Supplementary Figure 6.*

*We systematically searched for additional datasets and lastly only found the SHARE-seq mouse skin dataset shown above. Here, we describe our search process. We were looking for a multi-modal dataset that measures gene expression and chromatin accessibility at the same time. Furthermore, the cells need to be annotated based on both modalities, and the dataset needs to include at least 10k cells and a decent sequencing depth. Lastly, the cell type annotation should not be done with any method being evaluated in this manuscript, as that would bias the evaluation result.*

*Specifically, we searched on PubMed with these keywords: ((single-cell multiomics) OR (single-cell multiomics)) OR (single-cell gene expression and chromatin accessibility) and we looked through the top 20 hits that generated potentially usable datasets.*

*a. Profiled snRNA-seq and snATAC-seq, but not paired profiling, thus cannot be used as the groundtruth dataset for simulation:*

*a. Muto et al., Nature Commun. 2021, PMCID: PMC8044133.*

*b. Regner et al., Molecular Cell 2021, PMCID: PMC8642316.*

*c. Andueza et al., Cell Reports 2020, PMCID: PMC7801938.*

*d. Morabito et al., Nature Genetics 2021, PMCID: PMC8766217.*

*e. Long et al., Cell Discovery 2022, PMCID: PMC9296597.*

*f. Cao et al., Science 2020 (sci-RNA-seq3), PMCID: PMC7780123 and Domcke et al., Science 2020 (sci-ATAC-seq3), PMCID: PMC7785298.*

*g. Taavitsainen et al., Nature Communications 2021, PMCID: PMC8421417.*

*h. Chiou et al., Nature Genetics 2021, PMCID: PMC9037575 (snATAC-seq only).*

*b. Review articles:*

*a. Lee et al., Exp Mol Med. 2020, PMCID: PMC8080692.*

*b. Ma et al., Trends in Biotechnology 2020, PMCID: PMC7442857.*

*c. Yan et al., Genome Biology 2020, PMCID: PMC6996192 (for ATAC-seq analysis, not relevant in this context).*

*d. Preissl, Gaulton, and Ren, Nature Reviews Genetics 2022, PMCID: PMC9771884.*

*c. Other multi-modal technologies:*

*a. Mimitou et al., Nature Biotech 2021, PMCID: PMC8763625. This is the ASAP-seq dataset that allowed the joint profiling of chromatin accessibility and surface protein. Not eligible for the scope of this benchmarking study.*

*b. Guo et al., Cell Research 2017, PMCID: PMC5539349. Generated a dataset with single-cell COOL-seq technology which allows Joint profiling of chromatin accessibility, nucleosome positioning, DNA methylation. Not eligible for the scope of this benchmarking study.*

*d. Garcia-Alonso et al., Nature 2022, PMCID: PMC9300467. Single-Cell roadmap of human gonadal development.*

*a. scRNA-seq datasets are processed with scanpy, and then integrated with scVI. b. snATAC-seq cell type annotation was transferred from scRNA-seq, with CCA alignment approach.*

*e. Zhang et al., 2022 Cell Reports, PMCID: PMC8957708. Generated all three data types, but datasets are processed with Seurat/Signac for cell type identification. Thus, the study is biased towards one of methods being evaluated.*

*f. Argelaguet et al., Nature 2019, PMCID: PMC6924995. Joint profiling of chromatin accessibility, DNA methylation, and RNA expression using scNMT-seq. Only profiled 1,105 single cells, thus limited number of cells for this benchmarking study.*

*g. Chen, Lake, and Zhang, Nature Biotech 2019, PMCID: PMC6893138. SNARE-seq dataset only profiled 5,081 cells.*

*h. Ma et al., Cell 2020, PMCID: PMC7669735. This paper introduced a new multi-modal sequencing technology called SHARE-seq. The largest dataset from this paper was profiling cells from mouse skin development. A total of 34,774 cells were profiled, although the depth of the RNA-seq and ATAC-seq profiles was relatively low. The cells were first annotated using gene expression and chromatin accessibility independently and later merged to get a coherent annotation. The preprocessing of the gene expression profile was done using Seurat v3, but just the standard library size normalization and log transformation. The key method we evaluated in the Seurat v3 approach is the canonical correlation alignment (CCA) used to align the RNA-seq and ATAC-seq profiles, which 8 is not used by Ma et al. Thus, the annotation from this dataset does not bias towards any method. As a result, we added new simulations using this dataset as the ground-truth. There is a new paper by the same group released on 3/29/2023 (Hu et al., BioRxiv 2023, doi: 10.1101/2023.03.28.533945). The authors generated two additional large-scale datasets. We did not use this dataset as it has not been published yet, thus the cell type annotation may not be finalized. Nevertheless, we believe this could be a great source of datasets for future evaluations.*

*i. Open Problems in Single-Cell Analysis 2022 (link) generated multiome dataset from “mobilized peripheral CD34+ hematopoietic stem and progenitor cells (HSPCs) isolated from four healthy human donors”. However, the cell type annotation was preliminary result based on RNA gene expression only. Therefore, the cell type annotation label from this study should not be considered as a ground truth.*

4. The BMMC simulation dataset actually represents three differentiation trajectories. I wonder if using discrete cell types as the gold standard is appropriate since some computationally defined cell types are actually intermediate cell states between cell types which may not be homogeneous. How about using trajectories to evaluate the results?

*Response: Thank you for your insightful comment, and we appreciate your concerns regarding the use of discrete cell types as the gold standard in our evaluation. We acknowledge that the current discrete cell populations might include intermediate cell states and might not be fully homogeneous. However, it is important to note that the authors of the BMMC dataset did not provide a ground-truth trajectory label.*

*To obtain such a ground-truth trajectory label, additional methods would need to be employed, which could introduce biases favoring certain methods being evaluated. Furthermore, as we are not experts in hematopoiesis, we feel it would be inappropriate for us to make judgments regarding the correctness of inferred trajectories.*

*Lastly, it is worth noting that not all cells are involved in a single trajectory, and the BMMC dataset is actually consists of multiple differentiation trajectories. Given these considerations, we have decided not to utilize trajectories as an evaluation metric for the BMMC results.*

5. Cell types defined in PBMC and BMMC are only inferred using scRNA-seq. Why not using snATACseq to define cell types or jointly use scRNA and snATAC? This is related to my previous concern that the gold standard may not be good enough.

*Response: We apologize for the lack of clarity. The cell type annotations were defined using both RNA and ATAC profiles for both PBMC and BMMC datasets. For the PBMC dataset, it was annotated using the weighted-nearest neighbor approach which defines cell similarity using both gene expression and chromatin accessibility (Hao et al., Cell 2021, PMCID: PMC8238499). For the BMMC dataset, the annotation was first done in each modality separately and merged in an expert-supervised manner (see the screenshots below).*

6. For the ‘peak to gene pair’ analysis, is it also possible to evaluate the imputation of chromatin accessibility using gene expression using the methods mentioned in point 1?

*Response: We apologize for the lack of explanation in the original manuscript. We chose to evaluate peak-gene pair analysis using imputed gene expression and observed chromatin accessibility, instead of the other direction (imputed chromatin accessibility and observed gene expression) due to two reasons. Firstly, there was no standard in the field in terms of whether to represent chromatin accessibility data as binary variables (open or close), or quantitative values (number of reads mapped to the region). Two recent studies suggest a quantitative representation of the chromatin accessibility data is more desirable (Miao and Kim, bioRxiv 2022, doi: 10.1101/2022.04.20.488960; Martens et al., bioRxiv 2022, doi: 10.1101/2022.05.04.490536). This is the reason that we performed our peak-gene pair calculation using peak counts instead of binarized counts. However, not all methods model the chromatin accessibility data as quantitative measurements. MultiVI, for instance, binarizes the cell-peak counts matrix, treating each peak region as either open or closed. As a result, the imputation result from MultiVI is a list of probability measurements, representing the likelihood for each peak region to be accessible in each cell. Moreover, we tried using the predicted probability as input to the peak-gene pair calculation, but an unresolvable error persisted after repeated efforts (see screenshot below). Therefore, we were unable to use imputed chromatin accessibility data from MultiVI.*

*Furthermore, bindSC is not able to impute chromatin accessibility using a gene expression matrix. Thus, if we choose to calculate peak-gene pair identification using imputed chromatin accessibility, we would have one less method in the comparison. Lastly, there are often five-fold or ten-fold more features for chromatin accessibility data compared to gene expression data. Thus, the prediction task for chromatin accessibility is a much harder problem. Therefore, we reasoned that imputing gene expression from ATAC-seq is a more reliable approach.*

7. The authors mentioned that the peak gene pair analysis suffers from the high sparsity of the data? How about using imputation methods for scRNA-seq such as SAVER and MAGIC and for snATAC-seq such as SCALE and SCATE?

*Response: Thank you for the suggestion, and we appreciate your recognition that imputing dropouts with methods like SAVER, MAGIC, SCALE, and SCATE could potentially enhance the accuracy of peak-gene pair analysis. However, as mentioned previously, we have chosen to maintain the focus of our evaluations.*

*While scRNA-seq and snATAC-seq data are known to exhibit sparsity and noise, currently, there is no conclusive evidence suggesting that applying imputation to denoise the input consistently yields more biologically relevant results. Therefore, we remain cautious about applying these methods to our observed RNA-seq and ATAC-seq profiles before calculating the ground-truth list of peak-gene pairs.*

*Alternatively, we could apply imputation methods such as SAVER and MAGIC solely to predicted scRNAseq data to potentially reduce some sparsity. However, this approach introduces complexity that may hinder our interpretation. By employing denoising methods, it would become challenging to disentangle whether any changes in peak-gene pair recovery accuracy are solely attributed to the integration methods or influenced by the denoising algorithms employed.*

*The primary focus of calculating the peak-gene pair recovery accuracy is to evaluate the computationally paired cells as compared to experimentally generated ones. Thus, it is crucial that we direct our evaluation toward the performance of the in-silico integration methods themselves. Therefore, we have deliberately chosen to avoid additional preprocessing steps to better evaluate the impact of the integration methods on peak-gene pair analysis.*

8. Benchmarking on only two simulation studies may not be convincing enough. Consider benchmarking on at least one real dataset.

*Response: We completely agree with your comment. In this revision, we have added a real dataset generated from The Human Pancreas Analysis Program (HPAP). Given our expertise in analyzing human pancreatic islet single-cell datasets, we have a clear expectation of the results and are able to compare across integration methods. Below is our description of the HPAP datasets and the integration results (lines 444-495 in the revised manuscript).*

*Integration of single-cell multi-omic datasets from Human Pancreas Analysis Program (HPAP) Simulated datasets are critical for evaluating integration performance due to the availability of ground-truth cell type annotations. However, there are limitations with the use of simulated datasets. The three data types we simulated all came from the same single-cell multiome dataset and thus represent the most idealized situation where there is no batch effect between the singlemodality datasets and the multiome dataset. In reality, there are significant differences between datasets generated from different technologies, due to the different library preparation workflow. Moreover, the difference between scRNA-seq and snRNA-seq is non-negligible. To evaluate the performance of different integration methods in a more realistic situation, we used data generated by the Human Pancreas Analysis Program (HPAP; https://hpap.pmacs.upenn.edu/about-pancdb; PMID: 36206763; PMID: 31127054). Specifically, we integrated 22 samples generated from human islets, including 6 scRNA-seq samples, 8 snATACseq samples, and 4 multiome samples (Figure 6A). The scRNA-seq and snATAC-seq datasets were obtained from healthy adult human samples, while the multiome dataset included samples from one healthy adult, one young healthy donor, and two type 2 diabetic donors. Some of the datasets were obtained from islet cells in the same donor; Figure 6B shows the overlap of donors between datasets. We believe the HPAP data represent a realistic scenario where all three data types are present, and the goal is to integrate all cells to achieve one uniform cell type clustering result. We integrated the datasets using methods belonging to the ‘unpaired (multiome-split)’ and ‘multiome-guided integration’ categories. However, due to the presence of strong batch effects, we tried our best to modify each method’s default pipeline to limit the impact of technical batch differences. For methods that require z-score standardization, we performed this donor-by-donor and aggregated the scaled data. In situations where highly variable gene selection is needed, we ran highly variable gene selection per donor and aggregated the selected genes across donors by ranking to obtain the most representative genes. More details on specific optimization for each method are in the Supplementary Methods.*

*Results of each integration method are shown in Figure 6C and Supplementary Figure 17A. Since we did not have ground-truth annotation, we analyzed the integration results in three different ways: UMAP projections using the integrated embedding labeled by cluster id (left column) and dataset origin (middle column), and a dot plot showing the average gene expression and percentage of expressed cells for 10 marker genes, one per expected cell type (right column). Overall, Seurat v4, MultiVI, and GLUE all showed good separation of major cell types (Figure 6C). However, Seurat v4 revealed the cleanest result as it produced 10 clusters, each expressing only one cell type marker gene and showing the least amount of co-expression of marker genes for different cell types. The two rare cell populations in the human islet, PPY-expressing gamma cells and GHRL-expressing epsilon cells were also found by Seurat v4. For MultiVI and GLUE, although the broad cell type separation was decent, the GCG-expressing cell population was separated into two clusters, possibly due to technical reasons. Moreover, both methods failed to identify distinct clusters for gamma-cells or epsilon-cells. The results from other integration methods are shown in (Supplementary Figure 17A). We also calculated the same metrics measuring the mixing of batches (scRNA-seq, snATAC-seq, or multiome), and the mixing of cells from different donors (Supplementary Figure 17B). Like previous trends, Seurat v4 showed relatively poorer mixing of cells from different types of assays and donors, while FigR showed better batch mixing than the other methods. This HPAP analysis also allowed us to better evaluate each method’s run time, as there were more than 70,000 cells in this dataset. We again found MultiVI to be the slowest method. Among the ‘unpaired (multiome-split)’ methods, LIGER took the longest while the rest took similar time, also comparable to Seurat v4. ScMoMaT and Cobolt were the fastest, but again, their integration performance was less optimal. ScMoMaT, especially, did not separate the cells into meaningful clusters.*

*Figure 6: Integration of scRNA-seq, snATAC-seq and multiome datasets from the Human Pancreas Analysis Program (HPAP). (A) Illustration of the number of samples for each data type from PANC-DB, the website releasing all HPAP datasets. (B) Overlap of samples from the same human donor between data types. (C) Integration results: UMAP projection using integrated embedding for a select number of methods, colored by cluster ID (left), data type (middle), and dot-plots showing the expression of marker genes per cluster, colored by average expression, sized by the percentage of cells expressing the gene (right). Integration results of the rest of the methods and other evaluation metrics are shown in Supplementary Figure 17.*

9. The running time experiment needs to include cases with a much larger number of cells. 8000 cells is not a large number given the scale of datasets now and in the future. Running time for datasets with 1 million cells will be desirable.

*Response: Thank you for the insightful suggestion, and we wholeheartedly agree that evaluating the runtime in larger datasets would be highly desirable. However, the availability of multiome ground-truth datasets is currently limited. Unfortunately, we were not able to find a multiome dataset that has 1 million cells. The largest dataset we were able to find without significant batch effects is the mouse skin dataset (Ma et al., Cell 2020, PMCID: PMC7669735). Even in this scenario, the evaluation was performed on a maximum of 31,000 cells.*

*In real-world scenarios, it becomes even more challenging to find relevant datasets that encompass scRNA-seq, snATAC-seq, and multiome data generated for the same targeted cell population. We consider ourselves fortunate to have access to the HPAP data, but even in this situation, we were only able to profile 70,000 cells. While it may not reach the scale of 1 million cells, we have made the most of the currently available studies.*

*We appreciate your understanding of the limitations posed by dataset availability and assure you that we have maximized the utilization of existing resources in our evaluations.*

10. The plots need to be improved. They are too monotonous right now. The scale of the y-axis also need to be adjusted to enlarge the actual plotting part. For example Figure 4D NMI the y-axis can be rescaled to (0.5,0.8).

*Response: Thank you for the suggestion. We recognize that there is not much variation in our plots, but the reason is that for each plot, there is a lot of information that we want to show. For example, for Figure 2, each subfigure has nine different methods, splitting into 3 categories with varying numbers of cells. Fixing this format for the rest of the figures is to hope that readers get familiar with reading these plots and thus are able to grasp the key conclusion quickly and efficiently. Moreover, we are demonstrating the HPAP integration result in a different format (Figure 6). We hope you find this addition interesting.*

*Lastly, thanks for bringing up the issue regarding the scale of the y-axis in some of the figures. We have taken your feedback into consideration and made the necessary adjustments to enlarge the actual plotting area and maintain a tight y-axis range.*

Reviewer #2:

Integrating data from different modalities is an important problem in the analysis of single-cell genomic data. In this manuscript, the authors benchmarked seven methods for integrating single-cell RNA-seq (scRNA-seq) and single-cell ATAC-seq (scATAC-seq) data using two datasets with different complexity levels (PBMC and BMMC). They found that single-cell multiome data are helpful for annotating cell types in single modality data. The cell number needs to be large enough for multiome-guided integration to be effective. With fixed cost, increasing cell number is more important than increasing sequence depth. These findings provide a number of useful guidelines for designing future single-cell multiome studies. Therefore, it is worthwhile to have them documented in the literature. I have several questions and comments listed below. I hope that they can help the authors to improve their study and make the conclusions more robust and broadly applicable.

Major issues:

1. It may be useful to evaluate performance and robustness of different methods when the cell types in single-modality scRNA-seq and scATAC-seq are not exactly the same. Will the addition of multiome data help in this case? How will the integration performance depend on cell number of the multiome data? You may simulate data with different degrees of cell type overlap between the two data modalities for this evaluation.

*Response: Thank you for the constructive feedback. We agree completely that it is important to evaluate the performance of integration methods at detecting rarer cell populations that exist in scRNA-seq or snATAC-seq. This would be highly possible in real life as the scRNA-seq is profiling gene expression mainly in the cell cytoplasm while snATAC-seq focuses on chromatin accessibility within the nucleus. Due to the large discrepancy between the two library preparation workflows, it is possible to have cell types exclusive to a certain single-modality dataset. Following this suggestion, we simulated the situation where scRNA-seq and scATAC-seq either have one or two cell types missing, while multiome dataset has the target cell type(s). Below is our description of the simulation and the results shown in Figure 7 (lines 499-552 in the revised manuscript).*

*It is not uncommon to have unique cell types in a certain dataset. This could happen when one of the datasets is generated in a different lab that may have slightly different tissue digestion or single-cell isolation protocol. Moreover, there are intrinsic distinctions between scRNA-seq datasets and the gene expression profiling in the multiome dataset, as the former is single-cell, with mRNA mostly from the cytoplasm, while the latter is single-nucleus, with the mRNA coming from the nucleus. In these situations where the scRNA-seq, snATAC-seq, and multiome have cell types that only exist in two or even one dataset, it is of interest to investigate if the integration algorithms can accurately identify the unique cell populations that are not shared by all three datasets. To this end, we considered two scenarios: (1) scRNA-seq or snATAC-seq do not share the same cell types, e.g. NK cells are found in scRNA-seq and multiome, but not snATAC-seq dataset; (2) multiome dataset does not share the same cell populations as the single-modality datasets, e.g. NK is missing in the multiome dataset, but present scRNA-seq and snATAC. For each situation, we explored varying degrees of cell type overlap, illustrated in Figure 7 and 8.*

*We performed simulations for the above-described scenarios using the PBMC and the SHARE-seq data as the source. The BMMC dataset was not used for this simulation because there were not enough numbers of cells in any single donor, making it challenging to evaluate the impact of rare cells, and the batch effects become strong if all donors are combined. For the PBMC and SHARE-seq simulations, we ensured that the targeted cell types being excluded in certain datasets had at least 50 cells during the clustering step. In the PBMC simulation, the NK cells account for 4.6% of the 10,085 total cells. We considered the situation where there are 1,000 scRNA-seq cells, 1,000 snATAC-seq cells and 1,000, 3,000, or 6,000 multiome cells. We calculated the F1 score for recovering the NK cells in the scRNA-seq dataset and that in the snATAC-seq dataset separately. By comparing the two plots in Figure 7A, we found that the absence of NK cells in the snATAC-seq data (right) led to decrease in the correct identification of NK cells in the scRNA-seq data, as compared to the baseline (left) using the ‘unpaired integration’ methods. If we incorporate the multiome dataset, which contained the NK cells, the F1 score was comparable to the baseline, for both the ‘unpaired (multiome-split)’ and the ‘multiome-guided integration’ methods. Figure 7B shows the F1 score of identifying NK cells in snATAC-seq data when scRNA-seq data. Similar to Figure 7A, the absence of NK cells from scRNA-seq data led to a decrease in recovering NK cells in snATAC-seq data after integration.*

*We generalized this evaluation strategy to the SHARE-seq dataset where we filtered out cell types with less than 500 cells and subset the dataset to one with a total of 10,000 cells, in which ‘Hair Shaft’ (HS) cells account for 10% and ‘endothelial’ cells account for 5% of the total cell population. Using this dataset, we evaluated the situation where snATAC-seq does not have HS cells, endothelial cells, or both and calculated the F1 score for identifying the rare cell population in scRNA-seq (Figure 7C-D). We repeated a similar set-up but with the rare cell population missing in the scRNA-seq data and evaluated the recovery of the rare cell population in snATAC-seq (Figure 7E-F). We also considered the situation when each single-modality dataset has one cell type (scRNA-seq has HS, snATAC-seq has Endo), but the result (data not shown) is similar to the single cell type missing scenarios shown in Figure 7.*

*None of the unpaired integration methods were consistently better than the others when a cell type was missing from one of the single-modality datasets. We observed that, in general, if one single-modality dataset lacks a certain cell type, the identification of this cell type after integration is harder, with the exception of the case shown in Figure 7D. In this case, the baseline performance for some of the unpaired integration methods is comparable to the ‘snATAC missing Endo’ or ‘snATAC missing HS & Endo’ results. One hypothesis is that endothelial cells have very distinct gene expression patterns compared to other cell populations. Therefore, they are distinct enough to be identified as one cluster in the integrated space. ‘Unpaired (multiome-split)’ integration methods and ‘multiome-guided’ integration methods generally achieved a similar accuracy. The top performing method in the ‘unpaired (multiome-split)’ category was GLUE for the SHARE-seq simulated scenarios, but BindSC and FigR performed better for the PBMC simulated cases. Seurat v4 is the best performing method most of the times, except for situations shown in Figure 7E-F.*

*Figure 7 is shown below,*

*Figure 7: Evaluation of rare cell recovery when one of the single-modality datasets miss the rare population of cells. (A-B) F1 score of recovering ‘Natural Killer’ (NK) cells in PBMC-simulated situations. (A) F1 score of recovering NK cells in scRNA-seq at the baseline situation (left) and when snATAC-seq does not have NK cells (right). (B) F1 score of recovering NK cells in snATAC-seq at the baseline situation (left) and when scRNA-seq does not have NK cells (right). (C-F) F1 scores of recovering ‘Hair Shaft’ (HS) cells or ‘Endothelial’ (Endo) cells in SHARE-seq-simulated situations. (C) F1 score of recovering HS cells in scRNA-seq at the baseline situation (left), versus snATAC-seq does not have HS cells (middle), versus snATAC-seq does not have HS and Endo cells (right). (D) F1 score of recovering Endo cells in scRNA-seq at the baseline situation (left), versus snATAC-seq does not have Endo cells (middle), versus snATAC-seq does not have HS and Endo cells (right). (E) F1 score of recovering HS cells in snATAC-seq at the baseline situation (left), versus scRNA-seq does not have HS cells (middle), versus scRNA -seq does not have HS and Endo cells (right). (F) F1 score of recovering Endo cells in snATAC-seq at the baseline situation (left), versus scRNA - seq does not have Endo cells (middle), versus scRNA -seq does not have HS and Endo cells (right). For all box plots, the whisker is 1.5 times the inter-quartile range.*

2. Additionally, it will also be useful to evaluate performance and robustness of different methods when the cell types in multiome data and the cell types in single modality data are not exactly the same. You may simulate data with different degrees of cell type overlap between the multiome and single modality data for this evaluation.

Response: Thank you for the insightful feedback. Again, we completely agree with this comment and thus we added simulations where a cell type is missing in the multiome data, or it is found only in scRNA-seq or snATAC-seq data. Below is our description of the simulation and the results are shown in Figure 8 (lines 553-571 in the revised manuscript).

For the second group of scenarios, we again simulated data using the PBMC and SHARE-seq datasets. Here, our goal was to test the missing cell type situation where multiome did not contain a specific cell population, or if two of the three datasets missed the cell type. Figure 8A-B shows the integration results for scenarios simulated using the PBMC dataset, and the target cell type is NK cells. Comparing the baseline (left) to the ‘Multiome missing NK’ (middle) result, we observe a large decline in the F1 score for Seurat v4 and Cobolt. The other multiome-guided methods are less affected. Results from the unpaired-integration methods are included as a baseline, demonstrating results achieved by the integration of the scRNA-seq and snATAC-seq datasets. The rightmost panel shows an extreme situation where the cell type is only present in scRNA-seq. In this scenario, we observe a decline in performance for the unpaired integration methods as well. We repeated the analysis using the SHARE-seq dataset and simulated with two rare cell populations again, checking if the result would differ depending on the number of cells in the two cell types(Figure 8C-F). Similar to what we observed in the PBMC-based simulations, Seurat v4 was affected the most. This is expected because Seurat v4 is a supervised integration approach; it builds a reference dataset using the multiome cells and then maps the single-modality dataset onto the reference space. Our results suggest that, if there are single-modality specific cell types, they are unlikely to be identified as a unique cell population using the Seurat v4 integration. In this situation, GLUE or other unpaired-integration methods might better preserve those rare cell populations.

Figure 8 is shown below,

Figure 8: Evaluation of rare cell recovery when the multiome dataset or two of the three datasets to be integrated miss the rare population of cells. (A-B) F1 score of recovering ‘Natural Killer’ (NK) cells in PBMC-simulated situations. (A) F1 score of recovering NK cells in scRNA-seq at the baseline situation (left), versus when multiome does not have NK cells (middle), versus when multiome and snATAC-seq do not have NK cells (right). (B) F1 score of recovering NK cells in snATAC-seq at the baseline situation (left), versus when multiome does not have NK cells (middle), versus when multiome and scRNA-seq do not have NK cells (right). (C-F) F1 scores of recovering ‘Hair Shaft’ (HS) cells or ‘Endothelial’ (Endo) cells in SHARE-seq-simulated situations. (C, E) F1 score for recovering HS (C) and Endo (C) in scRNA-seq at the baseline situation (left), versus when multiome does not have the cell type (middle), versus when multiome and snATAC-seq do not have the target cell type (right). (D,F) F1 score for recovering HS (E) and Endo (F) in snATAC-seq at the baseline situation (left), versus when multiome does not have the cell type (middle), versus when multiome and scRNA-seq do not have the target cell type (right). For all box plots, the whisker is 1.5 times the inter-quartile range.

3. Another question of interest is that when multiome data are already available, is it still useful to use previously generated single modality data? To answer this question, it will be helpful to compare the unpaired, unpaired (multiome split), and multiome-guided integration methods to analysis results one would obtain using only the multiome data without any single modality data.

*Response: Thanks for raising this question. Although multiome data are potentially more informative than scRNAseq or snRNA-seq at the per cell level, the previously generated single-modality data remains valuable because these data might contain information that is not captured by the multiome data. A concrete example of this is our experience as the leadership group building the Human Pancreas Analysis Program (HPAP). Initially, scRNA-seq and snATAC-seq data were generated for the earlier donors collected by the program. Subsequently, when the multiome technology became available, the program carefully examined the impact of profiling the two modalities simultaneously and found that it did not greatly affect the data quality. Therefore, HPAP made the decision to generate only the multiome data for future donors.*

*However, due to this historical setup, a significant number of single-modality datasets have already been generated using precious human islet samples. Some of these datasets represent rare situations, such as samples from pediatric donors or early-onset disease cases. It is important to incorporate these single-modality datasets to ensure that cells from all donors are included in the analysis. This is crucial for identifying and studying rarer phenotypes that may be present in a small subset of the available data.*

*Furthermore, when it comes to comparative analysis, such as comparing disease and healthy samples, the number of cells and independent samples plays a pivotal role in deriving robust conclusions. Integrating the single-modality datasets becomes especially critical in the case of datasets generated from human samples. This holds true for large-scale consortia like The Human BioMolecular Atlas Program (HubMap) and the Human Cell Atlas (HCA).*

*Regarding the suggested comparison between the analysis of multiome data using unpaired, unpaired (multiome-split), or multiome guided methods, we think it is an unfair evaluation of the integration methods. The unpaired integration methods are designed to integrate single-modality datasets, so the assumption is that RNA-seq cells and ATAC-seq cells are independent samples from the same cell population. However, for multiome-guided integration methods, they know the pairing between cells. The current best-performing method in the multiome-guided category is Seurat v4, which clusters cells using only the multiome dataset and then project the single-modality dataset onto this multiome reference. In this situation, the multiome was processed without any single-modality datasets.*

*In summary, even when multiome data is available, the previously generated single-modality datasets retain their value, particularly in datasets generated from human samples. Integrating all three data types ensures the inclusion of cells from all donors and enables the exploration of rarer phenotypes.*

4. Line 243 of the manuscript: "Seurat v3 revealed a higher percentage of recovered peak-gene pairs and better F1 score than the measured paired gene expression and chromatin accessibility profile from 1000 cells". The higher percentage of recovered peak-gene pairs is true in Fig 2B, but the better F1 score is not supported by Fig 2B. Indeed, Fig 2B shows that Seurat v3 has a lower F1 score compared to paired data from 1000 cells.

*Response: Thank you for pointing this out, we have revised it (lines 259-262 in the revised manuscript).*

*What’s surprising is that the in-silico prediction profile from Seurat v3 revealed a higher percentage of recovered peak-gene pairs than the measured paired gene expression and chromatin accessibility profile from 1,000 cells, although the F1 score is lower.*

5. Line 246-247: "However, we also note that the predicted profiles only recovered less than 45% of the ground truth list … It is only recovering a small percentage of peak-gene pairs revealed by the experimentally generated multiome dataset". This statement is somewhat misleading. The peak-gene list from unpaired data was obtained using only 1000 cells per data modality, but the ground truth list was obtained using over 10,000 cells. The small percentage (45%) here cannot be used to conclude that imputation-based peak-gene list can only recover a small percentage of true peak-gene list. A fair comparison should be based on the same cell number. For example, if one would have 10,000 single-modality cells and their imputed values, what percentage of ground truth peak-gene list would be recovered where the ground truth is obtained using 10,000 multiome cells.

*Response: Thanks for the constructive feedback, we agree that the statement is immature and misleading. Here is the rephrased version (lines 263-267 in the revised manuscript).*

*Although the predicted profiles are sometimes better than the measured gene expression profiles, here we are only imputing the expression of 1,000 cells, and there are at least 1,000 scRNA-seq cells used for training. Further testing is required to assess the reliability of in-silico imputation when dealing with varying numbers of scRNA-seq cells and multiome cells.*

6. Only two datasets (PBMC, BMMC) are used for this evaluation. Among them, simulations based on the PBMC dataset do not have batch effect between the single-modality and multiome cells, and the "ground truth" cell type label of this dataset is computationally inferred using Seuratv4 (which is also a method to be evaluated) rather than experimentally determined. Thus, many evaluations (e.g. batch effects, cell type annotation) can only be rigorously done using one benchmark dataset (BMMC). If it is possible to compile more benchmark datasets, it would be very useful to see whether the main findings remain consistent across two or more benchmark datasets. If so, the conclusions would be more robust.

*Response: Thank you for the constructive feedback. We agree completely that additional datasets would make the conclusions more robust. Thus, we added two new datasets. Please see the response to Reviewer #1 comment #3 for the details on finding additional ground-truth multi-modal dataset and in the end, we added a mouse-skin dataset generated by SHARE-seq (Ma et al., Cell 2019). We also added another reallife scenario using the HPAP dataset. This dataset includes scRNA-seq, snATAC-seq, and multiome dataset from many human donors. Therefore, there are significant batch effects across samples. We believe this serves as the ultimate challenge for all integration methods. Please see the response to Reviewer #1 comment #8 for more details on how the HPAP datasets were evaluated.*

Minor issues:

7. The first sentence in the Background section (i.e., line 37-39 of the manuscript): "Over the past ten years, hundreds of single-cell RNA-seq … or single-nucleus ATAC-seq … [datasets] have been produced by laboratories worldwide". The word "[datasets]" is missing.

*Response: Thank you for noticing the lack of ‘datasets’. They have been added (lines 37-39).*

*Over the past ten years, hundreds of single-cell RNA-seq (scRNA-seq) (for transcript abundance in single cells) or single-nucleus ATAC-seq (snATAC-seq) (for chromatin accessibility in single nuclei) datasets have been produced by laboratories worldwide…*

8. Line 154: "We evaluated four popular unpaired integration methods (Seurat v4, …)". Here Seurat v4 should be Seurat v3.

*Response: Thank you for catching this typo. We have corrected it in the revised manuscript (line 169).*

*We evaluated five popular unpaired integration methods (Seurat v3, LIGER, FigR, bindSC, GLUE)…*

9. Line 346: (Figure 3D) should be (Figure 3E)

*Response: Thank you for catching this typo. We have corrected it in the revised manuscript (line 389-390).*

*We noted that Seurat v3 performed consistently better than Seurat v4 (Figure 4E).*

**Second round of review**

**Reviewer 1**

The authors have addressed all my comments.

**Reviewer 2**

The authors have satisfactorily answered all my questions.
